# Supplementary material for: Egg-laying and locomotory screens with C. elegans yield a nematode-selective small molecule stimulator of neurotransmitter release
Source: Commun Biol. 2022 Aug 24;5:865. doi: 10.1038/s42003-022-03819-6 (PMC9402605; doi:10.1038/s42003-022-03819-6)
Supplement: Supplementary file 2 — Supplementary Information [file 42003_2022_3819_MOESM2_ESM.pdf]

Supplementary Materials for

Egg-Laying and Locomotory Screens with *C. elegans* Yield a Nematode-Selective Small Molecule Stimulator of Neurotransmitter Release

Sean Harrington, Jessica J. Knox, Andrew R. Burns, Ken-Loon Choo, Aaron Au, Megan Kitner, Cecile Haeberli, Jacob Pyche, Cassandra D'Amata, Yong-Hyun Kim, Jonathan R. Volpatti, Maximillano Guiliani, Jamie Snider, Victoria Wong, Bruna M. Palmeira, Elizabeth M. Redman, Aditya S. Vaidya, John Gilleard, Igor Stagljar, Sean R. Cutler, Daniel Kulke, James J. Dowling, Christopher M. Yip, Jennifer Keiser, Inga Zasada, Mark Lautens, and Peter J. Roy*

*Correspondence to: peter.roy@utoronto.ca

This PDF file includes:

Supplementary Methods (Chemistry)
Supplementary Figs. 1 to 4
Supplementary Table 1
References

Other Supplementary Materials for this manuscript include the following:

Supplementary Movies 1 to 6
Supplementary Data 1 to 7

Supplementary Methods (Chemistry)

General Considerations

Unless otherwise stated, all reactions were set up under inert atmosphere (argon) utilizing glassware (or 2 dram vials) that were flame-dried and cooled under argon purging. Unless otherwise stated, flash column chromatography was performed on Silicycle® Siliaflash® P60, 40-63 µm silica gel. Starting materials and catalysts were purchased from commercial suppliers (Sigma Aldrich, Strem, Alfa Aesar, TCI or Combi-Blocks) and used without further purification unless otherwise stated. All solvents were distilled, purified, and dried according to standard procedures. Reactions were monitored using thin-layer chromatography (TLC) on EMD Silica Gel 60 F254 plates. Visualization of the developed plates was performed under UV light (254 nm) or by immersion in Ceric Ammonium Molybdate (CAM) or Potassium Permanganate ($KMnO_4$) stains.

NMR characterization data was collected at 296 K on a Varian Mercury 300, Varian Mercury 400, Bruker Avance III 400, Agilent DD2 500 (with cold probe), or an Agilent DD2 600 operating at 300, 400, 500, or 600 MHz for ^{1}H NMR, and 75, 100, 125, or 150 MHz for ^{13}C NMR. (Funded by the Canadian Foundation for Innovation, project number 19119, and the Ontario MRI). ^{1}H NMR spectra were internally referenced to the solvent residual signal ($CDCl_3$ = 7.26 ppm) unless otherwise stated. ^{13}C NMR spectra were internally referenced to the residual solvent signal (CDCl3 = 77.16 ppm) unless otherwise stated. ^{19}F NMR spectra were externally referenced to $CFCl_3$. Data for ^{1}H NMR are reported as follows: chemical shift (δ ppm), multiplicity (s = singlet, d =

doublet, t = triplet, q = quartet, m = multiplet, br = broad), coupling constant (Hz), integration.

Melting point (mp) ranges were determined on a Fisher-Johns® Melting Point Apparatus and are reported uncorrected.

Infrared (IR) spectra were acquired using a Shimadzu FTIR-8400S FT-IR spectrometer as thin films ($CHCl_3$ or CH_2Cl_2) or neat on NaCl plates. Data is presented in wavenumbers (v_{max}, cm^{-1}).

High Resolution Mass Spectra (HRMS) were obtained on a micromass 70S-250 spectrometer (EI) or an AB SCIEX QSTAR® Mass Spectrometer (ESI) or a JEOL® AccuTOF medel JMS-T1000LC mass spectrometer equipped with an IONICS® Direct Analysis in real Time (DART) ion source at Advanced Instrumentation For Molecular Structure (AIMS) in the Department of Chemistry at the University of Toronto. Where ESI+ was employed, the values correspond to the ionic species of interest and the given ionic formula includes the charging agent (H^+ or Na^+); both the measured and calculated values are corrected for the mass of the electron and are reported as m/z. The DART-MS accurate mass report is generated using the *Elemental Composition Estimation* feature as implemented in the JEOL Mass Centre software package. Where DART was employed, the measured values correspond to the neutral species of interest and the given molecular formula includes the charging agent (H^+ or NH_4^+); the measured and calculated values are not corrected for the mass of the electron and are reported as neutral masses.

General Procedures for the Synthesis

General Procedure A – Wittig Reaction

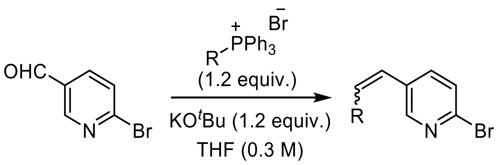

The phosphonium salt (1.2 equiv.) was first dissolved in THF (0.3 M). KO^{t}Bu (1.2 equiv.) was then added and the mixture was stirred at room temperature for 30 minutes. The pyridinecarboxaldehyde (1 equiv.) was then added in three portions and the reaction was refluxed at 70 °C for 3 hours. The mixture was then allowed to cool to room temperature and was filtered through a Celite pad eluting with pentanes. The filtrate was concentrated in vacuo and the product (mixture of isomers) was purified by flash column chromatography, eluting with a mixture of EtOAc:pentanes.

General Procedure B – Suzuki Reaction[1]

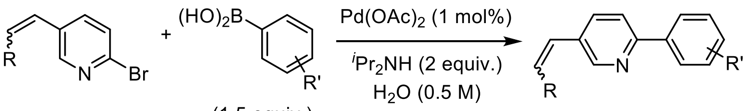

Substituted aryl boronic acid (1.5 equiv.) and $Pd(OAc)_2$ (1 mol%) were added to a mixture of the substituted bromopyridine (1 equiv.) in water (0.5 M). ^{i}Pr$_2$NH (2 equiv.) was then added and the reaction was refluxed at 100 °C for 16 hours. The mixture was allowed to cool to room temperature and brine was added. The aqueous phase was extracted with ethyl acetate. The combined organics was washed with brine, dried with

MgSO4, and concentrated in vacuo. The product was purified by flash column chromatography, eluting with a mixture of EtOAc:pentanes.

General Procedure C – Hydrogenation

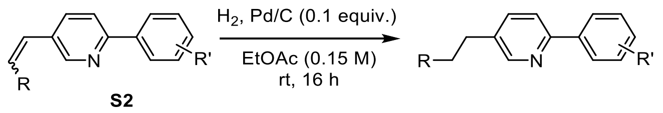

The vinylbiarene was dissolved in EtOAc (0.15 M). Pd/C (3% Pd, total 10 mol% Pd used) was added. Three cycles of evacuation and backfill with argon, followed by H_2 from a balloon was carried out. The reaction was stirred at room temperature under a H_2 atmosphere (balloon) for 16 hours. The contents of the flask were filtered over a Celite pad eluting with EtOAc and concentrated *in vacuo*. The product was purified by flash column chromatography, eluting with a mixture of EtOAc:pentanes.

2-(4-((difluoromethyl)thio)phenyl)-5-propylpyridine [nementin-12-5]

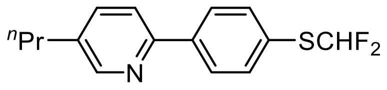

nementin-12-5 was synthesized according to General Procedure A, B, and C using 6-bromonicotinaldehyde, ethyltriphenylphosphonium bromide, and (4-((difluoromethyl)thio)phenyl)boronic acid with an overall yield of 21% as a pale yellow oil. **^{1}H NMR** (500 MHz, $CDCl_3$) δ 8.53 (s, 1H), 8.04 – 7.95 (m, 2H), 7.69 – 7.62 (m, 3H), 7.60 – 7.53 (m, 1H), 6.86 (t, J = 56.9 Hz, 1H), 2.64 (d, J = 8.4 Hz, 2H), 1.76 – 1.64 (m, 2H), 0.98 (t, J = 7.3 Hz, 3H). **^{13}C NMR** (125 MHz, $CDCl_3$) δ 153.7, 150.0, 140.8, 137.0, 136.8, 135.4 (d, J = 1.2 Hz), 127.5, 126.3 (t, J = 3.0 Hz), 121.0 (t, J = 275.3 Hz), 120.2, 34.7, 24.2, 13.7. **^{19}F NMR** (375 MHz, $CDCl_3$) δ -91.2 (d, J = 57.0 Hz). **IR** (thin film): 2962, 2932, 2871, 1554, 1472,

1319, 1068, 1036, 817 cm^{-1}. **HRMS** (DART-TOF+): mass [M+H] calc'd for $C_{15}H_{16}F_2NS$ 280.0972; found 280.0974.

4-(difluoromethoxy)-4'-propyl-1,1'-biphenyl [nementin-12-6]

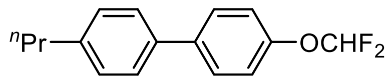

nementin-12-6 was synthesized according to General Procedure A, B, and C using 4-bromobenzaldehyde, ethyltriphenylphosphonium bromide, and (4-(difluoromethoxy)phenyl)boronic acid with an overall yield of 48% as a white solid. **^{1}H NMR** (500 MHz, $CDCl_3$) δ 7.61 – 7.53 (m, 2H), 7.50 – 7.45 (m, 2H), 7.29 – 7.24 (m, 2H), 7.21 – 7.16 (m, 2H), 6.55 (t, J = 74.0 Hz, 1H), 2.64 (t, J = 8.5 Hz, 2H), 1.75 – 1.63 (m, 2H), 0.99 (t, J = 7.3 Hz, 3H). **^{13}C NMR** (125 MHz, $CDCl_3$) δ 150.4 (t, J = 2.8 Hz), 142.1, 138.6, 137.4, 129.0, 128.3, 126.8, 119.8, 116.0 (t, J = 259.5 Hz), 37.7, 24.5, 13.9. **^{19}F NMR** (375 MHz, $CDCl_3$) δ -80.6 (d, J = 74.5 Hz). **IR** (thin film): 2961, 2931, 1497, 1381, 1224, 1130, 1049, 909, 734 cm^{-1}. **HRMS** (DART-TOF+): mass [M] calc'd for $C_{16}H_{16}F_2O$ 262.1169; found 262.1162. **mp** 74-75 °C.

5-(4-(difluoromethoxy)phenyl)-2-propylpyridine [nementin-12-7]

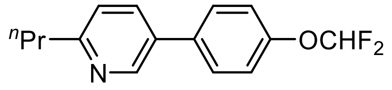

nementin-12-7 was synthesized according to General Procedure A, B, and C using 5-bromopicolinaldehyde, ethyltriphenylphosphonium bromide, and (4-(difluoromethoxy)phenyl)boronic acid with an overall yield of 16% as a white solid. **^{1}H NMR** (500 MHz, $CDCl_3$) δ 8.75 – 8.68 (m, 1H), 7.77 – 7.72 (m, 1H), 7.59 – 7.53 (m, 2H), 7.25 – 7.19 (m, 3H), 6.56 (t, J = 73.7 Hz, 1H), 2.81 (t, J = 7.6 Hz, 2H), 1.87 – 1.72 (m, 2H), 1.00 (t, J = 7.4 Hz, 3H). **^{13}C NMR** (125

MHz, $CDCl_3$) δ 161.4, 150.9 (d, $J = 3.2$ Hz), 147.4, 135.3, 134.5, 132.8, 128.4, 122.7, 120.1, 115.8 (t, $J = 260.3$ Hz), 40.0, 23.1, 13.9. **^{19}F NMR** (375 MHz, $CDCl_3$) δ -80.9 (d, $J = 73.3$ Hz). **IR** (thin film): 2962, 1599, 1483, 1224, 1127,1046, 819 cm^{-1}. **HRMS** (DART-TOF+): mass [M+H] calc'd for $C_{15}H_{16}F_2ON$ 264.1200; found 264.1194. **mp** 47-48 °C.

5-(difluoromethoxy)-2-(4-propylphenyl)pyridine [nementin-12-8]

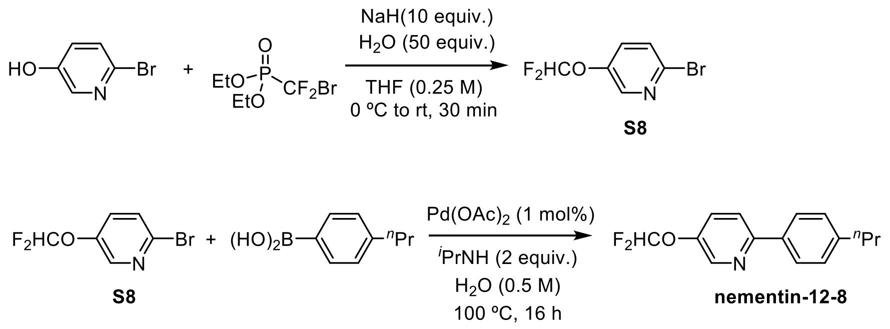

2-bromo-5-(difluoromethoxy)pyridine (S8): The procedure was adapted from Wu and Wu *et al.*[2] 6-bromopyridin-3-ol (348 mg, 2 mmol, 1 equiv.) was dissolved in THF (8 mL, 0.25 M) and cooled to 0 °C. NaH (800 mg, 20 mmol, 60%, 10 equiv.) was added and the mixture was stirred at 0 °C for 30 minutes. H_2O (1.80 mL, 100 mmol, 50 equiv.) was added dropwise and the mixture was stirred at 0 °C for 10 minutes. Diethyl (bromodifluoromethyl)phosphonate (0.71 mL, 4 mmol, 2 equiv.) was added and the reaction was allowed to stir from 0 °C to room temperature for 30 minutes. H_2O was added and the aqueous phase was extracted with EtOAc. The combined organics was washed with brine, dried with $MgSO_4$, and concentrated in vacuo. The crude mixture was purified by flash column chromatography, eluting with 5% (v/v) EtOAc:pentanes to give S8 (161 mg, 0.72 mmol, 36%).

5-(difluoromethoxy)-2-(4-propylphenyl)pyridine [nementin-12-8]: Suzuki reaction was carried out to couple S8 (161 mg, 0.72 mmol, 1 equiv.) and (4-propylphenyl)boronic acid (177 mg, 1.08 mmol, 1.5 equiv.) according to General Procedure B to give **nementin-12-8** (140 mg, 0.53 mmol, 74%) as a white solid. **^{1}H NMR** (500 MHz, $CDCl_3$) δ 8.55 – 8.51 (m, 1H), 7.90 – 7.84 (m, 2H), 7.73 – 7.69 (m, 1H), 7.56 – 7.49 (m, 1H), 7.33 – 7.27 (m, 2H), 6.57 (t, J = 72.9 Hz, 1H), 2.65 (t, J = 7.3 Hz, 2H), 1.74 – 1.64 (m, 2H), 0.97 (t, J = 7.3 Hz, 3H). **^{13}C NMR** (125 MHz, $CDCl_3$) δ 154.9, 146.0 (t, J = 2.7 Hz), 143.9, 141.8, 135.8, 129.0, 128.2, 126.7, 120.6, 115.4 (t, J = 263.2 Hz), 37.8, 24.4, 13.8. **^{19}F NMR** (375 MHz, $CDCl_3$) δ -81.2 (d, J = 72.7 Hz). **IR** (thin film): 2961, 2930, 1471, 1379, 1215, 1118, 1047, 831 cm^{-1}. **HRMS** (DART-TOF+): mass [M+H] calc'd for $C_{15}H_{16}F_2ON$ 264.1200; found 264.1197. **mp** 25-26 °C.

2-(difluoromethoxy)-5-(4-propylphenyl)pyridine [nementin-12-9]

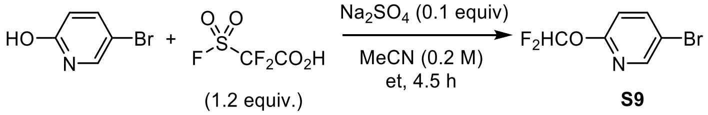

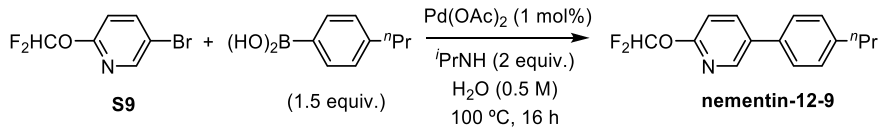

5-bromo-2-(difluoromethoxy)pyridine (S9): The procedure was adapted from Ando *et al.*[3]. 5-bromopyridin-2-ol (870 mg, 5 mmol, 1 equiv.) was dissolved in MeCN (25 mL, 0.2 M). 2,2-difluoro-2-(fluorosulfonyl)acetic acid (0.67 mL, 6 mmol, 1.2 equiv.) was

added, followed by Na_2SO_4 (7 mg, 0.5 mmol, 0.1 equiv.). The reaction was quenched with saturated $NaHCO_{3(aq)}$ solution and extracted with EtOAC. The combined organics was washed with brine, dried with $MgSO_4$, and concentrated in vacuo. The crude mixture was purified by flash column chromatography, eluting with a 5 to 10% (v/v) EtOAc:pentanes gradient to give S9 (522 mg, 2.3 mmol, 47%).

2-(difluoromethoxy)-5-(4-propylphenyl)pyridine (nementin-12-9): Suzuki reaction was carried out to couple S9 (112 mg, 0.5 mmol, 1 equiv.) and (4-propylphenyl)boronic acid (123 mg, 0.75 mmol, 1.5 equiv.) according to General Procedure B to give **nementin-12-9** (114 mg, 0.433 mmol, 87%) as an amorphous solid. **^{1}H NMR** (500 MHz, $CDCl_3$) δ 8.41 – 8.37 (m, 1H), 7.93 – 7.88 (m, 1H), 7.50 (t, *J* = 73.1 Hz, 1H), 7.46 – 7.42 (m, 2H), 7.31 – 7.27 (m, 2H), 6.96 (dd, *J* = 8.5, 0.7 Hz, 1H), 2.64 (t, *J* = 7.6 Hz, 2H), 1.74 – 1.62 (m, 2H), 0.98 (t, *J* = 7.3 Hz, 3H). **^{13}C NMR** (125 MHz, $CDCl_3$) δ 158.2 (t, *J* = 3.7 Hz), 144.9, 142.8, 138.6, 134.3, 133.6, 129.2, 126.7, 114.1 (t, *J* = 255.2 Hz), 111.6 – 111.0 (m), 37.7, 24.5, 13.8. **^{19}F NMR** (375 MHz, $CDCl_3$) δ -88.6 (d, *J* = 73.2 Hz). **IR** (thin film): 3362, 2962, 1601, 1478, 1347, 1258, 1129, 1103, 1072, 831 cm^{-1}. **HRMS** (DART-TOF+): mass [M+H] calc'd for $C_{15}H_{16}F_2ON$ 264.1200; found 264.1200.

2-(4-(difluoromethoxy)phenyl)-5-ethylpyridine [nementin-12-10]

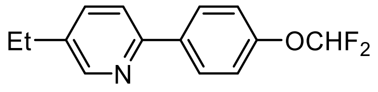

nementin-12-10 was synthesized according to General Procedure A, B, and C using 6-bromonicotinaldehyde, methyltriphenylphosphonium bromide, and (4-(difluoromethoxy)phenyl)boronic acid with

an overall yield of 4% as a white solid. **^{1}H NMR** (500 MHz, $CDCl_3$) δ 8.53 (s, 1H), 8.01 – 7.91 (m, 2H), 7.66 – 7.51 (m, 2H), 7.23 – 7.17 (m, 2H), 6.56 (t, *J* = 73.9 Hz, 1H), 2.69 (q, *J* = 7.6 Hz, 2H), 1.29 (t, *J* = 7.6 Hz, 3H). **^{13}C NMR** (125 MHz, $CDCl_3$) δ 153.8, 151.6 (t, *J* = 2.8 Hz), 149.5 – 149.3 (m), 137.9, 136.7, 136.2, 119.9, 119.5, 115.9 (td, *J* = 259.3, 3.1 Hz), 25.8, 15.3. **^{19}F NMR** (375 MHz, $CDCl_3$) δ -80.8 (d, *J* = 73.8 Hz). **IR** (thin film): 2967, 1478, 1381, 1221, 1129, 1052, 908, 733, 665 cm^{-1}. **HRMS** (DART-TOF+): mass [M+H] calc'd for $C_{14}H_{14}F_2ON$ 250.1044; found 250.1049. **mp** 32-33 °C.

2-(4-(difluoromethoxy)phenyl)-5-methylpyridine [nementin-12-11]

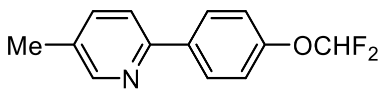

nementin-12-11 was synthesized according to General Procedure B using 2-bromo-5-methylpyridine and (4-(difluoromethoxy)phenyl)boronic acid with a yield of 14% as a white solid. **^{1}H NMR** (500 MHz, $CDCl_3$) δ 8.51 (s, 1H), 8.00 – 7.95 (m, 2H), 7.64 – 7.50 (m, 2H), 7.23 – 7.18 (m, 2H), 6.56 (t, *J* = 73.9 Hz, 1H), 2.37 (s, 3H). **^{13}C NMR** (125 MHz, $CDCl_3$) δ 153.6, 151.6 (t, *J* = 2.8 Hz), 137.4, 136.7, 131.8, 128.1, 119.8, 119.5, 115.9 (t, *J* = 259.7 Hz), 18.1. **^{19}F NMR** (375 MHz, $CDCl_3$) δ -80.8 (d, *J* = 73.9 Hz). **IR** (thin film): 3004, 1605, 1477, 1220, 1123, 1-42, 823 cm^{-1}. **HRMS** (DART-TOF+): mass [M+H] calc'd for $C_{13}H_{12}F_2ON$ 236.0882; found 236.0884. **mp** 67-69 °C.

2-(4-(difluoromethoxy)phenyl)pyridine [nementin-12-12]

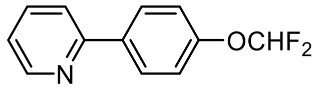

nementin-12-12 was synthesized according to General Procedure B using 2-bromopyridine and (4-

(difluoromethoxy)phenyl)boronic acid with a yield of 31% as a white solid. **^{1}H NMR** (500 MHz, $CDCl_3$) δ 8.71 – 8.63 (m, 1H), 8.04 – 7.98 (m, 2H), 7.79 – 7.73 (m, 1H), 7.72 – 7.68 (m, 1H), 7.26 – 7.19 (m, 3H), 6.57 (t, *J* = 73.8 Hz, 1H). **^{13}C NMR** (125 MHz, $CDCl_3$) δ 156.3, 151.9, 149.7, 136.8, 136.6, 128.4, 122.2, 120.3, 119.5, 115.8 (td, *J* = 260.0, 2.8 Hz). **^{19}F NMR** (375 MHz, $CDCl_3$) δ -80.8 (d, *J* = 74.0 Hz). **IR** (thin film): 3368, 2924, 1592, 1468, 1437, 1382, 1222, 1178, 1043, 780 cm^{-1}. **HRMS** (DART-TOF+): mass [M+H] calc'd for $C_{15}H_{16}F_2ON$ 222.0731; found 222.0724. **mp** 35-36 °C.

2-(4-(difluoromethoxy)phenyl)-5-(prop-1-yn-1-yl)pyridine [nementin-12-14]

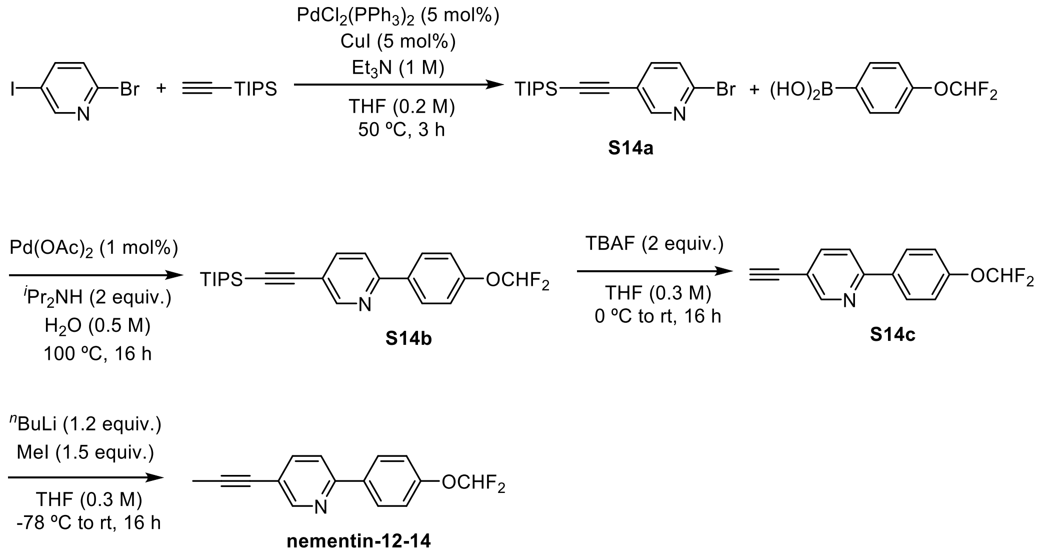

2-bromo-5-((triisopropylsilyl)ethynyl)pyridine (S14a): 2-bromo-5-iodopyridine (283 mg, 1 mmol, 1 equiv.), $PdCl_2(PPh_3)_2$ (35 mg, 0.05 mmol, 5 mol%), and CuI (10 mg, 0.05 mmol, 5 mol%) were weighed into a flame-dried round bottom flask. The contents were purged under nitrogen for 5 minutes. THF (6.0 mL, 0.2 M) was then added to the flask. Et_3N (1 mL, 1 M) and ethynyltriisopropylsilane (0.27 mL, 1.2 mmol, 1.2 equiv.) were

added subsequently. The reaction was stirred at 50 °C for 3 hours. The mixture was cooled to room temperature and then filtered through celite, eluting with EtOAc. The filtrate was concentrated in vacuo and the crude mixture was purified by flash column chromatography, eluting with 10% (v/v) EtOAc:pentanes to give S14a (237 mg, 0.70 mmol, 70%)

2-(4-(difluoromethoxy)phenyl)-5-((triisopropylsilyl)ethynyl)pyridine (S14b): Suzuki reaction was carried out to couple S14a (237 mg, 0.70 mmol, 1 equiv.) and (4-(difluoromethoxy)phenyl)boronic acid (197 mg, 1.05 mmol, 1.5 equiv.) according to General Procedure B to give S14b (162 mg, 0.40 mmol, 58%).

2-(4-(difluoromethoxy)phenyl)-5-ethynylpyridine (S14c): S14b (162 mg, 0.4 mmol, 1 equiv.) was dissolved in THF (1.3 mL, 0.3 M) and cooled to 0 °C. TBAF (0.8 mL, 0.8 mmol, 1 M in THF, 2 equiv.) was added dropwise. The reaction was allowed to stir from 0 °C to room temperature for 16 hours. H_2O was added and the aqueous phase was extracted with EtOAc. The combined organics was washed with brine, dried with $MgSO_4$, and concentrated in vacuo. The crude mixture was purified by flash column chromatography, eluting with a 5 to 10% (v/v) EtOAc:pentanes gradient to give S14c (77 mg, 0.31 mmol, 78%).

2-(4-(difluoromethoxy)phenyl)-5-(prop-1-yn-1-yl)pyridine (nementin-12-14): S14c (77 mg, 0.31 mmol, 1 equiv.) was dissolved in THF (1.0 mL, 0.3 M) and cooled to -78 °C. ^{n}BuLi (0.15 mL, 0.37 mmol, 2.5 M in hexane, 1.2 equiv.) was added dropwise. The mixture was stirred at -78 °C for 1 hour. Iodomethane (0.03 mL, 0.47 mmol, 1.5 equiv.) was added dropwise. The reaction was allowed to stir from -78 °C to room

temperature for 16 hours. The reaction was quenched with saturated $NH_4Cl_{(aq)}$ solution and extracted with EtOAc. The combined organics was washed with brine, dried with $MgSO_4$, and concentrated in vacuo. The crude mixture was purified by flash column chromatography, eluting with a 2.5 to 5% (v/v) EtOAc:pentanes gradient to give **nementin-12-14** (28 mg, 0.11 mmol, 35%) as a white solid. **^{1}H NMR** (500 MHz, $CDCl_3$) δ 8.68 – 8.67 (m, 1H), 8.02 – 7.96 (m, 2H), 7.75 – 7.70 (m, 1H), 7.65 – 7.59 (m, 1H), 7.24 – 7.17 (m, 2H), 6.56 (t, *J* = 73.8 Hz, 1H), 2.10 (s, 3H). **^{13}C NMR** (125 MHz, $CDCl_3$) δ 154.3, 152.2, 152.0 (t, *J* = 2.8 Hz), 139.2, 136.0, 119.6, 119.5, 119.3, 115.8 (t, *J* = 260.0 Hz), 90.0, 76.6, 4.5. **^{19}F NMR** (375 MHz, $CDCl_3$) δ -80.9 (d, *J* = 72.9 Hz). **IR** (thin film): 2932, 2256, 2221, 1588, 1475, 1378, 1230, 1129, 1041, 828 cm^{-1}. **HRMS** (DART-TOF+): mass [M+H] calc'd for $C_{15}H_{12}F_2ON$ 260.0887; found 260.0879. **mp** 68-70 °C.

(6-(4-(difluoromethoxy)phenyl)pyridin-3-yl)methanamine [nementin-12-17]

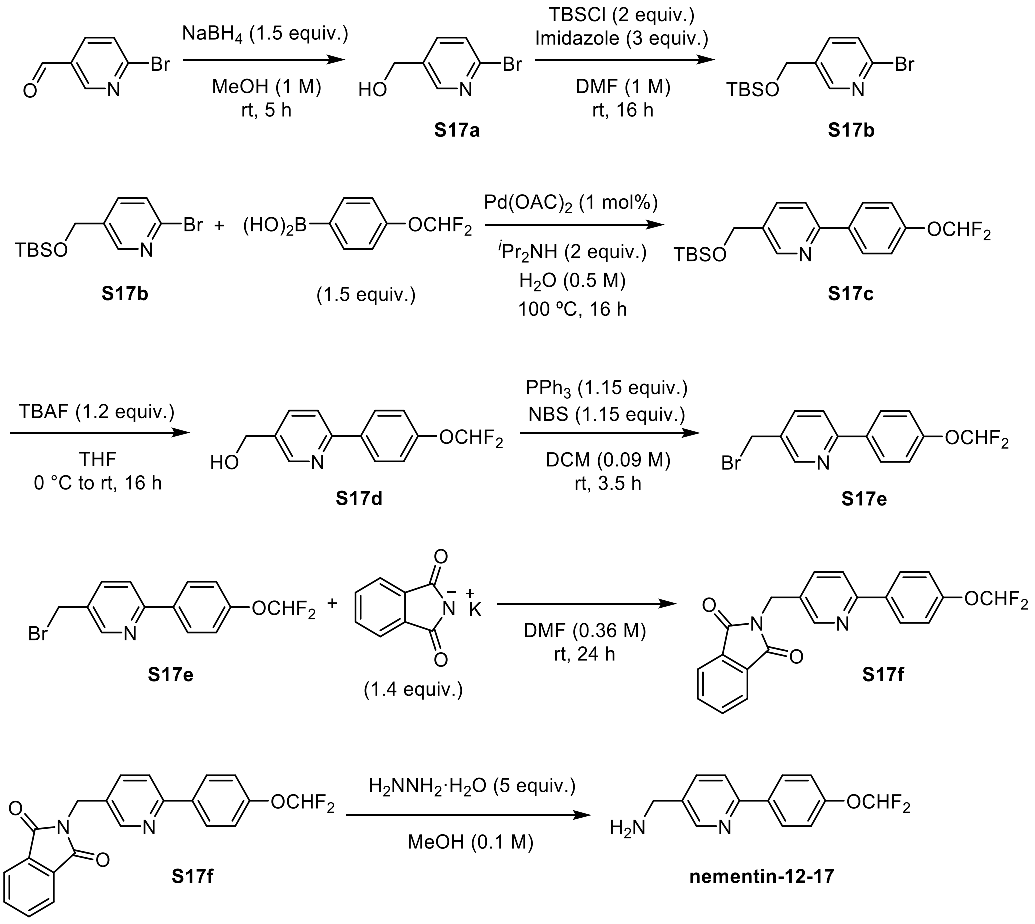

(6-bromopyridin-3-yl)methanol (S17a): 6-bromonicotinaldehyde (1.302 g, 7 mmol, 1 equiv.) was dissolved in MeOH (7 mL, 1 M). $NaBH_4$ (397 mg, 10.5 mmol, 1.5 equiv.) was added in three portions. The reaction was stirred at room temperature for 5 hours. The mixture was then cooled to 0 °C and 1 M HCl was added dropwise until bubbling seized, followed with dilution of the mixture with H_2O. The aqueous phase was extracted with EtOAc. The combined organics was washed with brine, dried with $MgSO_4$, and concentrated in vacuo. The crude mixture was purified by flash column

chromatography, eluting with a 50 to 60% (v/v) EtOAc:pentanes gradient to give S17a (1.286 g, 6.84 mmol, 98%).

2-bromo-5-(((tert-butyldimethylsilyl)oxy)methyl)pyridine (S17b): S17a (884 mg, 4.7 mmol, 1 equiv.) was dissolved in DMF (4.7 mL, 1 M) and cooled to 0 °C. TBSCl (1.416 g, 9.4 mmol, 2 equiv.) was added followed by imidazole (960 mg, 14.1 mmol, 3 equiv.). The reaction was allowed to stir from 0 °C to room temperature for 16 hours. H_2O was added to the mixture and the aqueous phase was extracted with EtOAc. The combined organics was washed with brine, dried with $MgSO_4$, and concentrated in vacuo. The crude mixture was purified by flash column chromatography, eluting with a 1 to 2% (v/v) EtOAc:pentanes gradient to give S17b (1.095 g, 3.63 mmol, 77%).

5-(((tert-butyldimethylsilyl)oxy)methyl)-2-(4-(difluoromethoxy)phenyl)pyridine (S17c): Suzuki reaction was carried out to couple S17b (1.090 g, 3.63 mmol, 1 equiv.) and (4-(difluoromethoxy)phenyl)boronic acid (1.020 g, 5.45 mmol, 1.5 equiv.) according to General Procedure B to give S17c (752 mg, 1.98 mmol, 54%).

(6-(4-(difluoromethoxy)phenyl)pyridin-3-yl)methanol (S17d): **S17c** (752 mg, 1.98 mmol, 1 equiv.) was dissolved in THF (6.6 mL, 0.3 M) and cooled to 0 °C. TBAF (2.38 mL, 2.38 mmol, 1 M in THF, 1.2 equiv.) was added dropwise. The reaction was allowed to stir from 0 °C to room temperature for 16 hours. H_2O was added to the mixture and the aqueous phase was extracted with EtOAc. The combined organics was washed with brine, dried with $MgSO_4$, and concentrated in vacuo. The crude mixture was purified by flash column chromatography, eluting with a 50 to 60% (v/v) EtOAc:pentanes gradient to give S17d (363 mg, 1.45 mmol, 73%).

5-(bromomethyl)-2-(4-(difluoromethoxy)phenyl)pyridine (S17e): S17d (363 mg, 1.45 mmol, 1 equiv.) was dissolved in DCM (16.11 mL, 0.09 M). PPh_3 (438 mg, 1.67 mmol, 1.15 equiv.) followed by NBS (297 mg, 1.67 mmol, 1.15 equiv.) were added. The reaction was stirred at room temperature for 3.5 hours. H_2O was added to the mixture and the aqueous phase was extracted with EtOAc. The combined organics was washed with brine, dried with $MgSO_4$, and concentrated in vacuo. The crude mixture was purified by flash column chromatography, eluting with a 15% (v/v) EtOAc:pentanes to give S17e (350 mg, 1.11 mmol, 77%).

2-((6-(4-(difluoromethoxy)phenyl)pyridin-3-yl)methyl)isoindoline-1,3-dione (S17f): S17e (349 mg, 1.11 mmol, 1 equiv.) was dissolved in DMF (3.1 mL, 0.36 M). Phthalimide potassium salt (438 mg, 1.67 mmol, 1.15 equiv.) was added. The reaction was stirred at room temperature for 24 hours. H_2O was added to the mixture and the aqueous phase was extracted with EtOAc. The combined organics was washed with brine, dried with $MgSO_4$, and concentrated in vacuo. The crude mixture was purified by flash column chromatography, eluting with a 20 to 25% (v/v) EtOAc:pentanes gradient to give S17f (262 mg, 0.69 mmol, 62%).

(6-(4-(difluoromethoxy)phenyl)pyridin-3-yl)methanamine (nementin-12-17): S17f (262 mg, 0.69 mmol, 1 equiv.) was dissolved in MeOH (6.9 mL, 0.1 M). Hydrazine hydrate (0.26 mL, 3.56 mmol, 50-60%, 5 equiv.) was added. The reaction was refluxed at 70 °C for 4 hours. The mixture was cooled to room temperature and filtered through Celite, eluting with MeOH. The filtrate was concentrated in vacuo. H_2O (7 mL) was

added, followed by 1 M KOH (1.75 mL). The aqueous phase was extracted with DCM. The combined organics was dried with $MgSO_4$ and then concentrated in vacuo to give **nementin-12-17** (131 mg, 0.53 mmol, 76%) as a white solid. **^{1}H NMR** (500 MHz, $CDCl_3$) δ 8.64 – 8.58 (m, 1H), 8.01 – 7.96 (m, 2H), 7.79 – 7.71 (m, 1H), 7.71 – 7.64 (m, 1H), 7.23 – 7.18 (m, 2H), 6.56 (t, J = 73.8 Hz, 1H), 3.95 (s, 2H), 1.52 (br, 2H). **^{13}C NMR** (125 MHz, $CDCl_3$) δ 155.0, 151.8 (t, J = 2.8 Hz), 148.8, 136.8, 136.5, 135.8, 128.3, 120.1, 119.5, 115.8 (t, J = 259.9 Hz), 43.7. **^{19}F NMR** (375 MHz, $CDCl_3$) δ -80.8 (d, J = 73.3 Hz). **IR** (thin film): 3265, 1591, 1478, 1381, 1231, 1125, 1037, 783 cm^{-1}. **HRMS** (DART-TOF+): mass [M+H] calc'd for $C_{13}H_{13}F_2ON_2$ 251.0991; found 251.0988. **mp** 41-43 °C.

2-(4-(difluoromethoxy)phenyl)-6-propylpyridine [nementin-12-20]

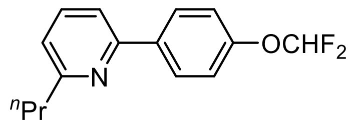

nementin-12-20 was synthesized according to General Procedure A, B, and C using 6-bromopicolinaldehyde, ethyltriphenylphosphonium bromide, and (4-(difluoromethoxy)phenyl)boronic acid with an overall yield of 18% as a pale yellow oil. **^{1}H NMR** (500 MHz, $CDCl_3$) δ 8.04 – 7.99 (m, 2H), 7.68 – 7.62 (m, 1H), 7.51 – 7.47 (m, 1H), 7.24 – 7.15 (m, 2H), 7.11 – 7.06 (m, 1H), 6.55 (t, J = 73.9 Hz, 1H), 2.83 (t, J = 7.9 Hz, 2H), 1.87 – 1.77 (m, 2H), 1.01 (t, J = 7.4 Hz, 3H). **^{13}C NMR** (125 MHz, $CDCl_3$) δ 162.3, 155.6, 151.7 (t, J = 2.8 Hz), 137.2, 136.8, 128.5, 121.2, 119.5, 117.4, 115.9 (dd, J = 260.1, 3.2 Hz), 40.5, 22.9, 13.9. **^{19}F NMR** (375 MHz, $CDCl_3$) δ -80.7 (d, J = 74.3 Hz). **IR** (thin film): cm^{-1}. **HRMS** (DART-TOF+): mass [M+H] calc'd for $C_{15}H_{16}F_2ON$ 264.1200; found 264.1197.

2-(4-(difluoromethoxy)phenyl)-4-propylpyridine [nementin-12-21]

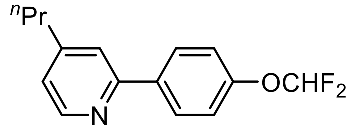

nementin-12-21(G) was synthesized according to General Procedure A, B, and C using 2-bromoisonicotinaldehyde, ethyltriphenylphosphonium bromide, and (4-(difluoromethoxy)phenyl)boronic acid with an overall yield of 9% as an amorphous solid. **^{1}H NMR** (500 MHz, $CDCl_3$) δ 8.55 (d, *J* = 5.0 Hz, 1H), 8.01 – 7.95 (m, 2H), 7.51 – 7.48 (m, 1H), 7.23 – 7.16 (m, 2H), 7.07 – 7.03 (m, 1H), 6.56 (t, *J* = 73.9 Hz, 1H), 2.64 (t, *J* = 7.7 Hz, 2H), 1.77 – 1.64 (m, 2H), 0.98 (t, *J* = 7.3 Hz, 3H). **^{13}C NMR** (125 MHz, $CDCl_3$) δ 156.2, 152.4, 151.8 (t, *J* = 2.8 Hz), 149.6 – 149.4 (m), 136.9, 128.5 (d, *J* = 1.2 Hz), 122.6, 120.6 (t, *J* = 2.1 Hz), 119.4, 115.9 (td, *J* = 260.0, 3.8 Hz), 37.5, 23.6, 13.7. **^{19}F NMR** (375 MHz, $CDCl_3$) δ -80.8 (d, *J* = 73.3 Hz). **IR** (thin film): 2961, 1605, 1222, 1130, 1050, 909, 733 cm^{-1}. **HRMS** (DART-TOF+): mass [M+H] calc'd for $C_{15}H_{16}F_2ON$ 264.1200; found 264.1205.

2-(4-(difluoromethoxy)phenyl)-3-propylpyridine [nementin-12-22]

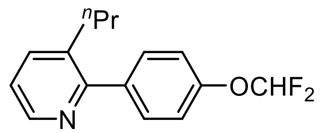

nementin-12-22 was synthesized according to General Procedure A, B, and C using 2-bromonicotinaldehyde, ethyltriphenylphosphonium bromide, and (4-(difluoromethoxy)phenyl)boronic acid with an overall yield of 6% as a pale yellow oil. **^{1}H NMR** (500 MHz, $CDCl_3$) δ 8.53 – 8.48 (m, 1H), 7.64 – 7.58 (m, 1H), 7.51 – 7.46 (m, 2H), 7.24 – 7.16 (m, 3H), 6.56 (t, *J* = 73.9 Hz, 1H), 2.61 (t, *J* = 7.7 Hz, 2H), 1.60 – 1.46 (m, 2H), 0.86 (t, *J* = 7.3 Hz, 3H). **^{13}C NMR** (125 MHz, $CDCl_3$) δ 157.6, 150.9 (t, *J* = 2.8 Hz), 146.9 – 146.7 (m), 138.0, 137.4, 135.5, 130.4, 122.4, 119.1, 116.0 (td, *J* = 259.1, 2.6 Hz), 34.4, 24.0, 13.9. **^{19}F NMR** (375 MHz,

CDCl3) δ -80.7 (d, *J* = 73.9 Hz). **IR** (thin film): 3367, 2962, 2873, 1610, 1511, 1435, 1382, 1223, 1179, 1127, 1045, 843, 783, 665 cm^{-1}. **HRMS** (DART-TOF+): mass [M+H] calc'd for $C_{15}H_{16}F_2ON$ 264.1200; found 264.1193.

2-(3-(difluoromethoxy)phenyl)-5-propylpyridine [nementin-12-23]

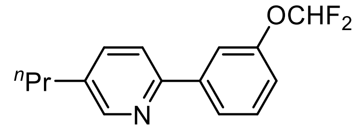

nementin-12-23 was synthesized according to General Procedure A, B, and C using 6-bromonicotinaldehyde, ethyltriphenylphosphonium bromide, and (3-(difluoromethoxy)phenyl)boronic acid with an overall yield of 21% as a pale yellow oil. **^{1}H NMR** (500 MHz, CDCl3) δ 8.53 – 8.51 (m, 1H), 7.82 – 7.79 (m, 1H), 7.79 – 7.77 (m, 1H), 7.66 – 7.61 (m, 1H), 7.59 – 7.55 (m, 1H), 7.48 – 7.42 (m, 1H), 7.16 – 7.12 (m, 1H), 6.59 (t, *J* = 74.0 Hz, 1H), 2.64 (t, *J* = 7.5 Hz, 2H), 1.75 – 1.64 (m, 2H), 0.98 (t, *J* = 7.3 Hz, 3H). **^{13}C NMR** (125 MHz, CDCl3) δ 153.6, 151.8 (t, *J* = 2.8 Hz), 150.0 – 149.8 (m), 141.5, 136.9, 136.8, 130.0, 123.5, 120.1, 119.5, 117.7, 116.1 (td, *J* = 259.2, 3.1 Hz), 34.7, 24.2, 13.7. **^{19}F NMR** (375 MHz, CDCl3) δ -80.5 (d, *J* = 73.9 Hz). **IR** (thin film): 2962, 1593, 1564, 1470, 1382, 1197, 1045, 792 cm^{-1}. **HRMS** (DART-TOF+): mass [M+H] calc'd for $C_{15}H_{16}F_2ON$ 264.1200; found 264.1197.

2-(2-(difluoromethoxy)phenyl)-5-propylpyridine (nementin-12-24)

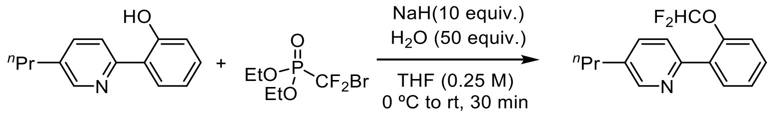

S24 **nementin-12-24**

2-(5-propylpyridin-2-yl)phenol (S24): **S24** was synthesized according to General Procedure A, B, and C using 6-bromonicotinaldehyde, ethyltriphenylphosphonium bromide, and (2-(benzyloxy)phenyl)boronic acid with an overall yield of 15%.

2-(2-(difluoromethoxy)phenyl)-5-propylpyridine (nementin-12-24): **S24** (43 mg, 0.2 mmol, 1 equiv.) was dissolved in THF (0.80 mL, 0.25 M) and cooled to 0 °C. NaH (80 mg, 10 mmol, 60%, 10 equiv.) was added and the mixture was stirred at 0 °C for 30 minutes. H_2O (0.18 mL, 10 mmol, 50 equiv.) was added dropwise and the mixture was stirred at 0 °C for 10 minutes. Diethyl (bromodifluoromethyl)phosphonate (0.07 mL, 0.4 mmol, 2 equiv.) was added and the reaction was allowed to stir from 0 °C to room temperature for 30 minutes. H_2O was added and the aqueous phase was extracted with EtOAc. The combined organics was washed with brine, dried with $MgSO_4$, and concentrated in vacuo. The crude mixture was purified by flash column chromatography, eluting with 5% (v/v) EtOAc:pentanes to give **nementin-12-24** (9 mg, 0.032 mmol, 16%) as a pale yellow oil. **^{1}H NMR** (500 MHz, $CDCl_3$) δ 8.55 – 8.49 (m, 1H), 7.82 – 7.74 (m, 1H), 7.66 – 7.61 (m, 1H), 7.58 – 7.54 (m, 1H), 7.42 – 7.36 (m, 1H), 7.37 – 7.29 (m, 1H), 7.25 – 7.20 (m, 1H), 6.48 (t, *J* = 74.6 Hz, 1H), 2.64 (t, *J* = 7.5 Hz, 2H), 1.75 – 1.66 (m, 2H), 0.99 (t, *J* = 7.3 Hz, 3H). **^{13}C NMR** (125 MHz, $CDCl_3$) δ 152.2, 149.7, 148.6 (t, *J* = 2.8 Hz), 136.5, 136.0, 132.8, 131.5, 129.7, 126.0, 124.3, 120.3, 116.7 (t, *J* = 259.1 Hz), 34.8, 24.2, 13.7. **^{19}F NMR** (375 MHz, $CDCl_3$) δ -80.4 (d, *J* = 74.4 Hz). **IR** (thin film): 2966, 2933, 1600, 1472, 1383, 1213, 1127, 1047, 1027, 758 cm^{-1}. **HRMS** (DART-TOF+): mass [M+H] calc'd for $C_{15}H_{16}F_2ON$ 264.1195; found 264.1195.

2-(4-(difluoromethoxy)phenyl)-5-propylpyrimidine [nementin-12-25]

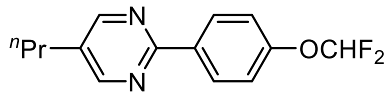

$Pd(OAc)_2$ (3 mg, 0.015 mmol, 5 mol%), PPh_3 (8.0 mg, 0.03 mmol, 10 mol%), K_2CO_3 (124.4 mg, 0.9 mmol, 3 equiv.) and (4-(difluoromethoxy)phenyl)boronic acid (85 mg, 0.45 mmol, 1.5 equiv.) were added to a flamed-dried flask. The mixture was purged with nitrogen for 5 minutes. Dioxane (0.55 mL, 0.55 M) and H_2O (0.14 mL, 2.18 M) were added, followed by 2-chloro-5-propylpyrimidine (0.04 mL, 0.3 mmol, 1 equiv.). The reaction was then refluxed at 100 °C for 16 hours. H_2O was added to the mixture and the aqueous phase was extracted with EtOAc. The combined organics was washed with brine, dried with $MgSO_4$, and concentrated in vacuo. The crude mixture was purified by flash column chromatography, eluting with a 2.5 to 5% (v/v) EtOAc:pentanes gradient to give **nementin-12-25** (69 mg, 0.26 mmol, 86%) as a white solid. **^{1}H NMR** (500 MHz, $CDCl_3$) δ 8.61 (s, 2H), 8.46 – 8.41 (m, 2H), 7.23 – 7.18 (m, 2H), 6.59 (t, *J* = 73.8 Hz, 1H), 2.61 (t, *J* = 7.5 Hz, 2H), 1.75 – 1.65 (m, 2H), 1.00 (t, *J* = 7.4 Hz, 3H). **^{13}C NMR** (125 MHz, $CDCl_3$) δ 161.7, 157.1, 153.0 (t, *J* = 2.7 Hz), 134.8, 132.8, 129.6, 119.0, 115.8 (t, *J* = 259.6 Hz), 32.2, 24.0, 13.6. **^{19}F NMR** (375 MHz, $CDCl_3$) δ -81.0 (d, *J* = 73.2 Hz). **IR** (thin film): 2963, 1588, 1431, 1383, 1220, 1126, 1048, 799 cm^{-1}. **HRMS** (DART-TOF+): mass [M+H] calc'd for $C_{14}H_{15}F_2ON_2$ 265.1147; found 265.1148. **mp** 37-39 °C.

2-(4-(difluoromethoxy)phenyl)-5-isobutylpyridine [nementin-12-26]

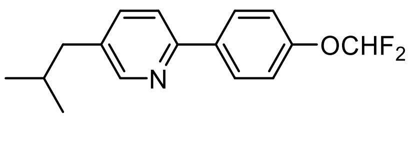

nementin-12-26 was synthesized according to General Procedure A, B, and C using 6-bromonicotinaldehyde,

isopropyltriphenylphosphonium iodide, and (4-(difluoromethoxy)phenyl)boronic acid with an overall yield of 23% as a white solid. ^{1}**H NMR** (500 MHz, $CDCl_3$) δ 8.49 – 8.45 (m, 1H), 8.03 – 7.95 (m, 2H), 7.64 – 7.59 (m, 1H), 7.56 – 7.51 (m, 1H), 7.23 – 7.17 (m, 2H), 6.56 (t, J = 73.9 Hz, 1H), 2.52 (d, J = 7.1 Hz, 2H), 1.95 – 1.85 (m, 1H), 0.95 (d, J = 6.6 Hz, 7H). ^{13}**C NMR** (125 MHz, $CDCl_3$) δ 153.8, 151.6 (t, J = 2.9 Hz), 150.4, 137.3, 136.7, 135.4, 128.2, 119.7, 119.5, 115.9 (t, J = 259.7 Hz), 42.0, 30.0, 22.2. ^{19}**F NMR** (375 MHz, $CDCl_3$) δ -80.8 (d, J = 73.3 Hz). **IR** (thin film): 2959, 1598, 1511, 1476, 1383, 1227, 1125, 1045, 836, 804 cm^{-1}. **HRMS** (DART-TOF+): mass [M+H] calc'd for $C_{16}H_{18}F_2ON$ 278.1351; found 278.1351. **mp** 29-30 °C.

5-butyl-2-(4-(difluoromethoxy)phenyl)pyridine [nementin-12-27]

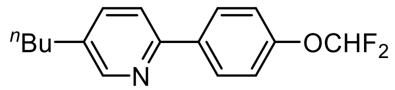

nementin-12-27 was synthesized according to General Procedure A, B, and C using 6-bromonicotinaldehyde, propyltriphenylphosphonium bromide, and (4-(difluoromethoxy)phenyl)boronic acid with an overall yield of 32% as an amorphous solid. ^{1}**H NMR** (500 MHz, $CDCl_3$) δ 8.59 – 8.45 (m, 1H), 8.03 – 7.91 (m, 2H), 7.66 – 7.47 (m, 2H), 7.24 – 7.15 (m, 2H), 6.56 (t, J = 73.9 Hz, 1H), 2.65 (t, J = 7.7 Hz, 2H), 1.70 – 1.56 (m, 3H), 1.46 – 1.32 (m, 2H), 0.95 (t, J = 7.3 Hz, 4H). ^{13}**C NMR** (125 MHz, $CDCl_3$) δ 153.8, 151.6 (t, J = 3.0 Hz), 149.8, 136.7, 136.7, 136.6, 128.2, 119.8, 119.5, 115.9 (t, J = 259.6 Hz), 33.2, 32.4, 22.2, 13.9. ^{19}**F NMR** (375 MHz, $CDCl_3$) δ -80.7 (d, J = 73.9 Hz). **IR** (thin film): 2961, 2932, 1607, 1477, 1383, 1224, 1129, 865, 827 cm^{-1}. **HRMS** (DART-TOF+): mass [M+H] calc'd for $C_{16}H_{18}F_2ON$ 278.1351; found 278.1351.

5-butyl-2-(4-methoxyphenyl)pyridine [nementin-12-28]

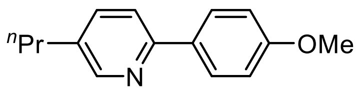

nementin-12-28 was synthesized according to General Procedure A, B, and C using 6-bromonicotinaldehyde, ethyltriphenylphosphonium bromide, and (4-methoxyphenyl)boronic acid with an overall yield of 41% as a white solid. **^{1}H NMR** (500 MHz, $CDCl_3$) δ 8.48 – 8.46 (m, 1H), 7.95 – 7.89 (m, 2H), 7.61 – 7.57 (m, 1H), 7.55 – 7.50 (m, 1H), 7.02 – 6.95 (m, 2H), 3.86 (s, 3H), 2.61 (t, J = 7.5 Hz, 2H), 1.73 – 1.59 (m, 2H), 0.97 (t, J = 7.3 Hz, 3H). **^{13}C NMR** (125 MHz, $CDCl_3$) δ 160.1, 154.7, 149.6, 136.6, 135.4, 132.1, 127.9, 119.3, 114.0, 55.3, 34.7, 24.3, 13.7. **IR** (thin film): 2958, 2869, 1607, 1514, 1476, 1275, 1246, 1174, 1019, 818 cm^{-1}. **HRMS** (DART-TOF+): mass [M+H] calc'd for $C_{15}H_{18}ON$ 228.1383; found 228.1381. **mp** 39-40 °C.

4-(5-butylpyridin-2-yl)phenol [nementin-12-29]

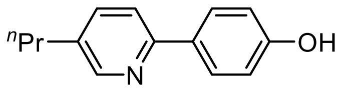

nementin-12-29 was synthesized according to General Procedure A, B, and C using 6-bromonicotinaldehyde, ethyltriphenylphosphonium bromide, and (4-(benzyloxy)phenyl)boronic acid with an overall yield of 31% as a white solid. **^{1}H NMR** (500 MHz, $CDCl_3$) δ 8.45 – 8.39 (m, 1H), 7.73 – 7.64 (m, 2H), 7.60 – 7.53 (m, 2H), 6.78 – 6.72 (m, 2H), 2.59 (t, J = 7.6 Hz, 2H), 1.71 – 1.60 (m, 2H), 0.95 (t, J = 7.4 Hz, 3H). **^{13}C NMR** (125 MHz, $CDCl_3$) δ 158.1 (d, J = 13.0 Hz), 155.3 – 155.3 (m), 148.6 (d, J = 3.9 Hz), 137.8 – 137.3 (m), 135.7, 130.1 (d, J = 12.8 Hz), 128.4, 120.5 (d, J = 5.3 Hz), 115.9 (d, J = 1.5 Hz), 34.6, 24.2, 13.7. **IR** (thin

film): 3851, 2967, 1603, 1479, 1388, 1275, 1244, 1172, 824 cm^{-1}. **HRMS** (DART-TOF+): mass [M+H] calc'd for $C_{14}H_{16}ON$ 214.1226; found 214.1230. **mp** 133-134 °C.

2-(4-(2,2-difluoroethoxy)phenyl)-5-propylpyridine [nementin-12-30]

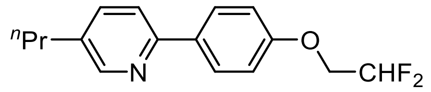

nementin-12-29 (21 mg, 0.1 mmol) was dissolved in DMF (0.33 ml, 0.3 M) and cooled to 0 °C. NaH (6 mg, 0.15 mmol, 60%, 1.5 equiv.) was added and the mixture was stirred at 0 °C for 30 minutes. 2-bromo-1,1-difluoroethane (0.02 mL, 0.15 mmol, 1.5 equiv.) was added dropwise and the reaction mixture was stirred from 0 °C to room temperature overnight. H_2O was added and the aqueous phase was extracted with EtOAc. The combined organics was washed with brine, dried with $MgSO_4$, and concentrated in vacuo. The crude mixture was purified by flash column chromatography, eluting with 5 to 10% (v/v) EtOAc:pentanes to give **nementin-12-30** (18 mg, 0.063 mmol, 63%) as a white solid. **^{1}H NMR** (500 MHz, $CDCl_3$) δ 8.50 – 8.44 (m, 1H), 7.98 – 7.89 (m, 2H), 7.59 (dd, *J* = 8.1, 0.9 Hz, 1H), 7.56 – 7.48 (m, 1H), 7.06 – 6.96 (m, 2H), 6.11 (tt, *J* = 55.2, 4.1 Hz, 1H), 4.24 (td, *J* = 13.0, 4.1 Hz, 2H), 2.61 (t, *J* = 7.4 Hz, 2H), 1.71 – 1.64 (m, 2H), 0.97 (t, *J* = 7.3 Hz, 3H). **^{13}C NMR** (125 MHz, $CDCl_3$) δ 158.2, 154.3, 149.7, 136.7, 135.8, 133.4, 128.1, 119.5, 114.7, 113.6 (t, *J* = 241.1 Hz), 67.3 (t, *J* = 29.6 Hz), 34.7, 24.3, 13.7. **^{19}F NMR** (375 MHz, $CDCl_3$) δ -125.2 (td, *J* = 55.6, 12.9 Hz). **IR** (thin film): 2958, 2931, 2872, 1606, 1514, 1475, 1274, 1240, 1135, 1065, 914, 821 cm^{-1}. **HRMS** (DART-TOF+): mass [M+H] calc'd for $C_{16}H_{18}F_2ON$ 278.1351; found 278.1345. **mp** 67-68 °C.

^{13}C NMR (125 MHz, CDCl$_3$)

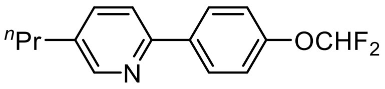

nementin-12

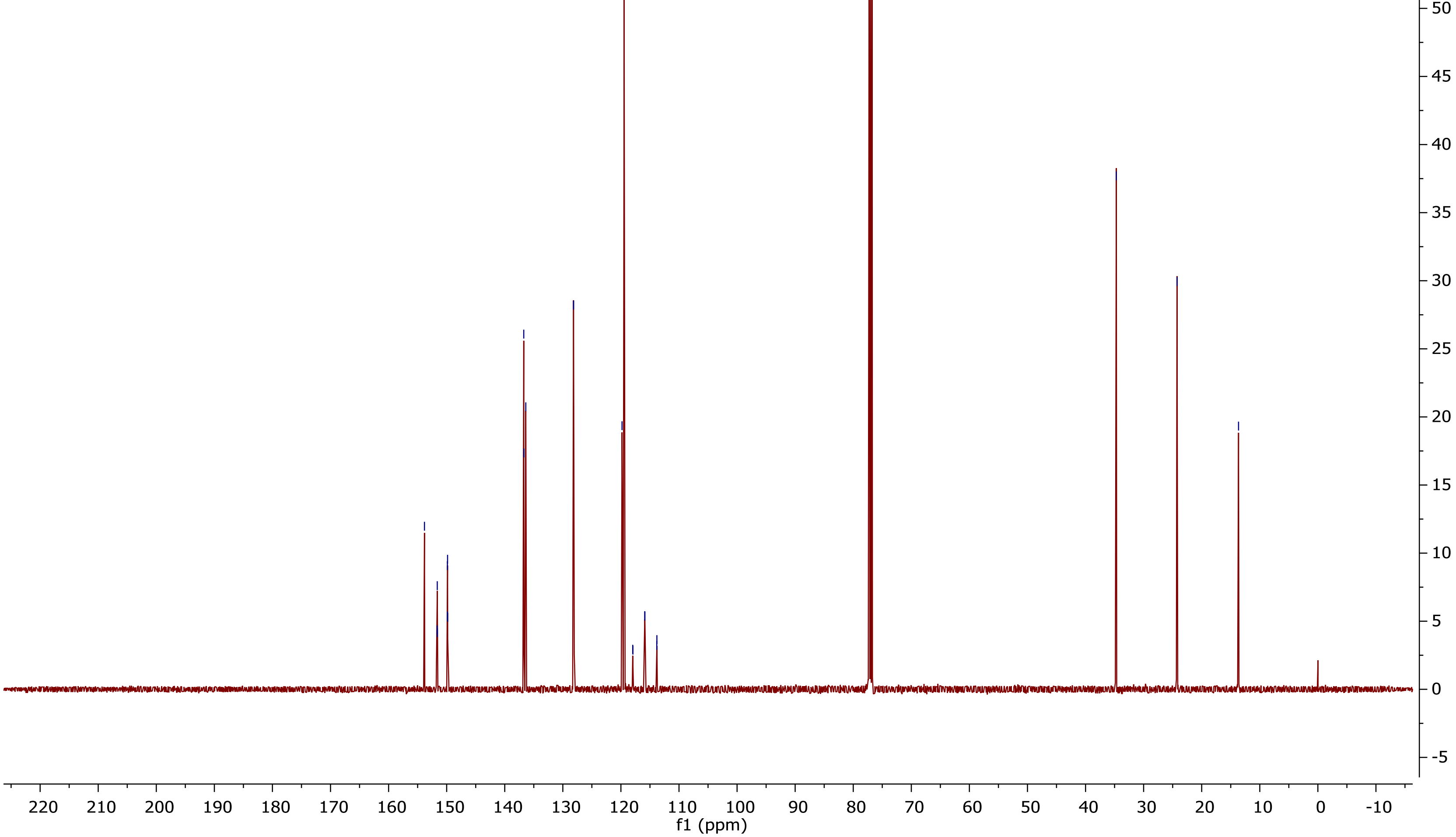

^{19}F NMR (375 MHz, $CDCl_3$)

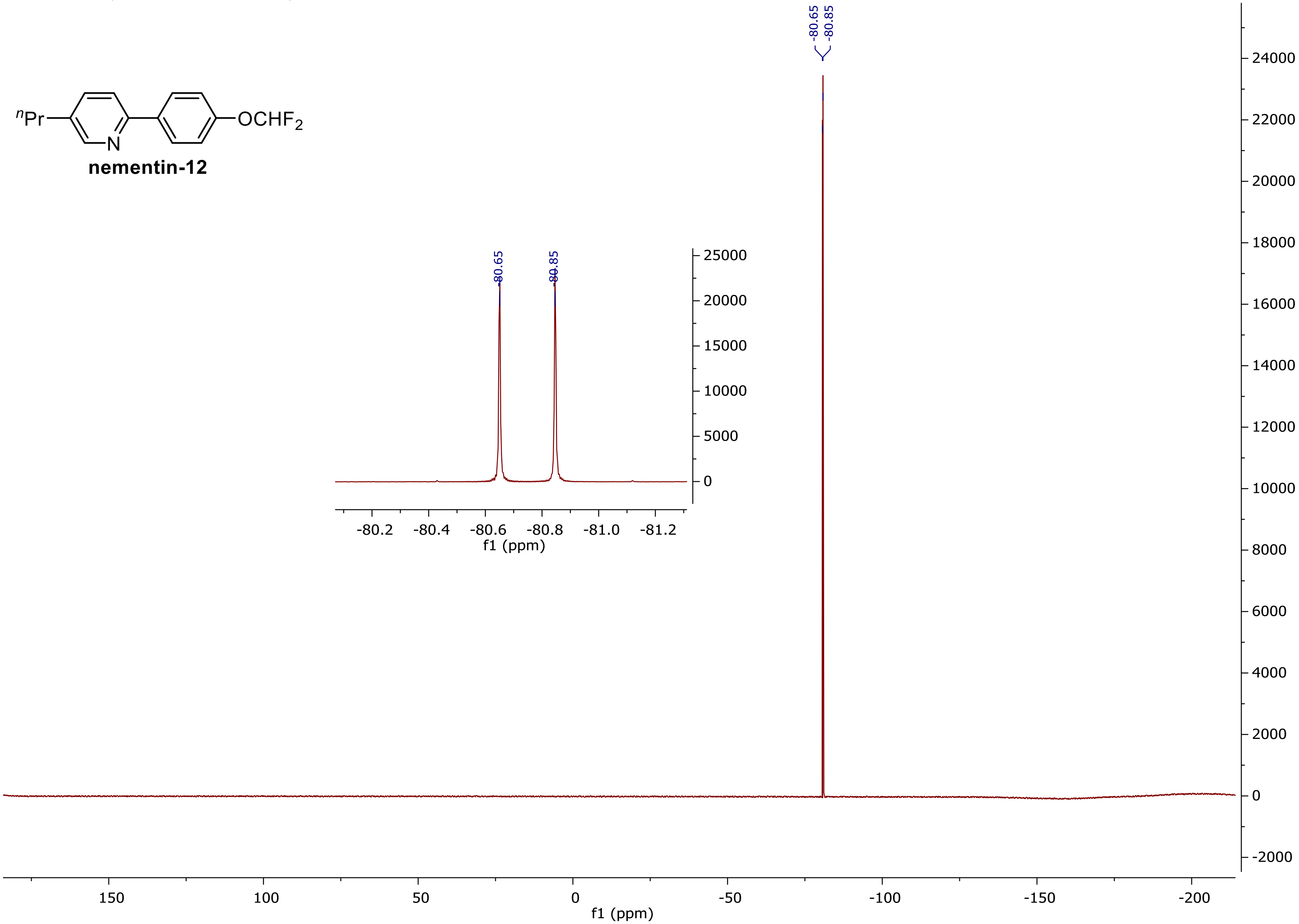

nementin-12

^1H NMR (500 MHz, $CDCl_3$) (nementin-12-5)

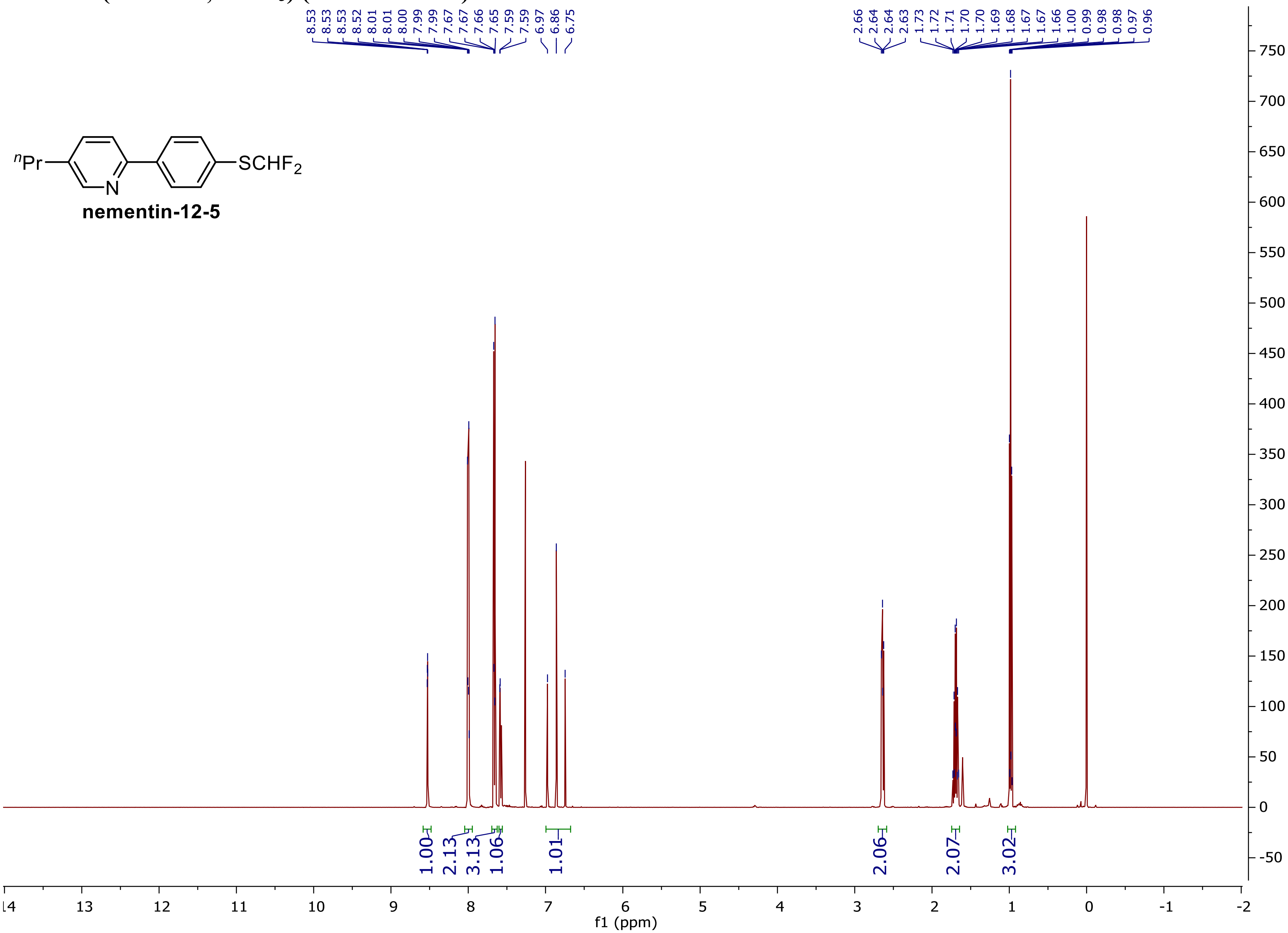

^{13}C NMR (125 MHz, $CDCl_3$)

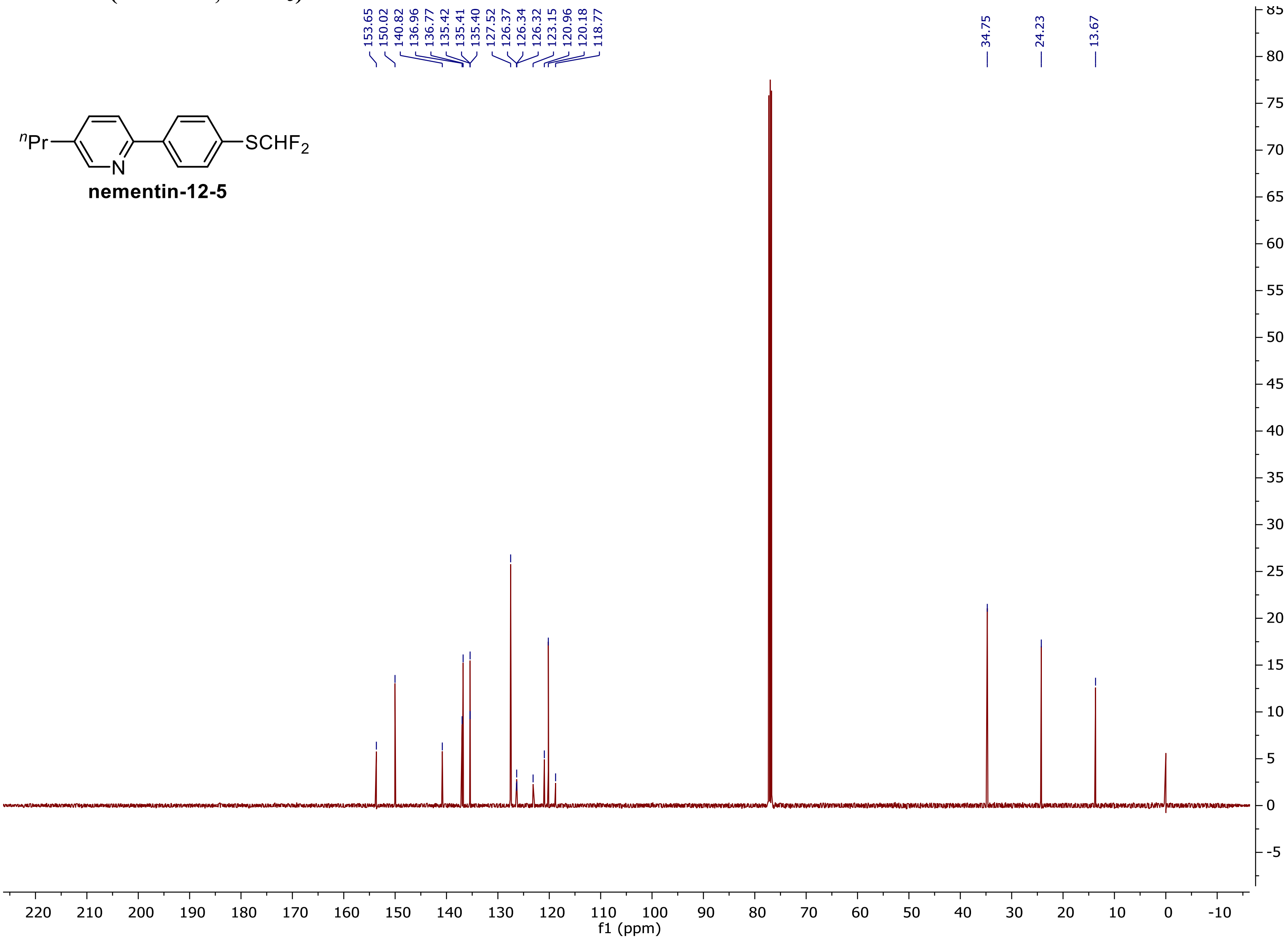

^{19}F NMR (375 MHz, $CDCl_3$)

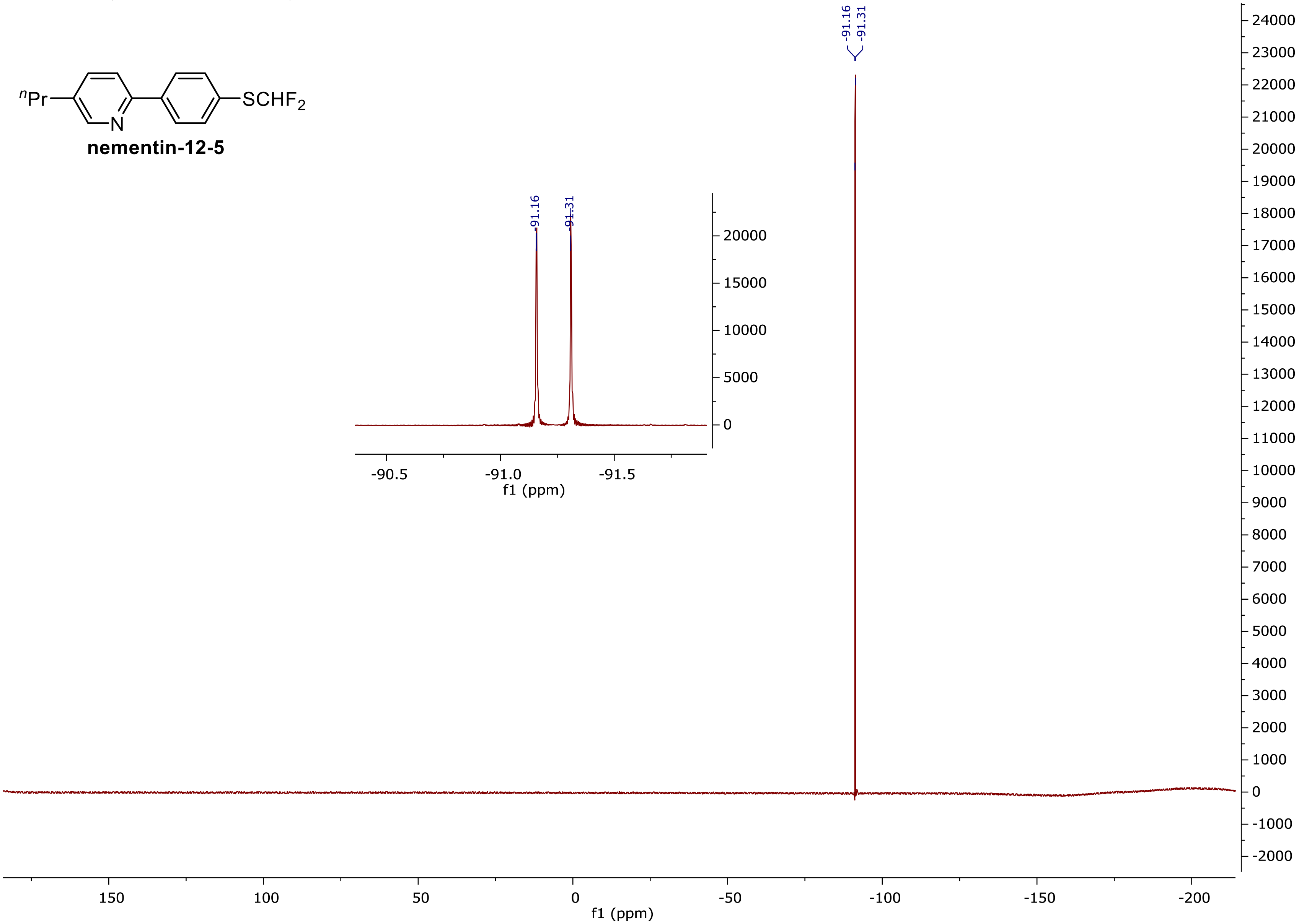

^{1}H NMR (500 MHz, $CDCl_3$) (nementin-12-6)

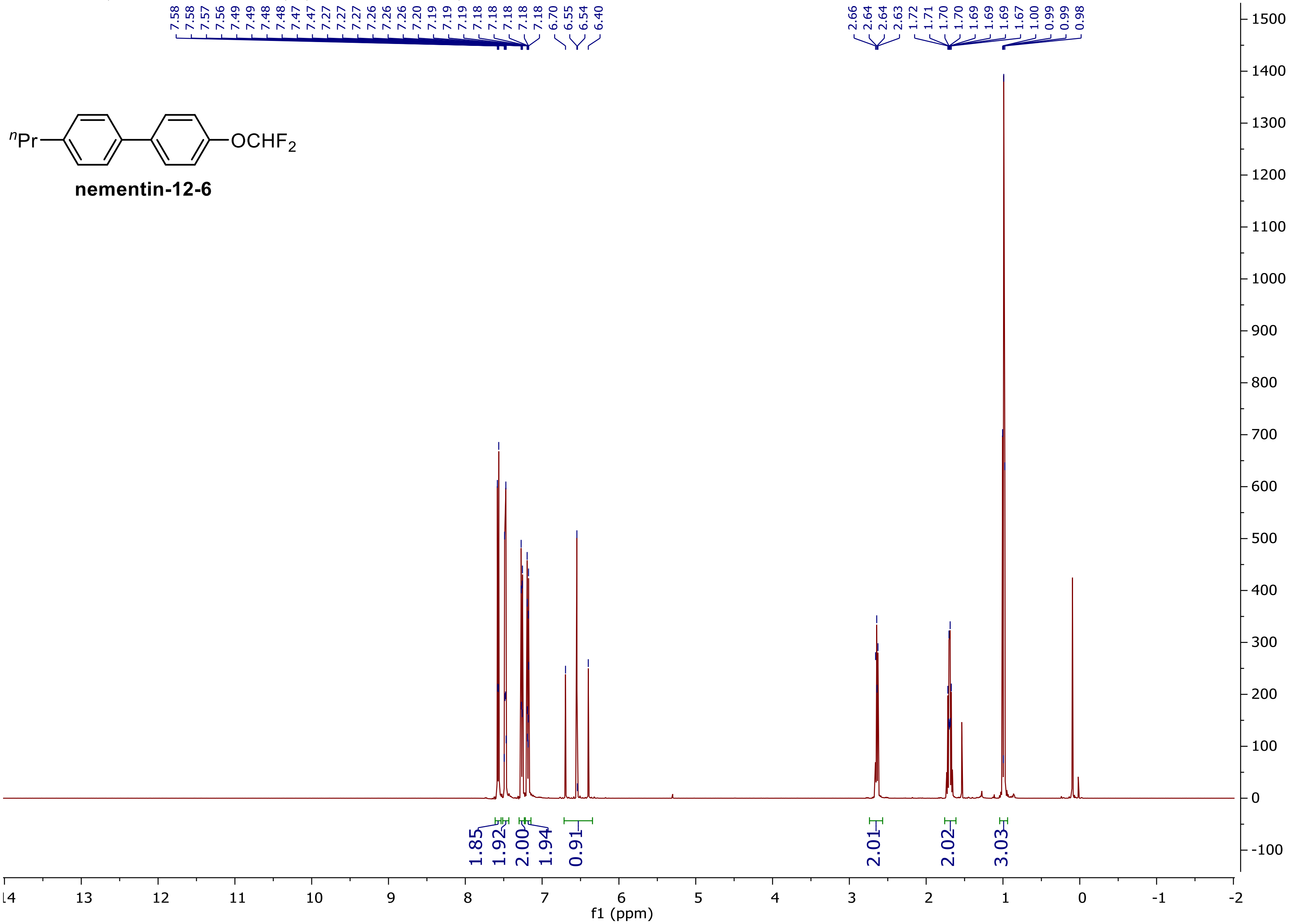

^{13}C NMR (125 MHz, $CDCl_3$)

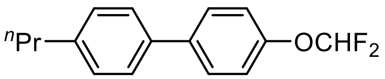

nementin-12-6

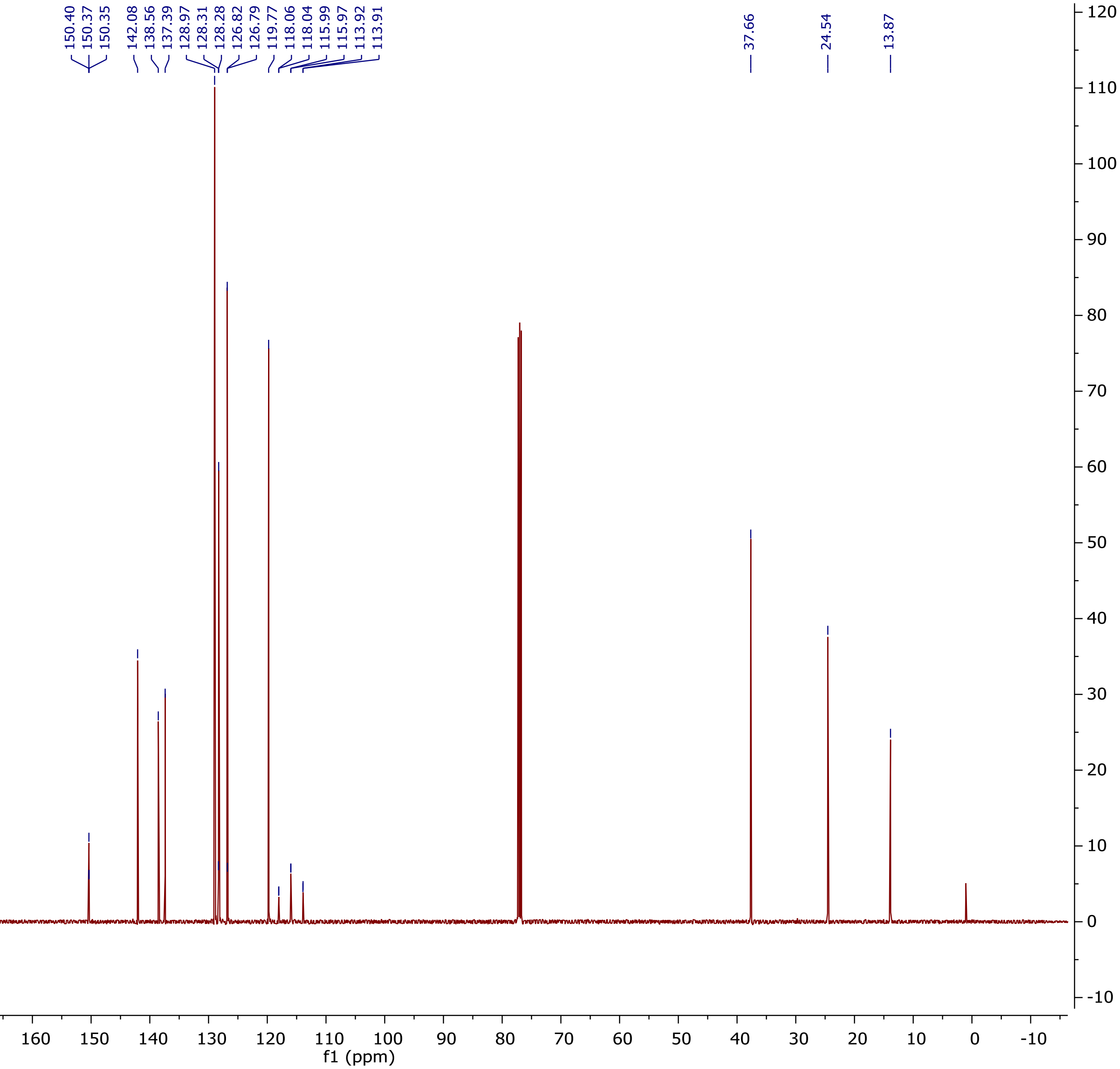

^{19}F NMR (375 MHz, $CDCl_3$)

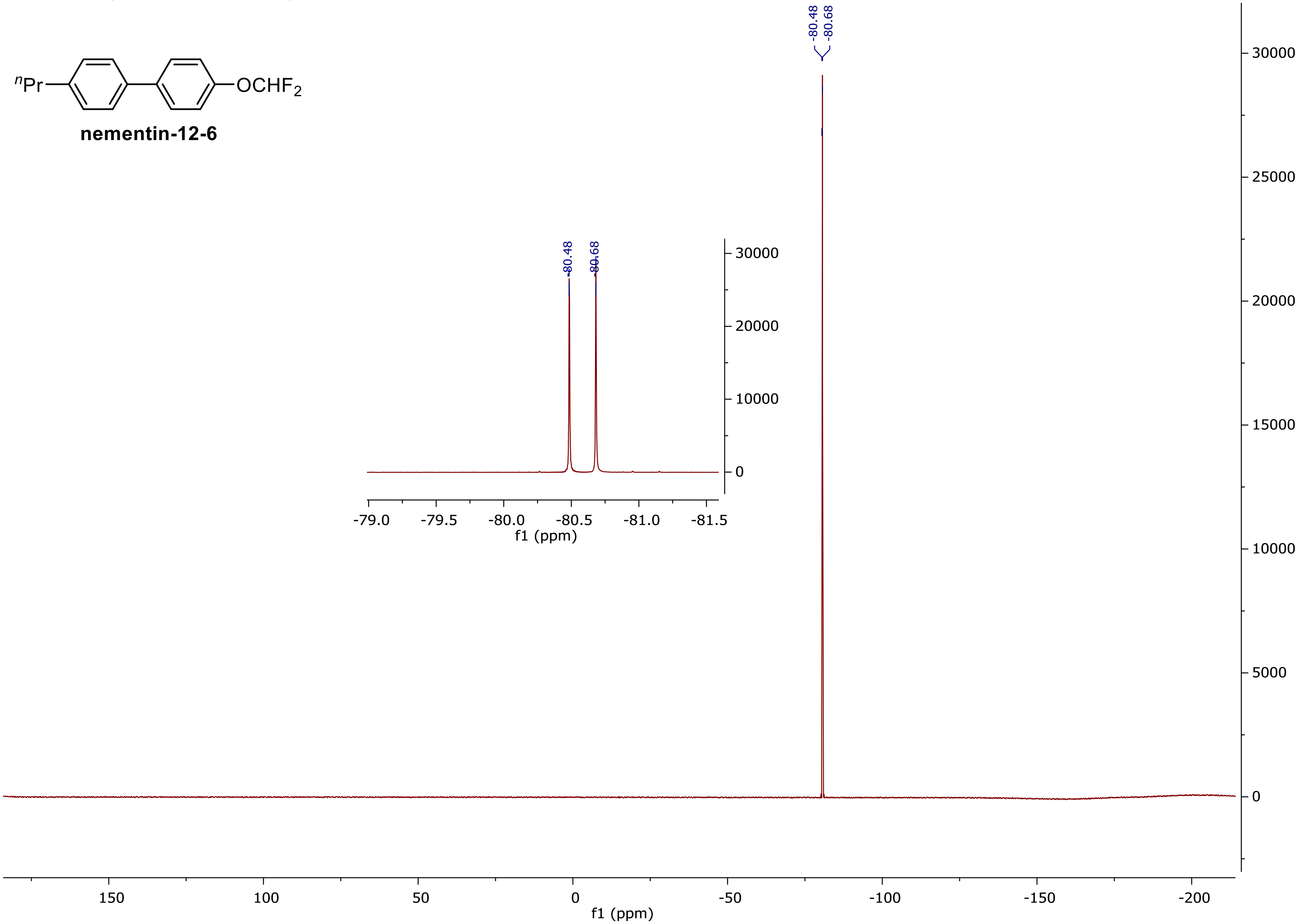

nementin-12-6

^{1}H NMR (500 MHz, $CDCl_3$) (nementin-12-8)

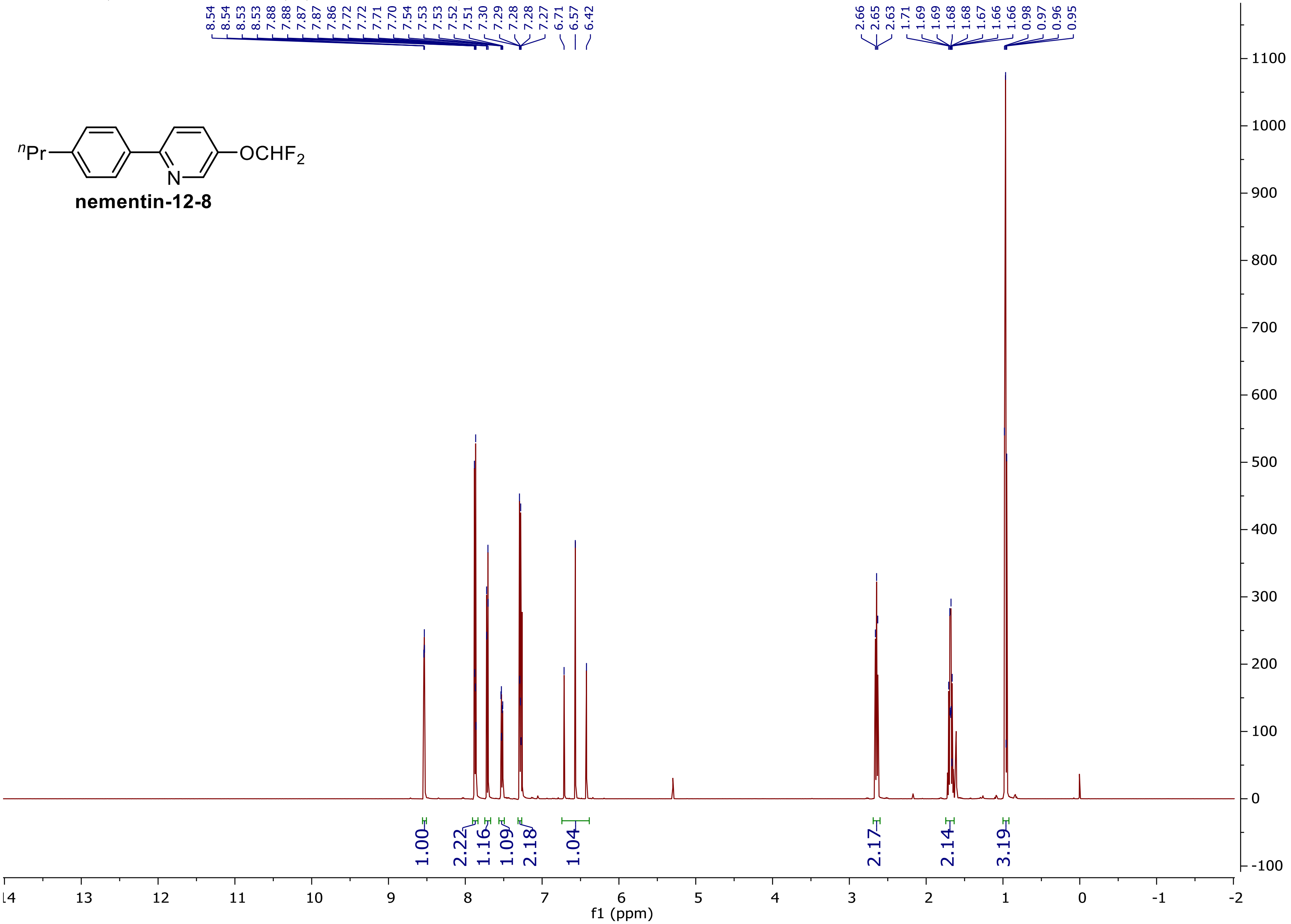

^{13}C NMR (125 MHz, CDCl$_3$)

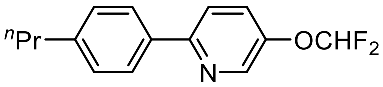

nementin-12-8

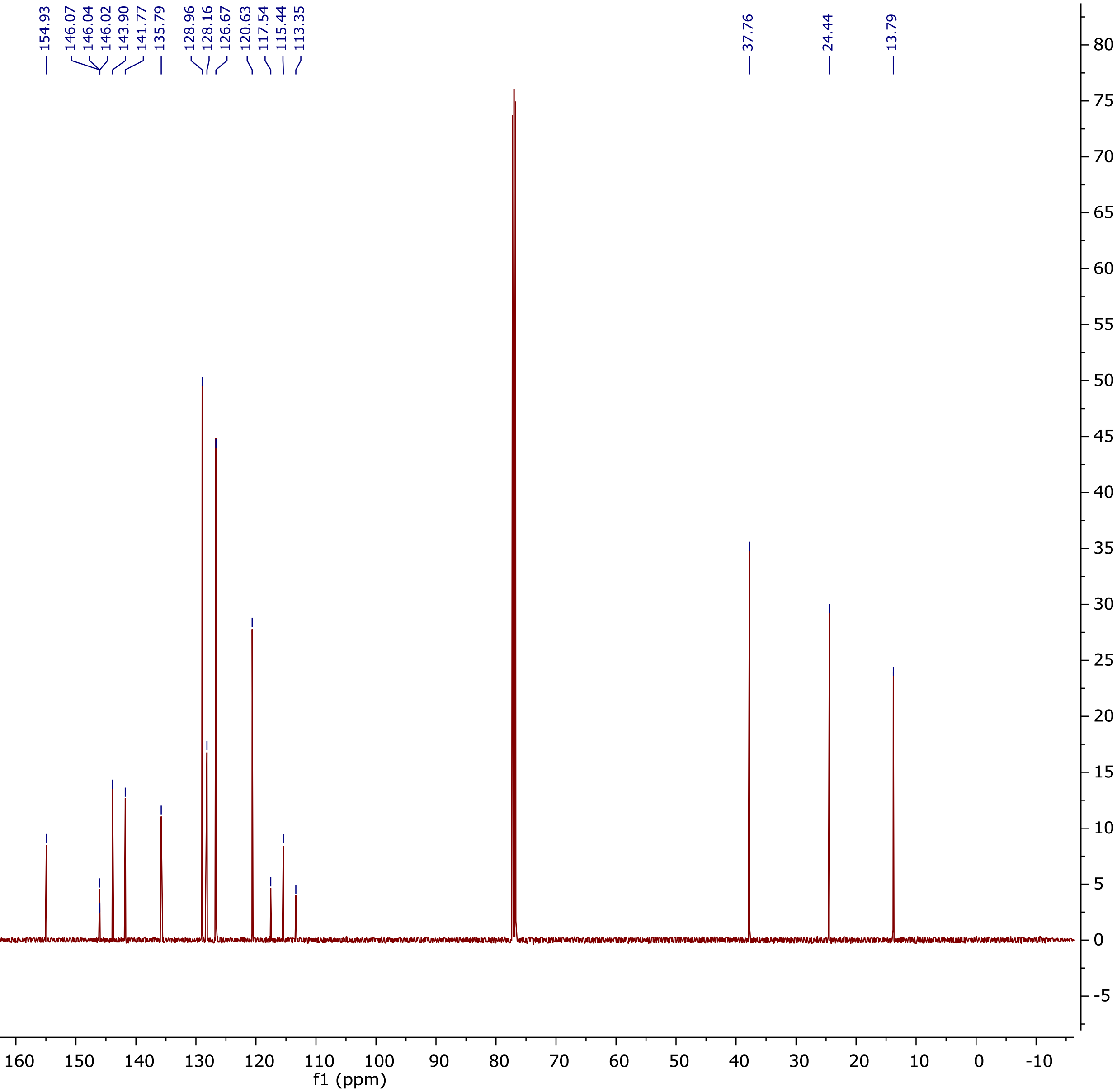

^{19}F NMR (375 MHz, $CDCl_3$)

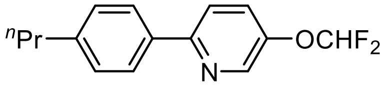

nementin-12-8

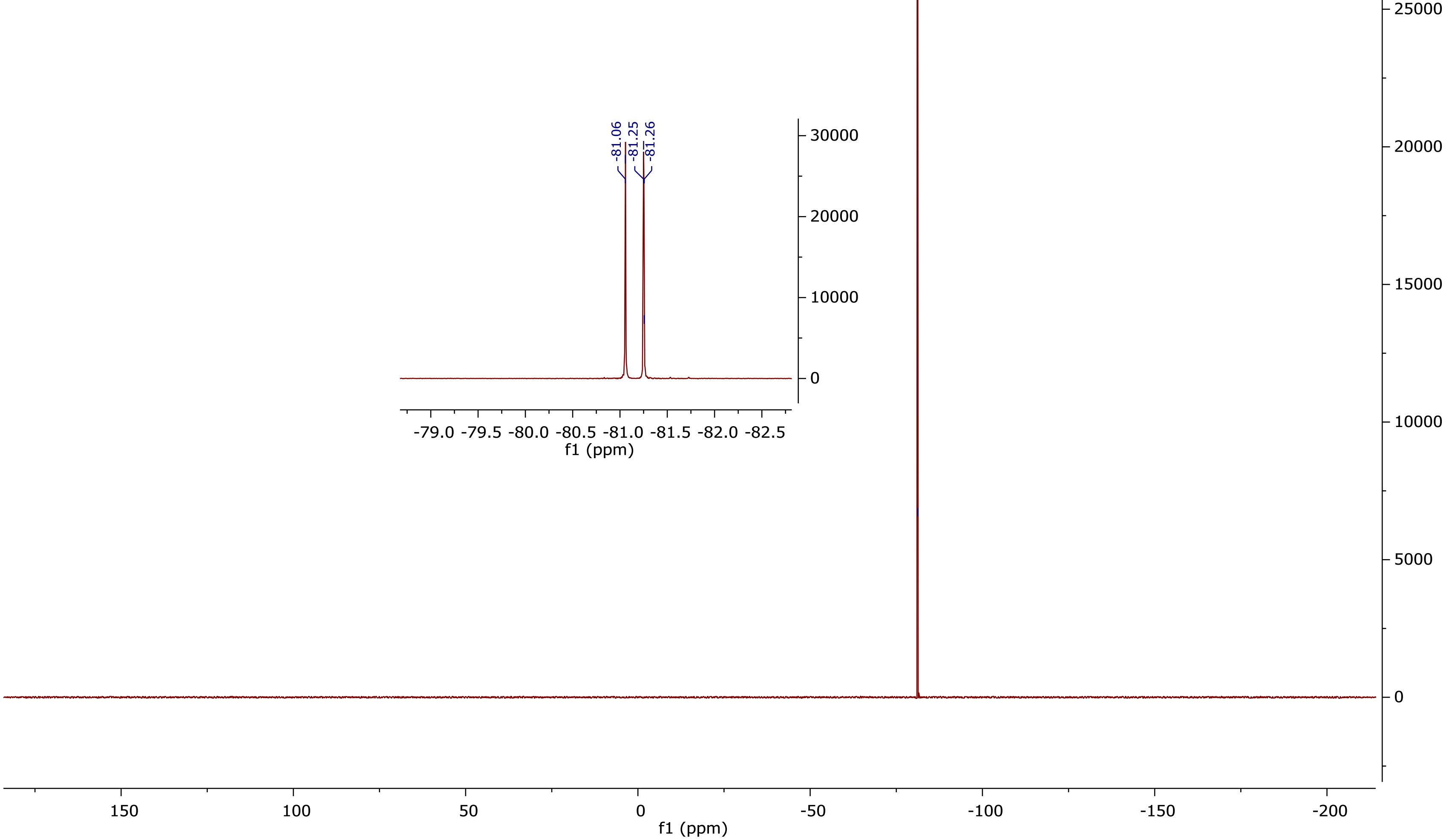

^{1}H NMR (500 MHz, CDCl$_3$) (nementin-12-9)

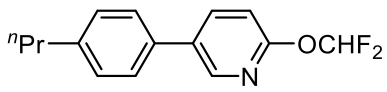

nementin-12-9

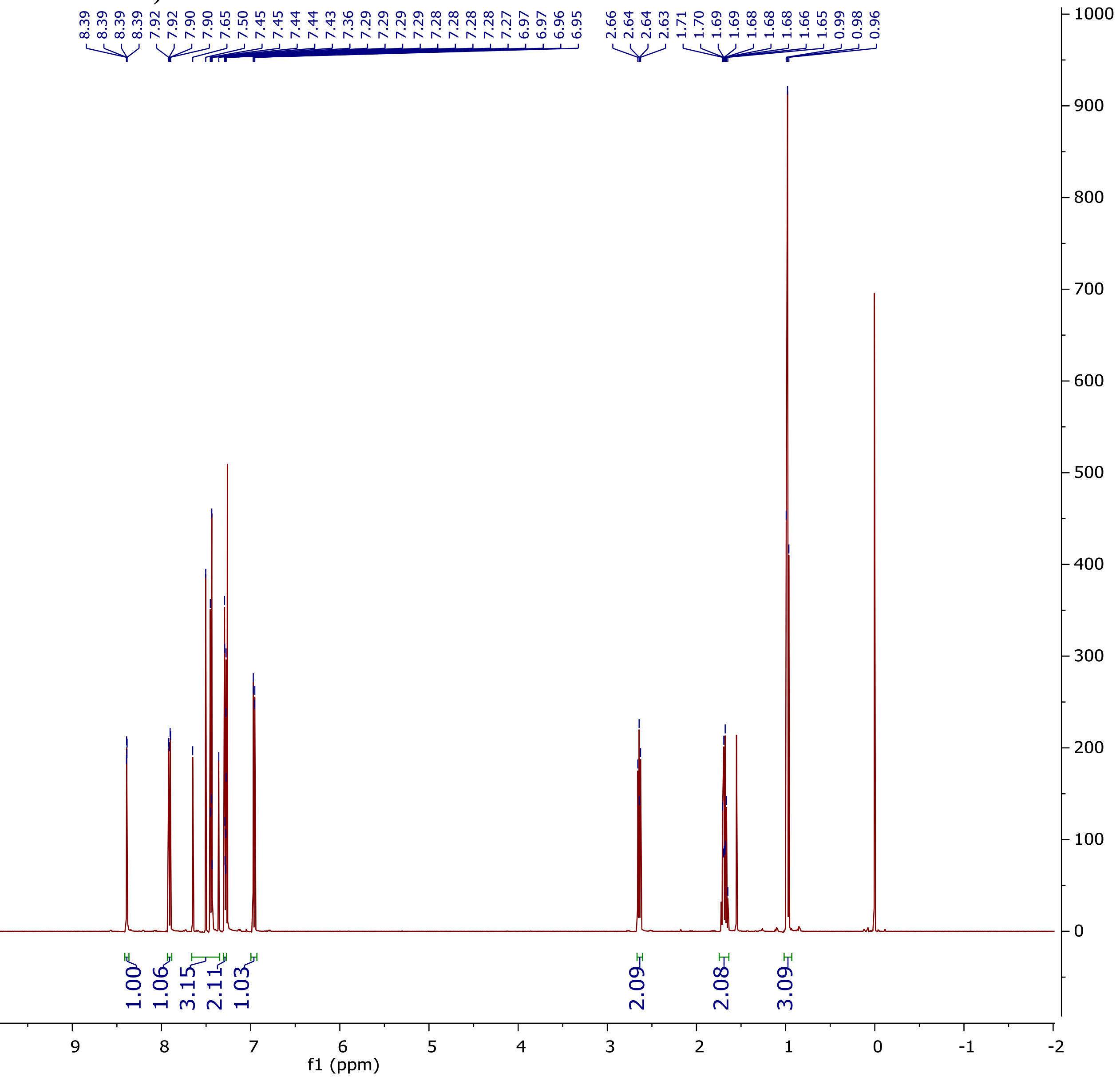

^{13}C NMR (125 MHz, $CDCl_3$)

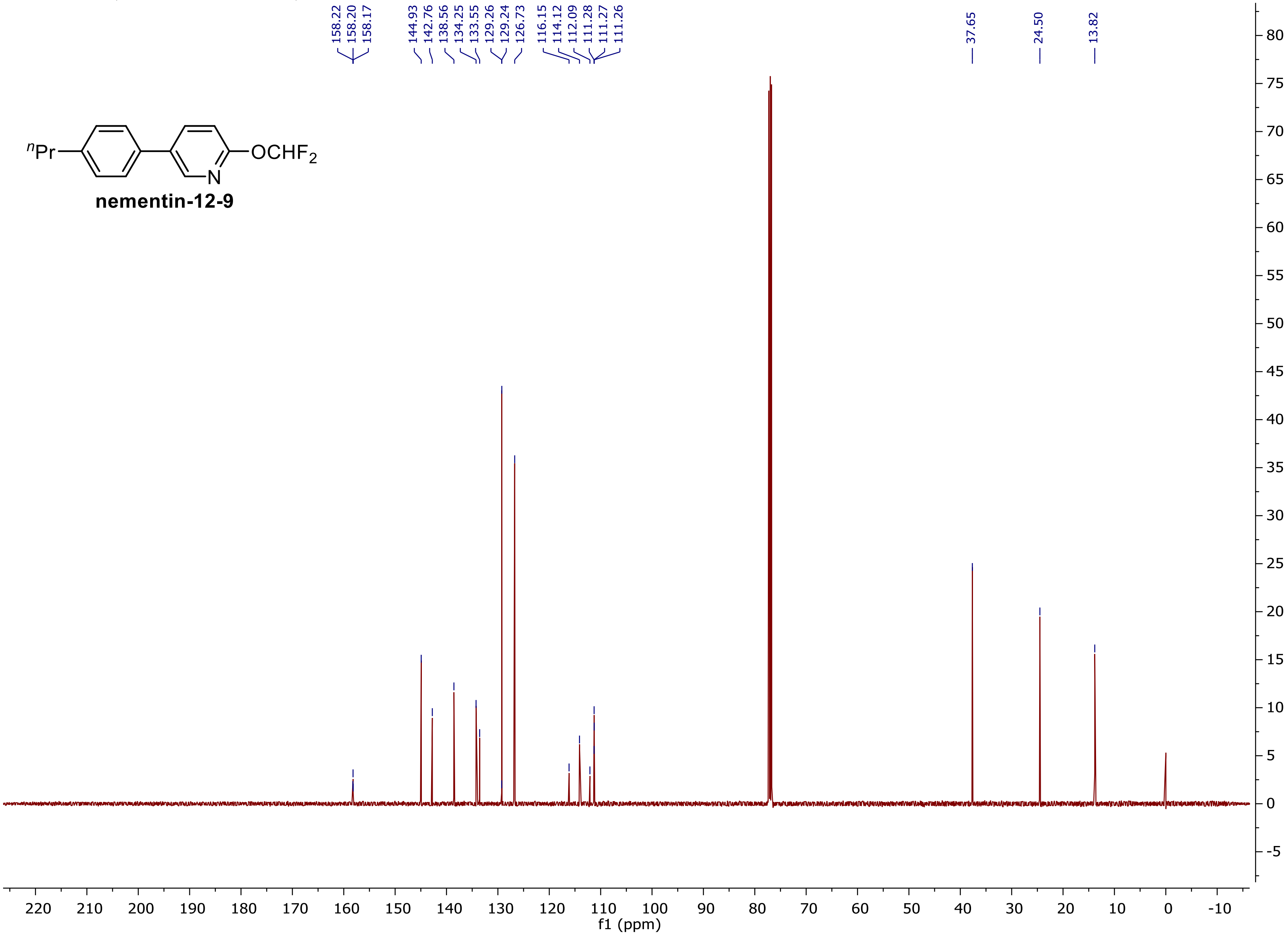

^{19}F NMR (375 MHz, CDCl$_3$)

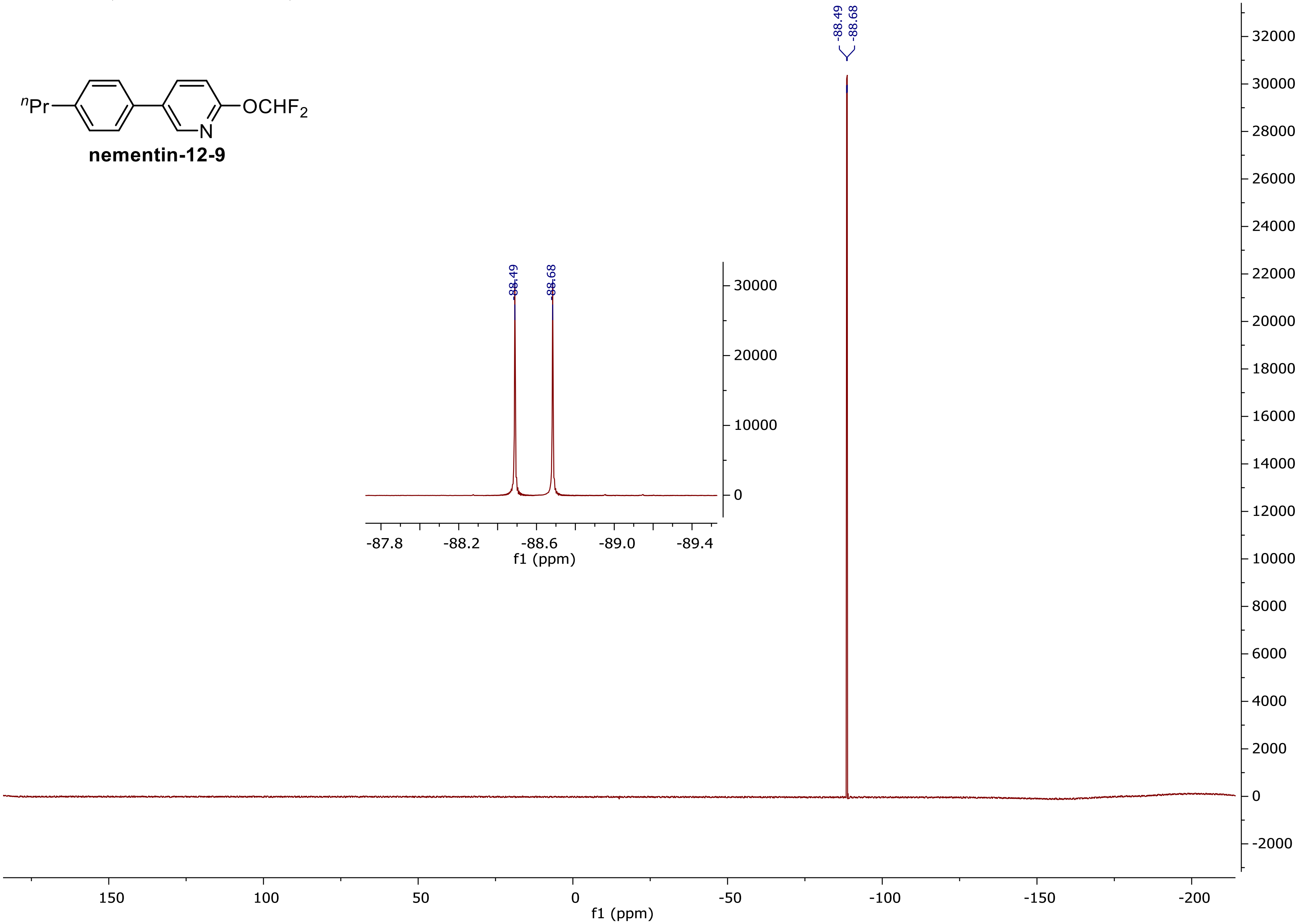

^{1}H NMR (500 MHz, $CDCl_3$) (nementin-12-10)

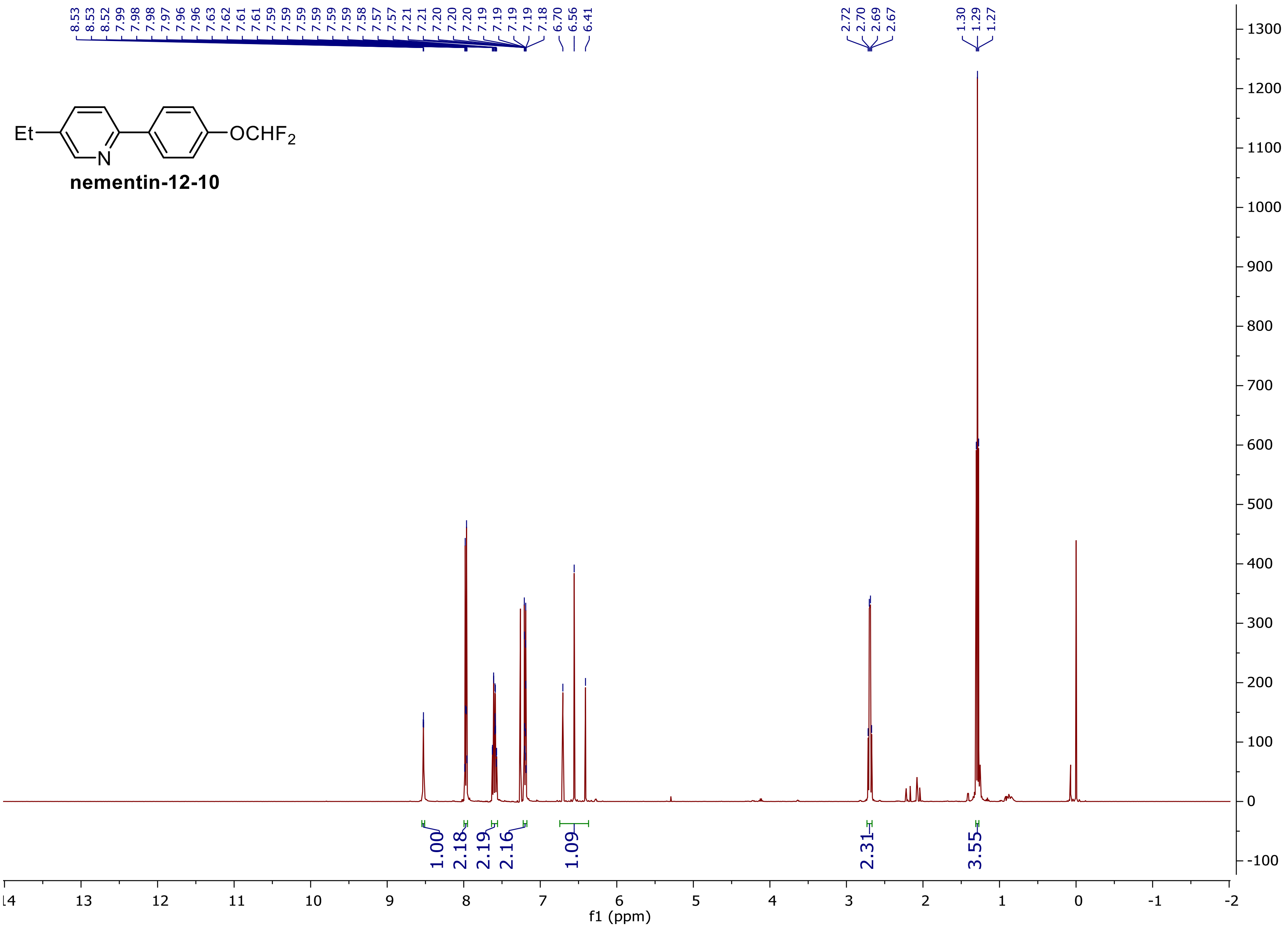

^{13}C NMR (125 MHz, $CDCl_3$)

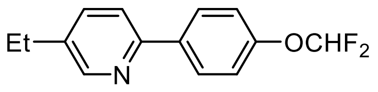

nementin-12-10

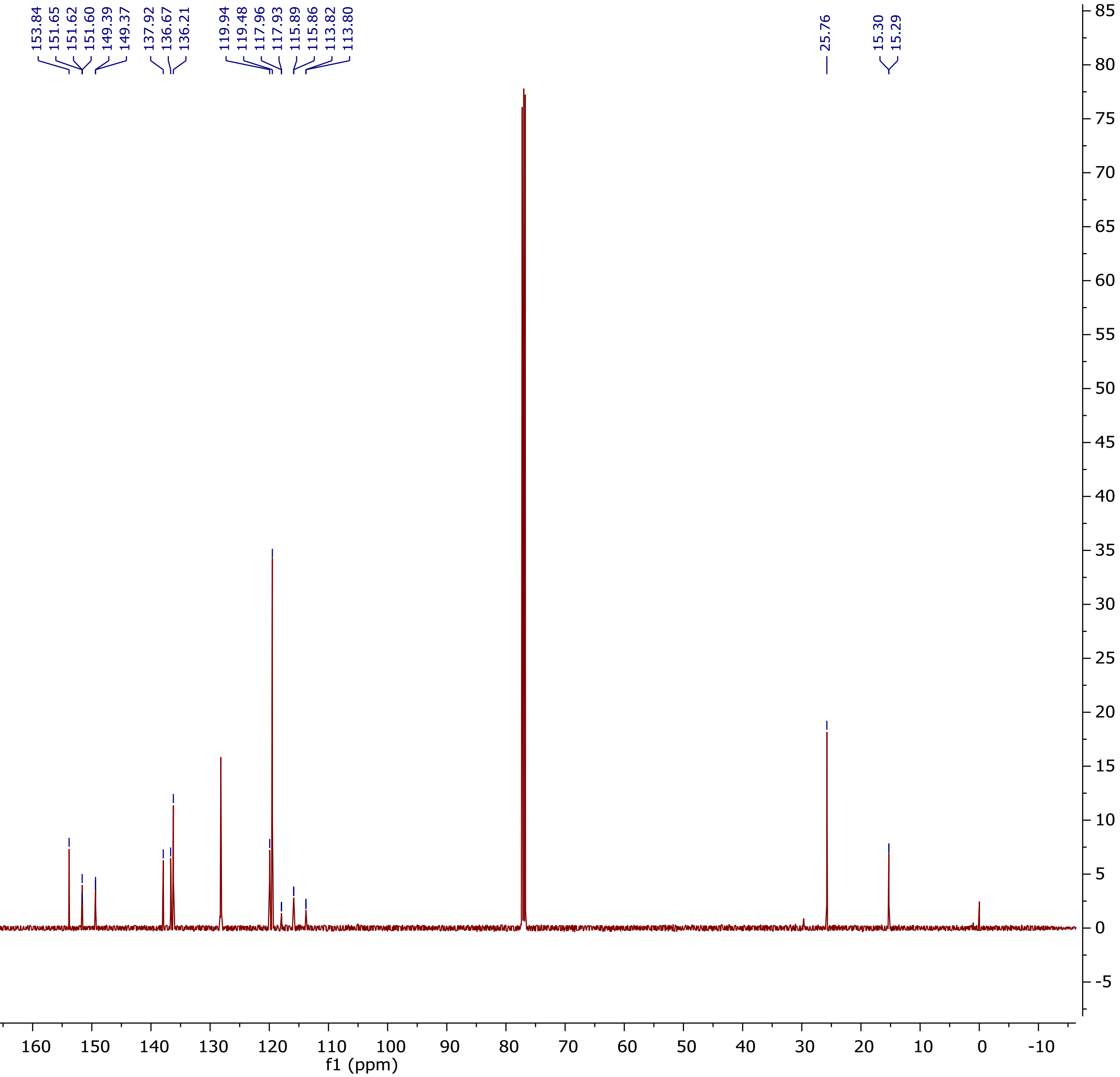

^{19}F NMR (375 MHz, $CDCl_3$)

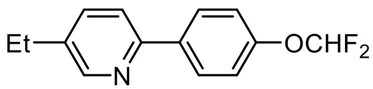

nementin-12-10

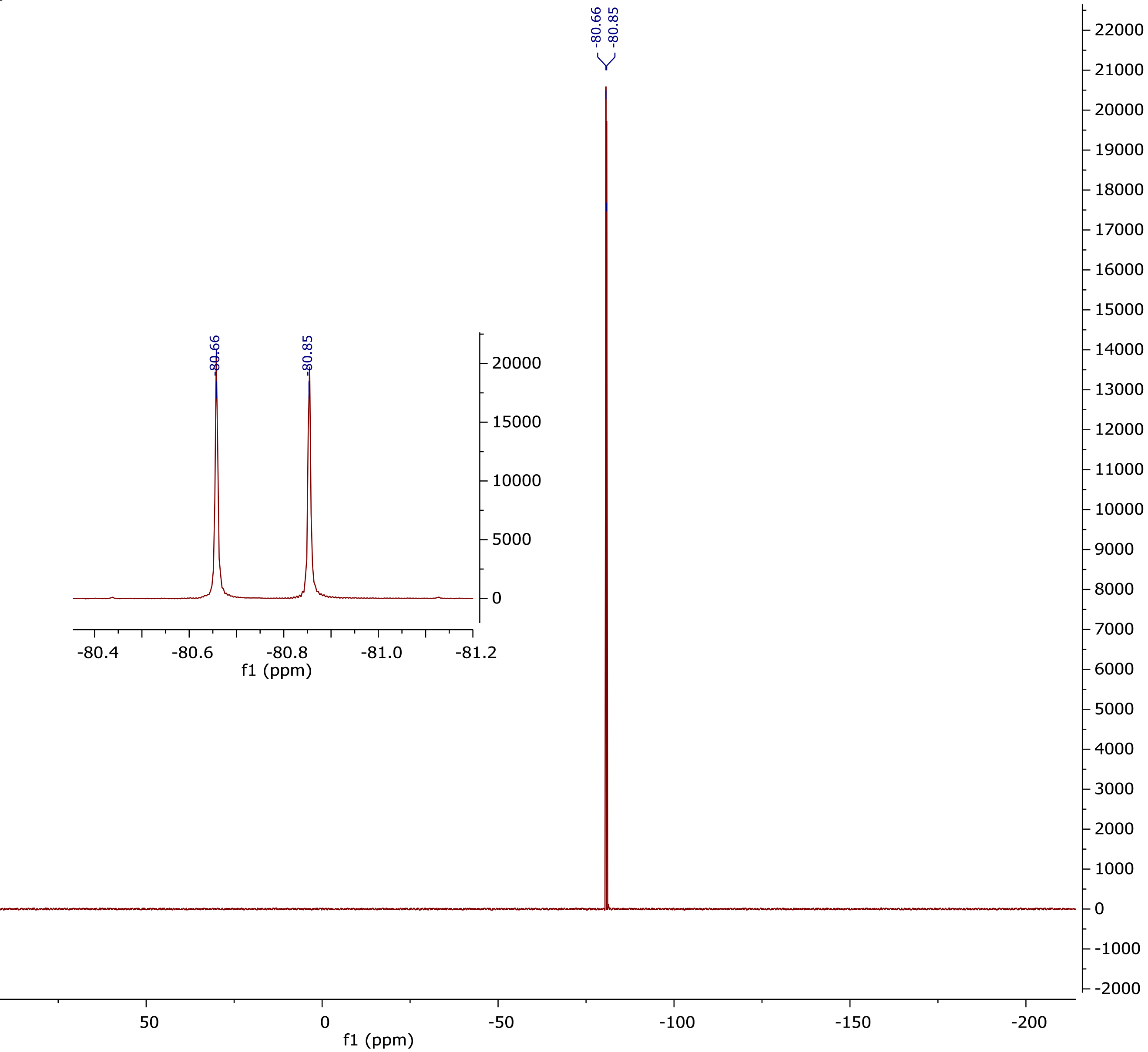

^{1}H NMR (500 MHz, $CDCl_3$) (nementin-12-11)

nementin-12-11

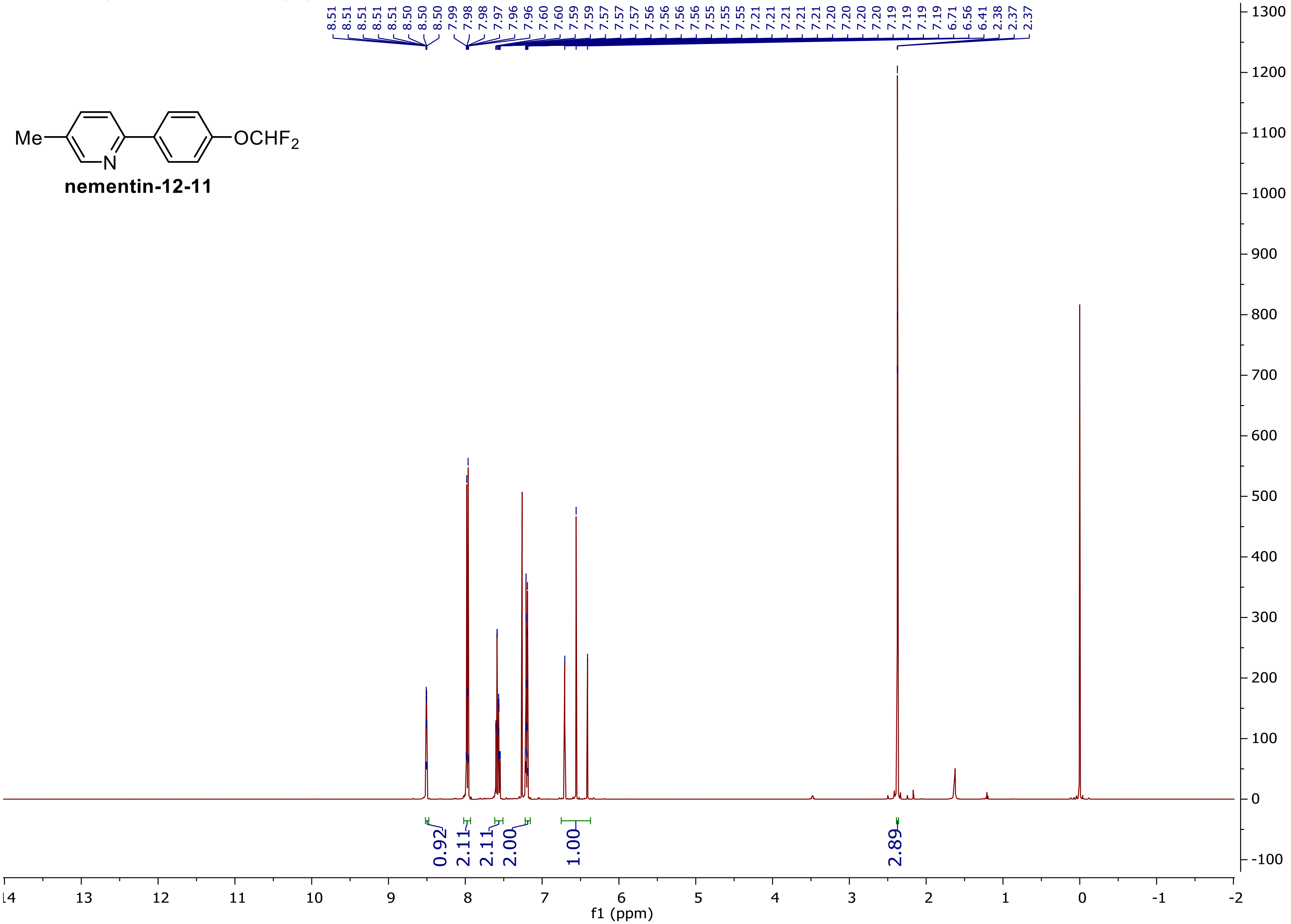

^{13}C NMR (125 MHz, CDCl$_3$)

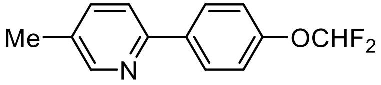

nementin-12-11

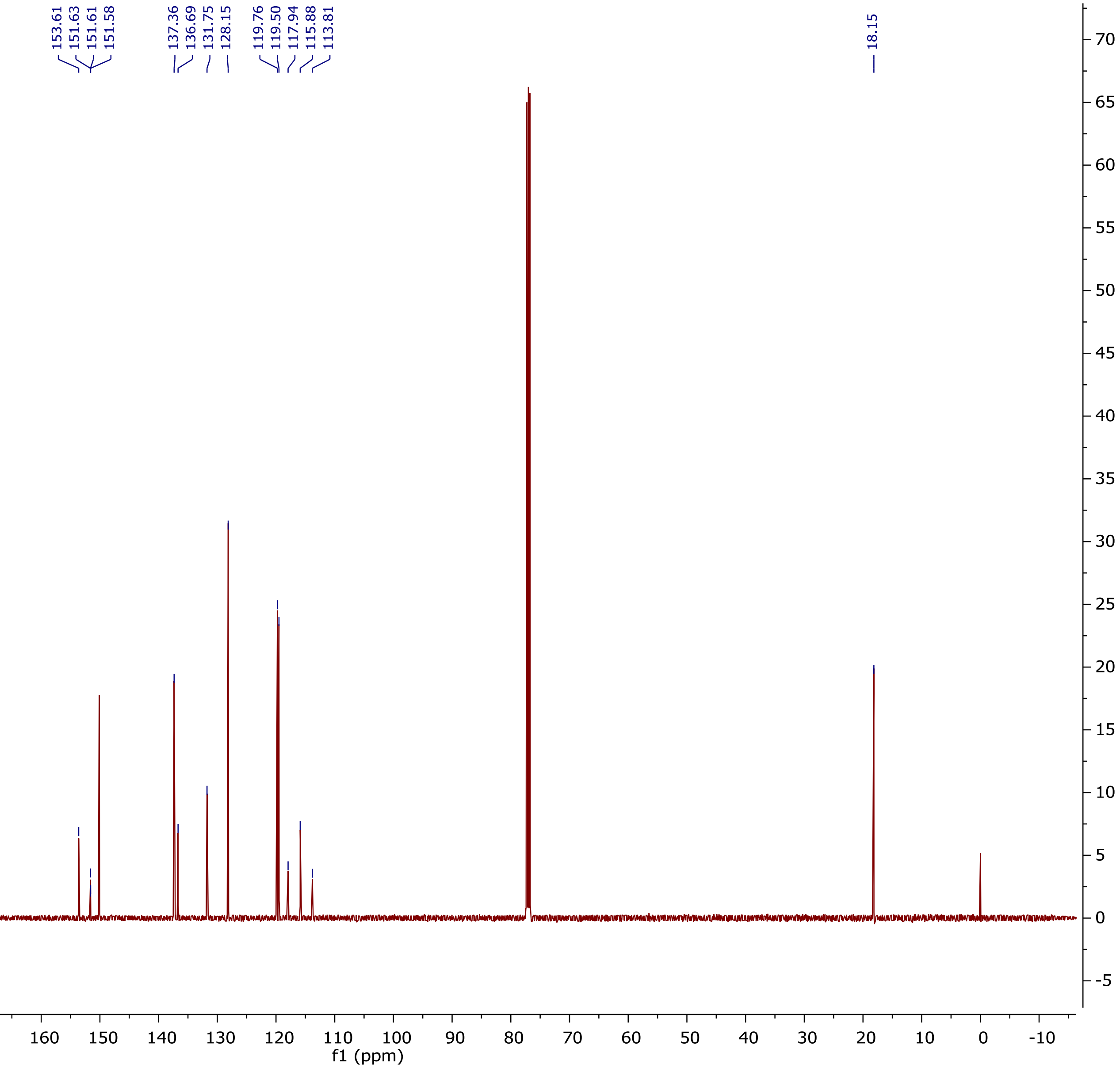

^{19}F NMR (375 MHz, $CDCl_3$)

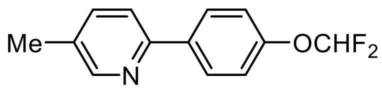

nementin-12-11

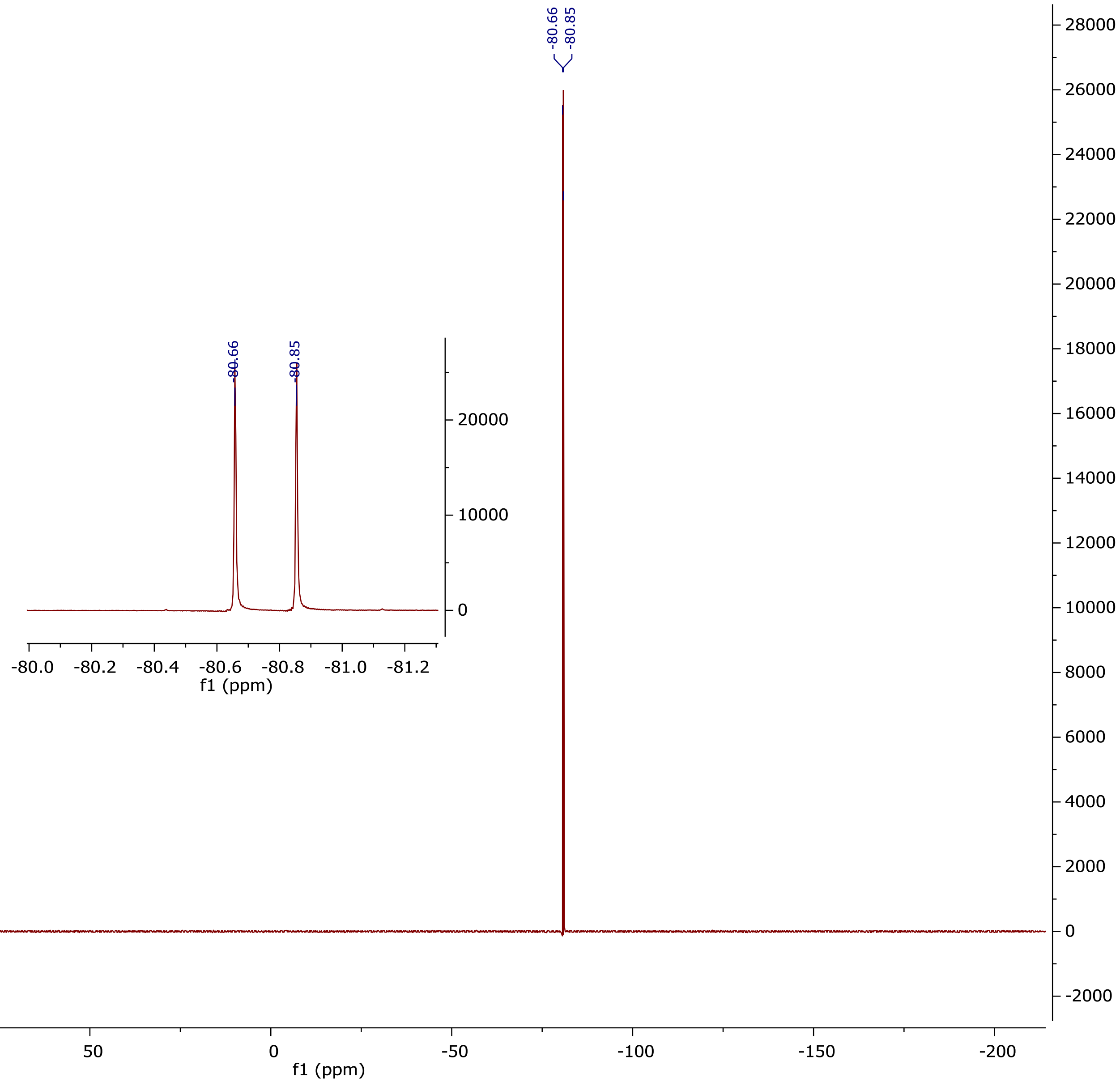

^{1}H NMR (500 MHz, $CDCl_3$) (nementin-12-12)

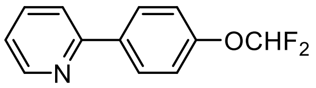

nementin-12-12

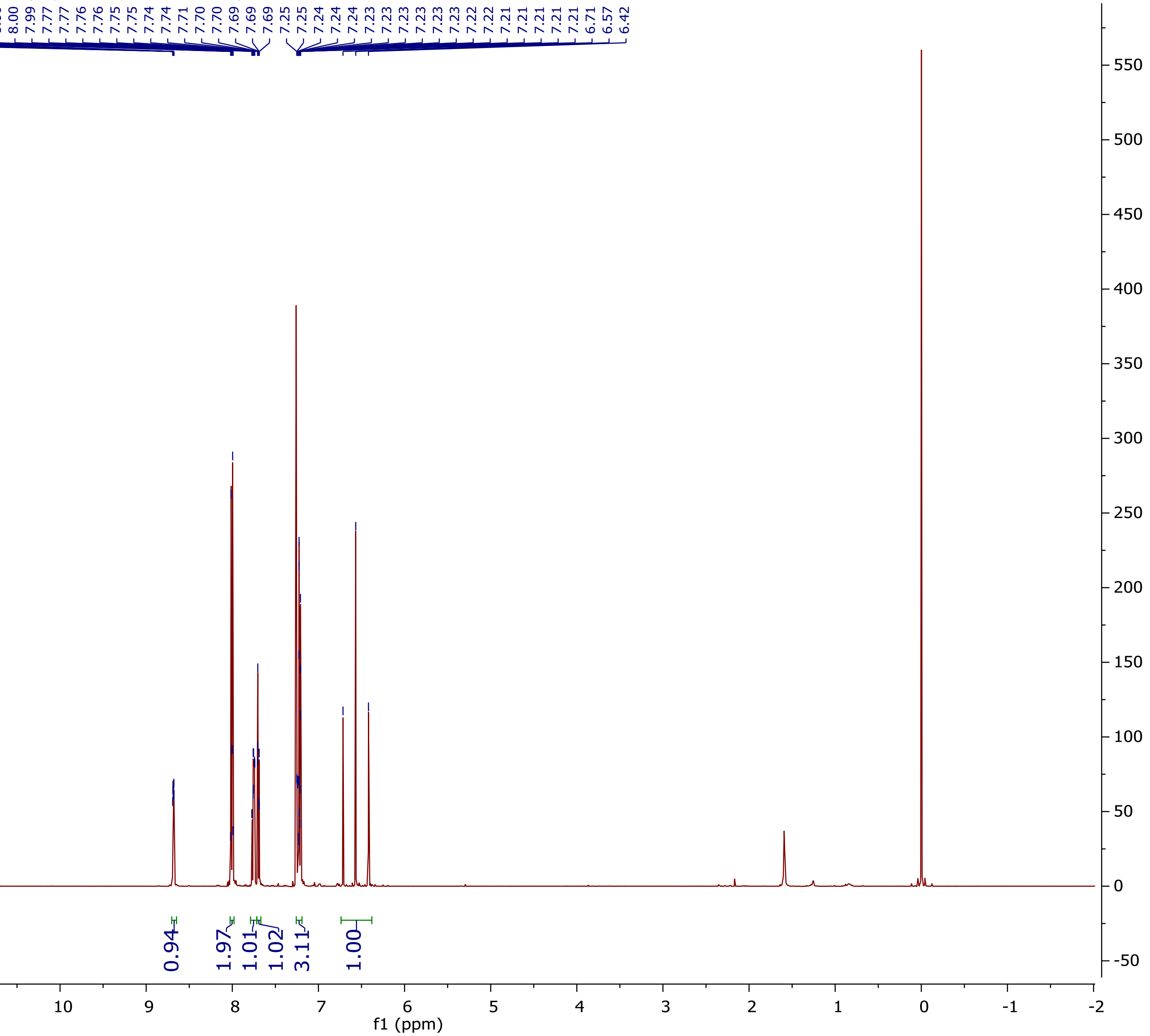

^{13}C NMR (125 MHz, $CDCl_3$)

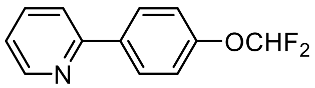

nementin-12-12

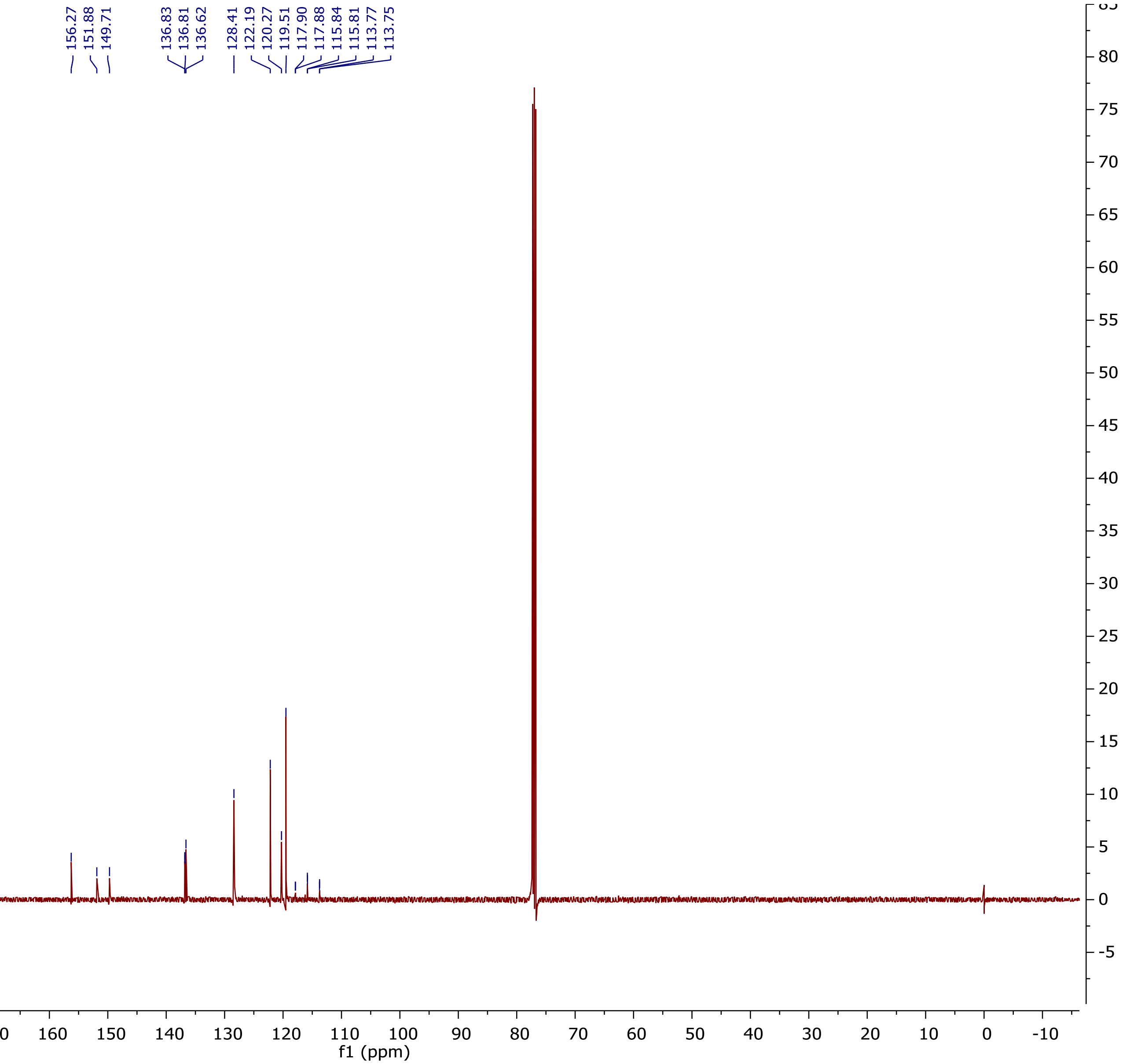

^{19}F NMR (375 MHz, CDCl$_3$)

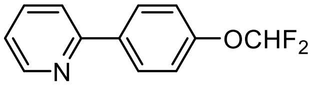

nementin-12-12

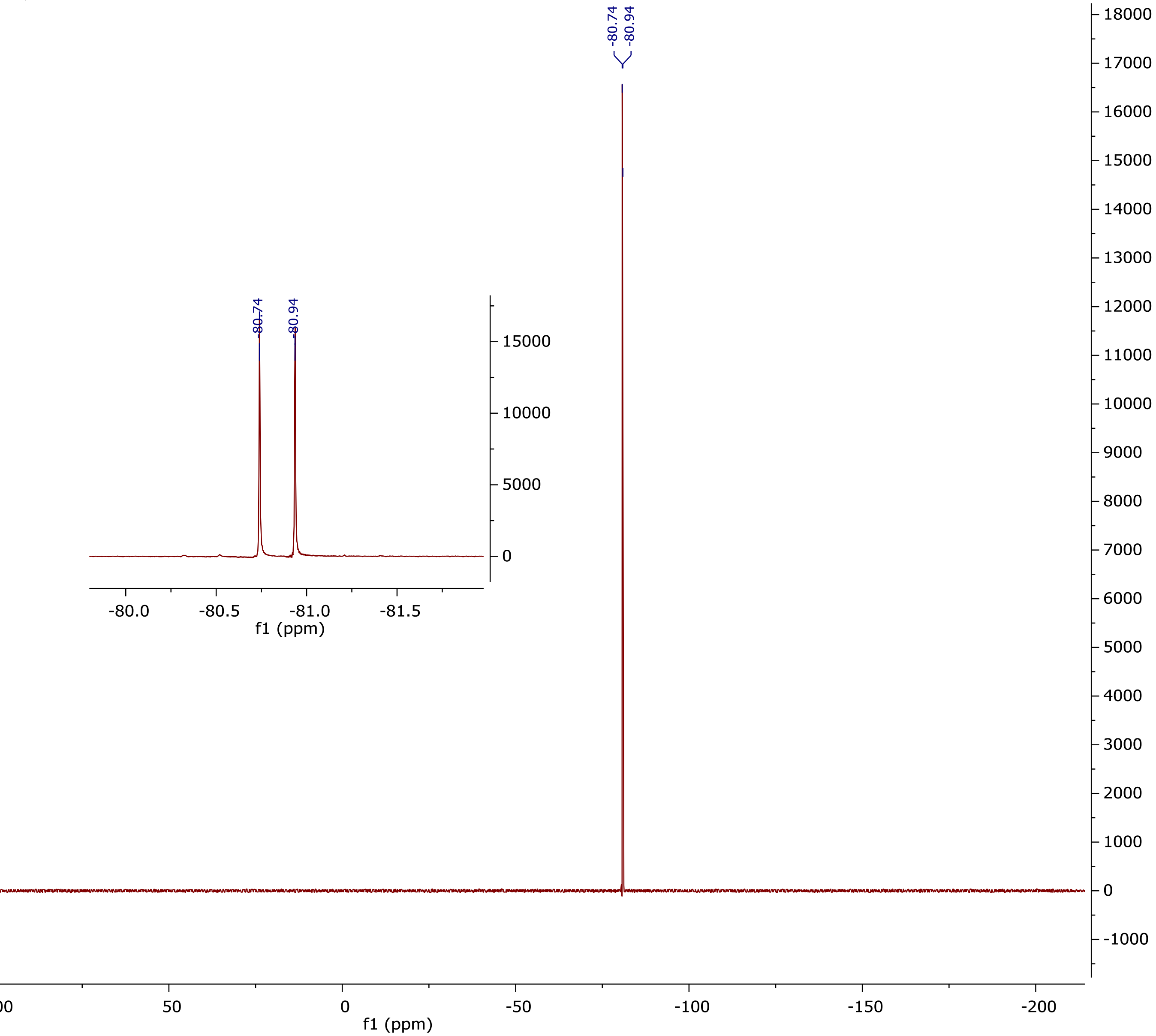

^{1}H NMR (500 MHz, $CDCl_3$) (nementin-12-14)

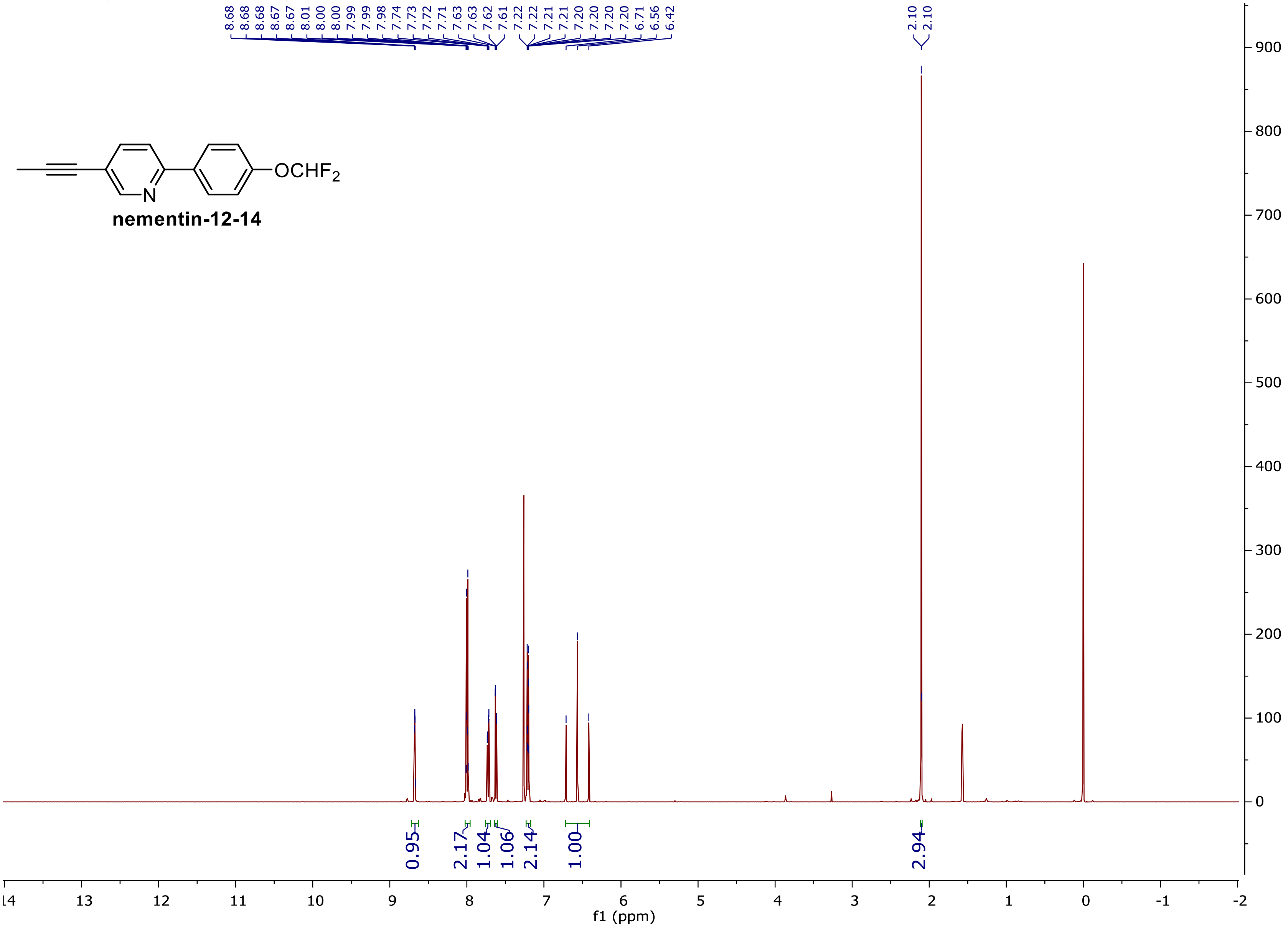

^{13}C NMR (125 MHz, $CDCl_3$)

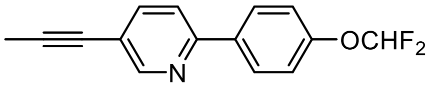

nementin-12-14

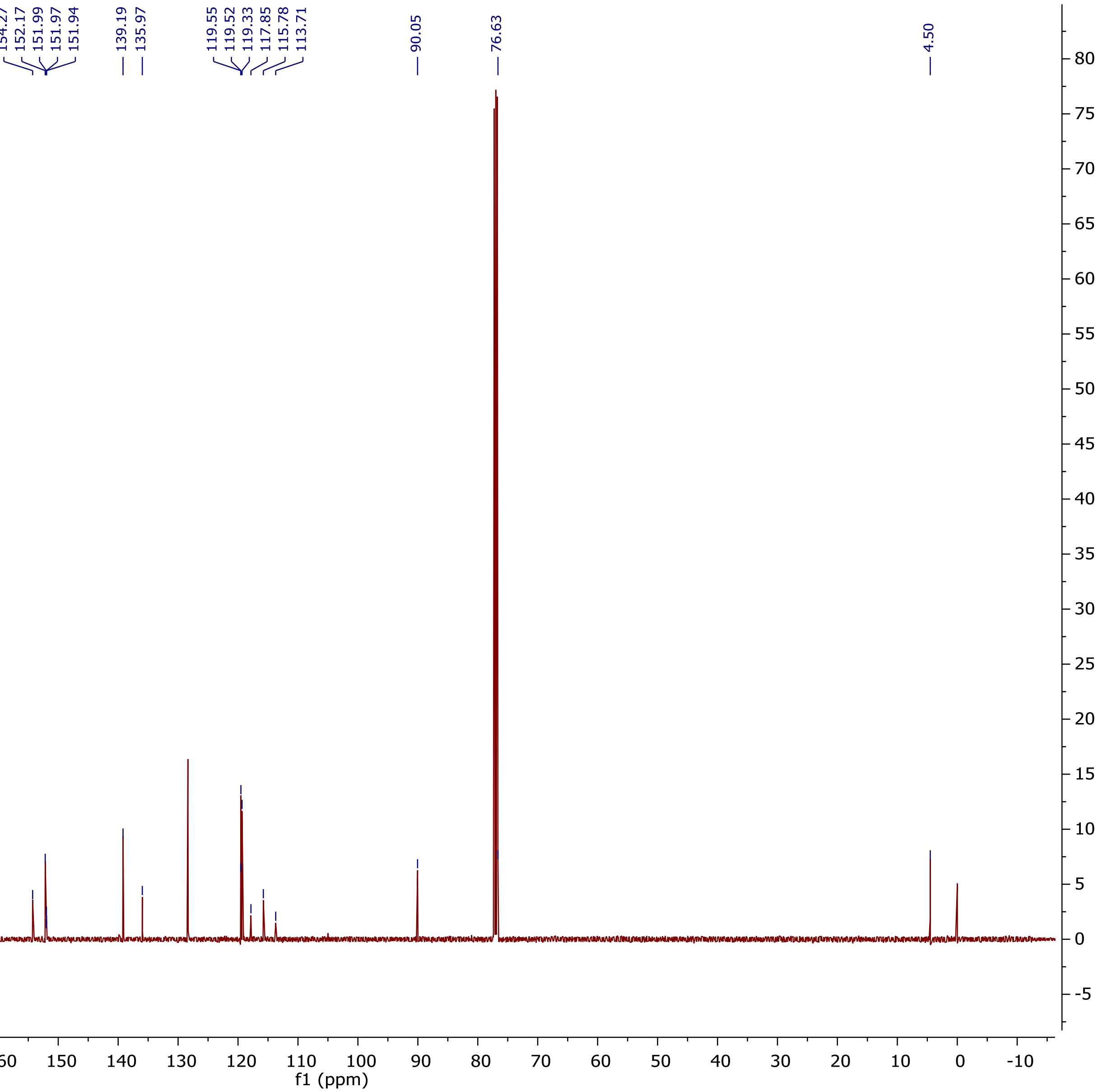

^{19}F NMR (375 MHz, $CDCl_3$)

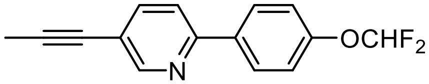

nementin-12-14

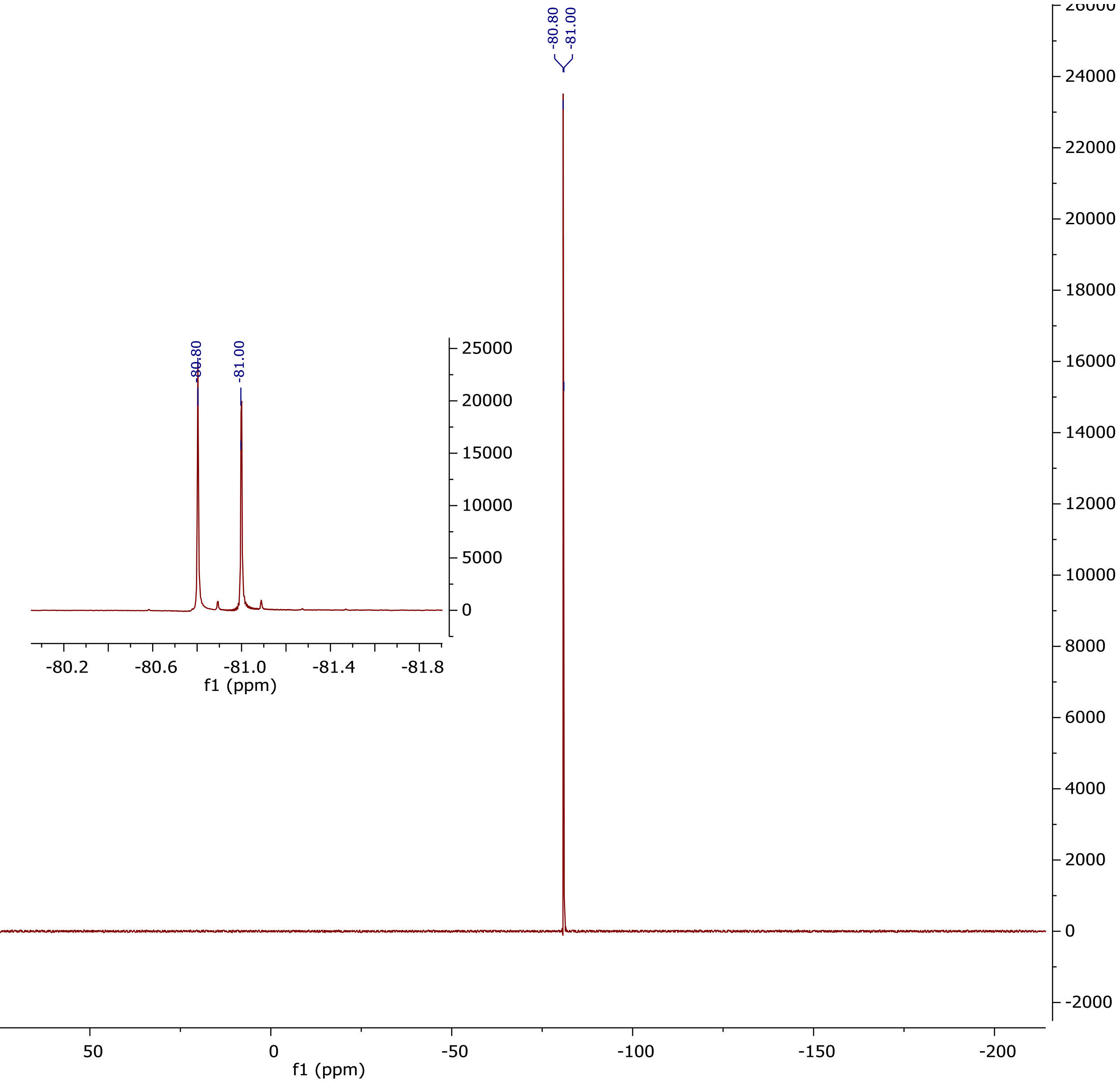

^{1}H NMR (500 MHz, CDCl₃) (nementin-12-15)

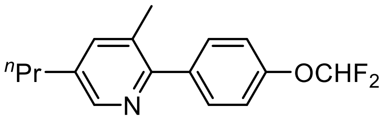

nementin-12-15

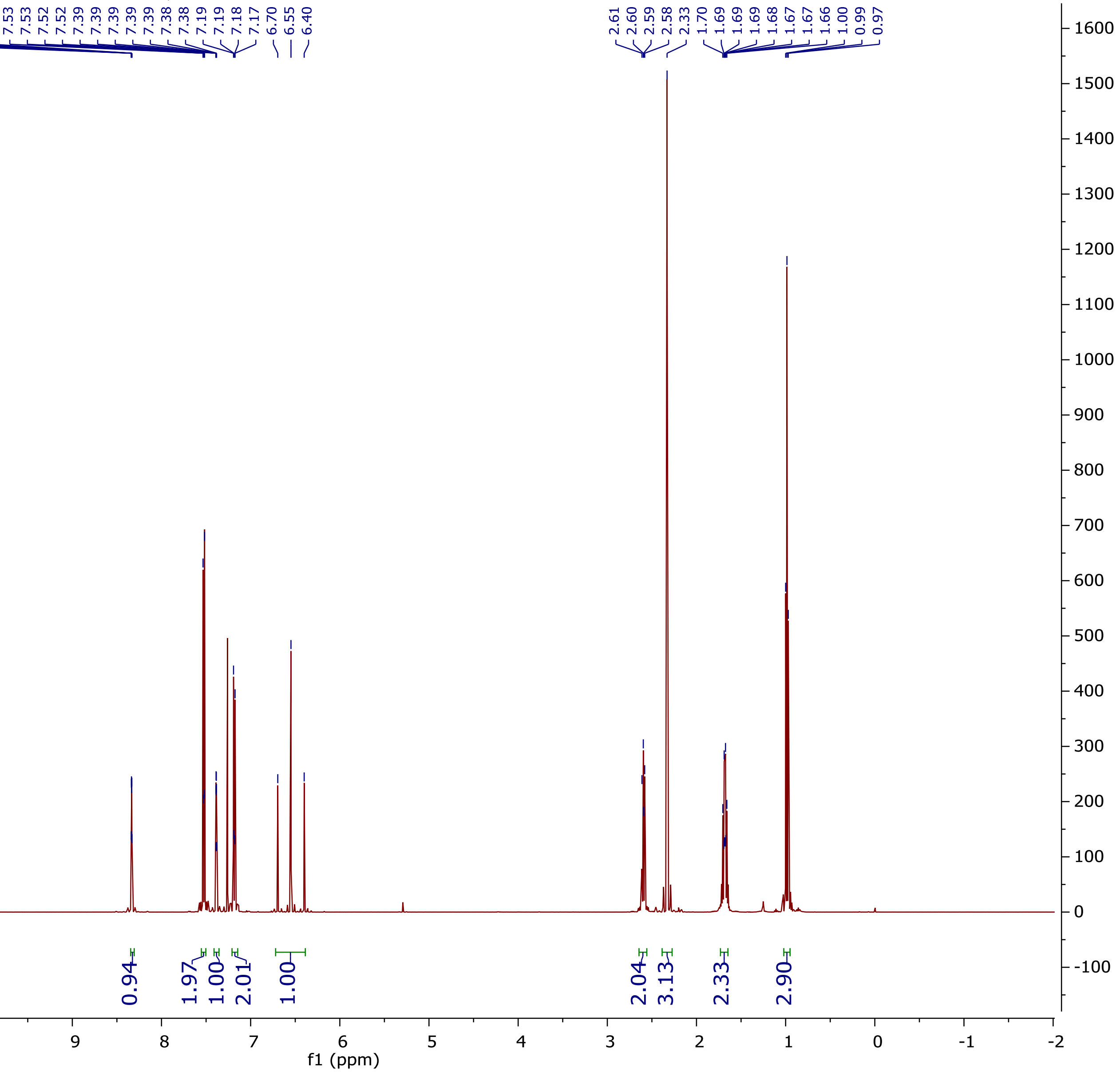

^{13}C NMR (125 MHz, $CDCl_3$)

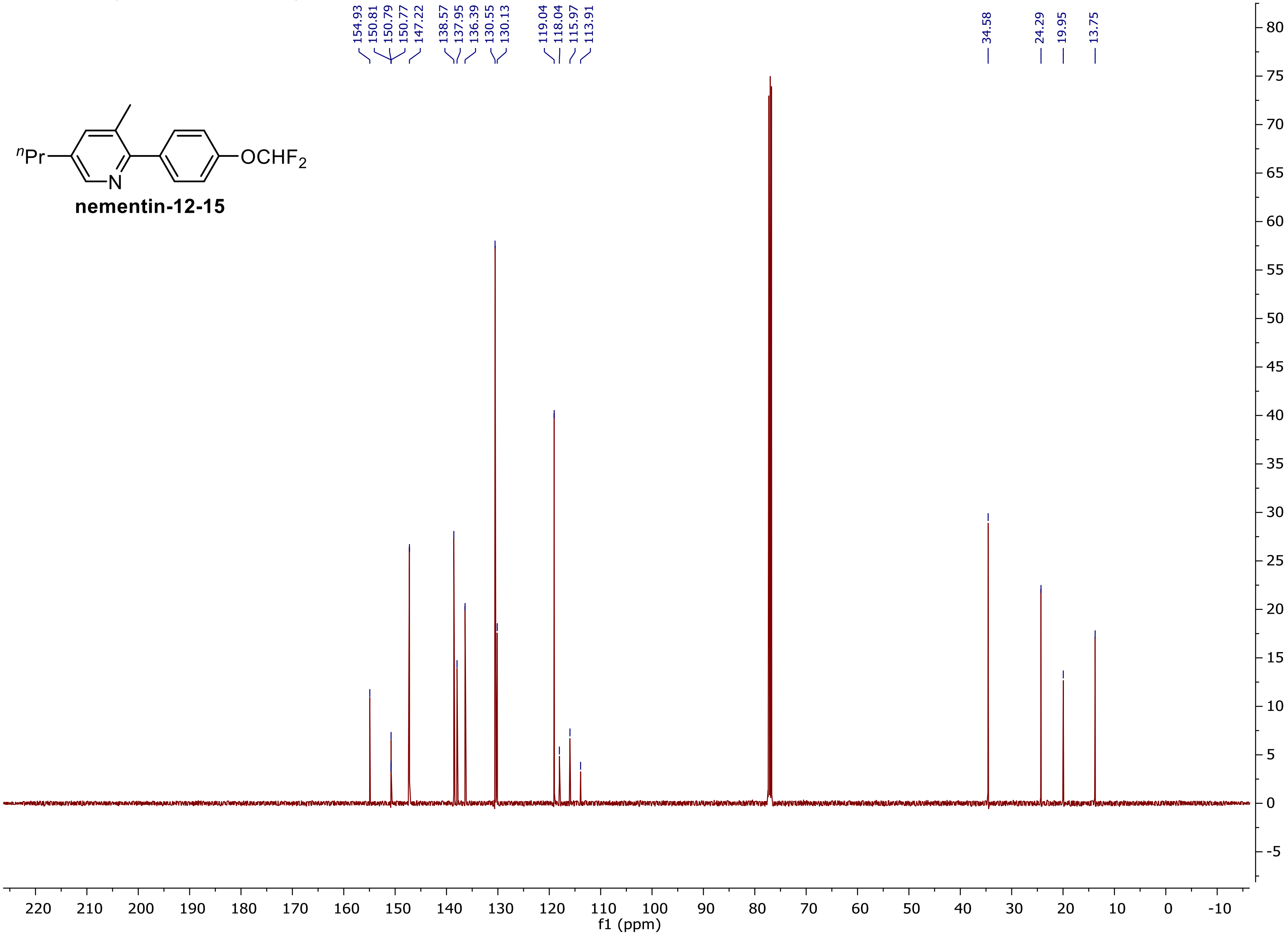

^{19}F NMR (375 MHz, $CDCl_3$)

nementin-12-15

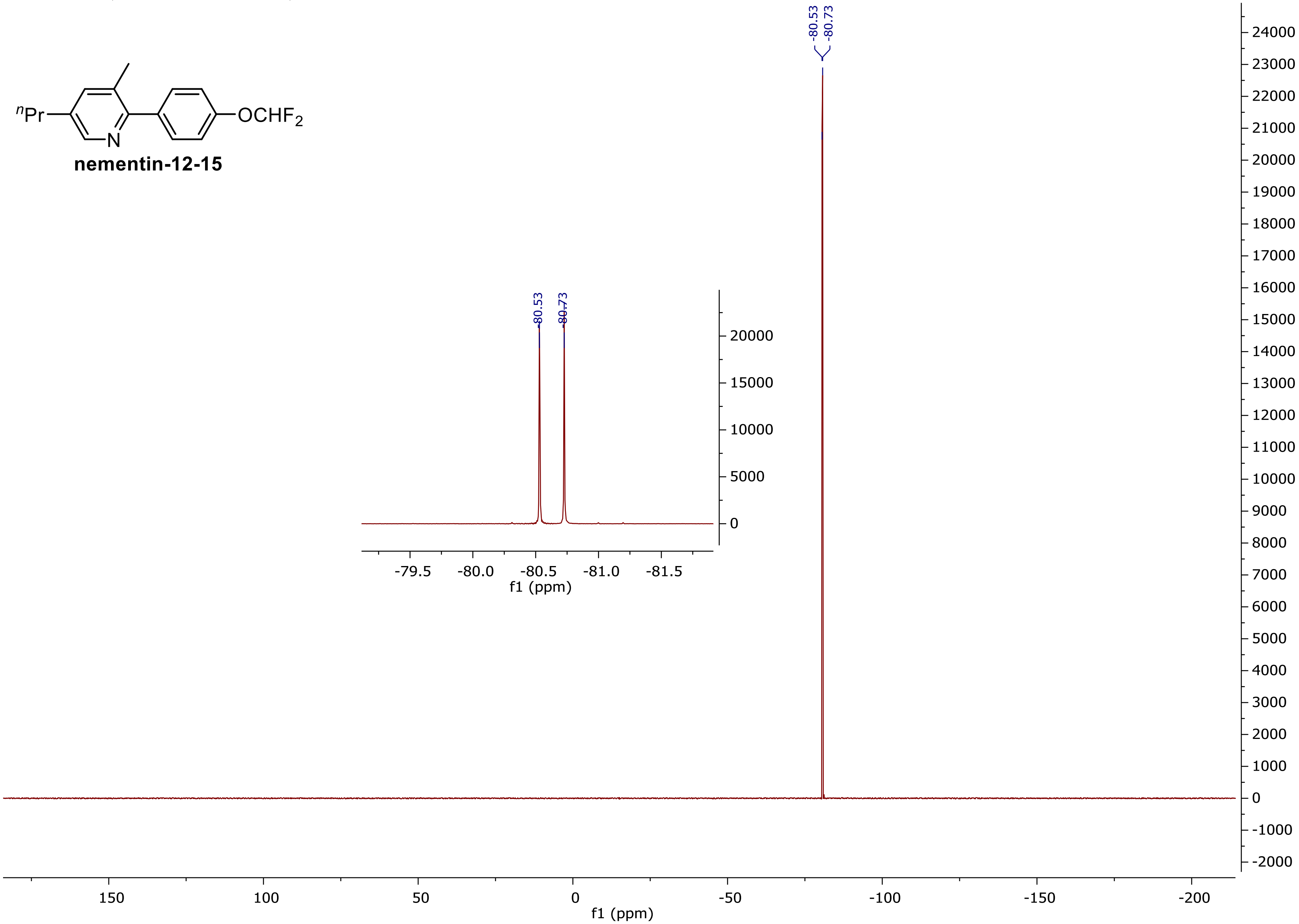

^{1}H NMR (500 MHz, CDCl$_3$) (nementin-12-17)

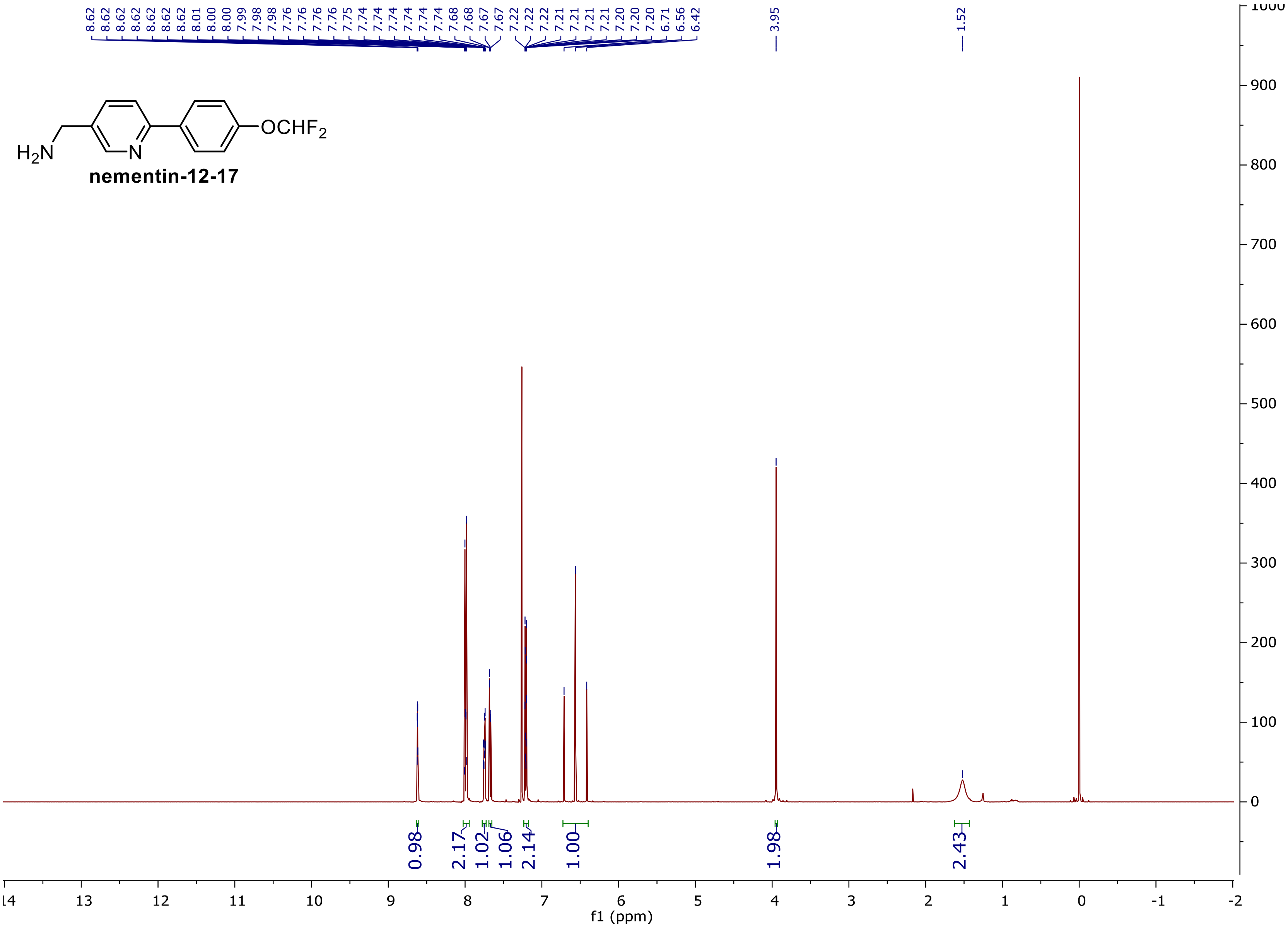

^{13}C NMR (125 MHz, CDCl$_3$)

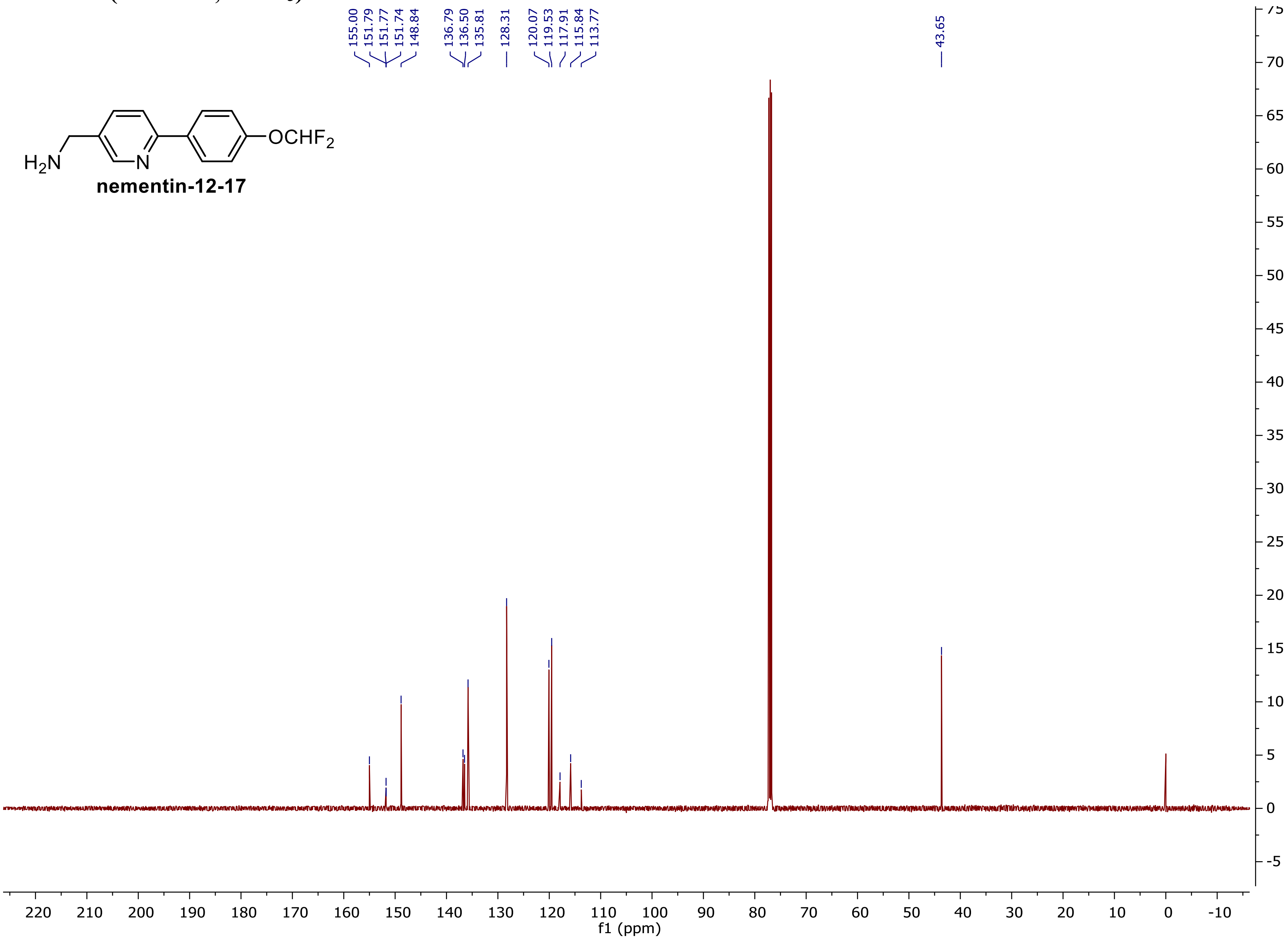

^{19}F NMR (375 MHz, CDCl$_3$)

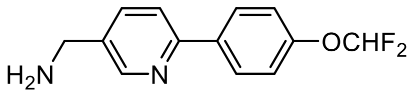

nementin-12-17

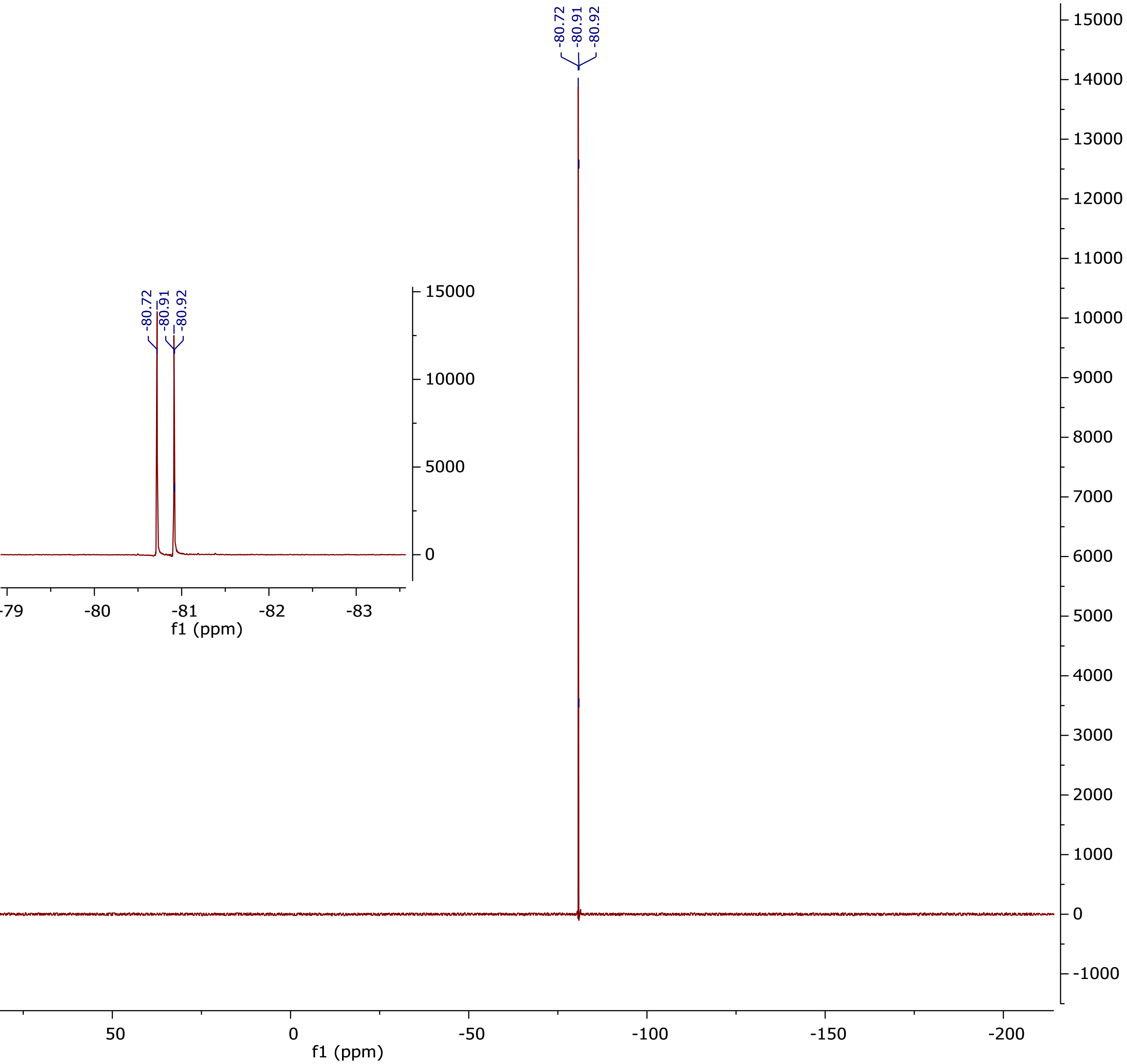

^{1}H NMR (500 MHz, CDCl$_3$) (nementin-12-18)

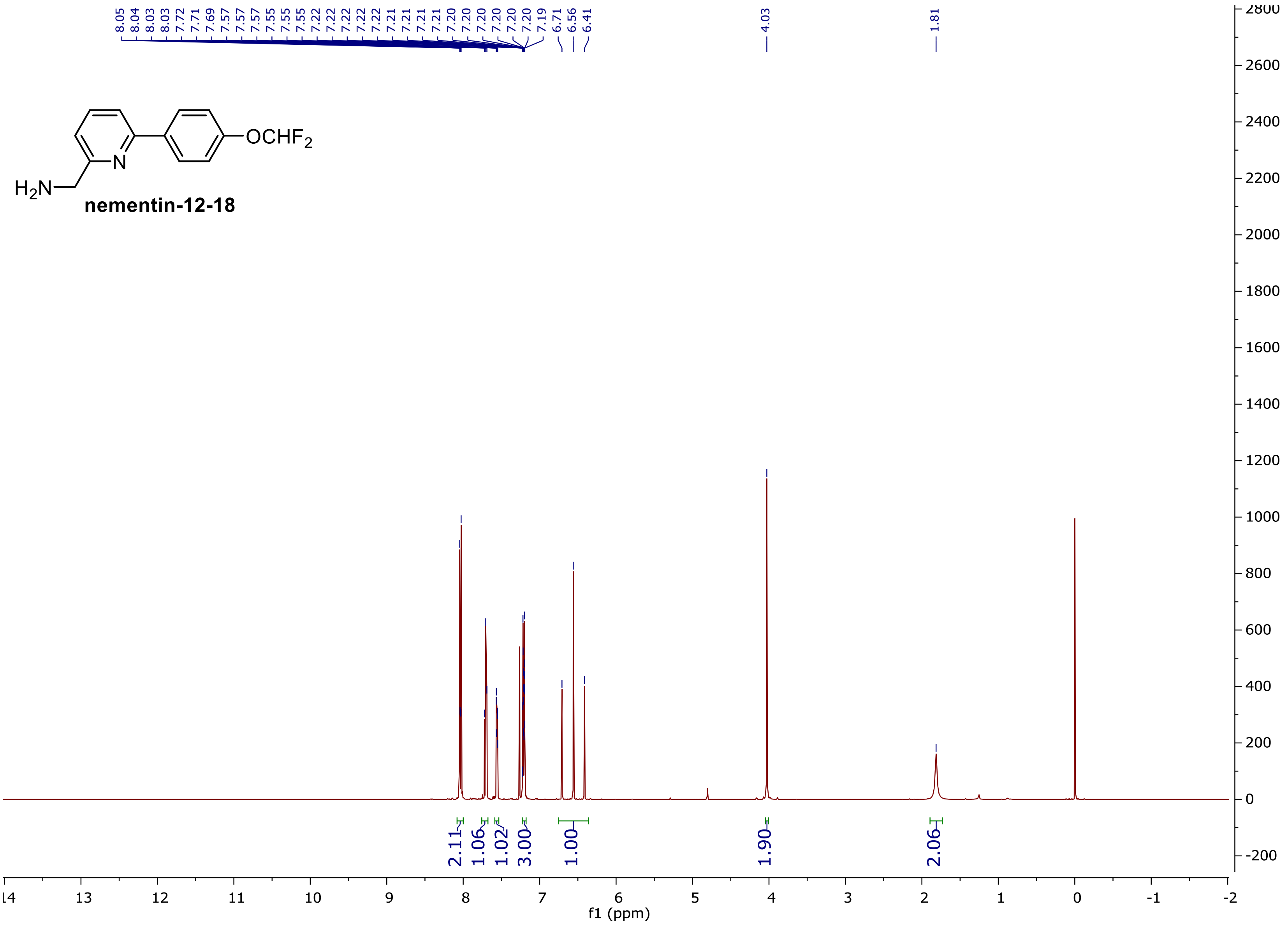

nementin-12-18

^{13}C NMR (125 MHz, $CDCl_3$)

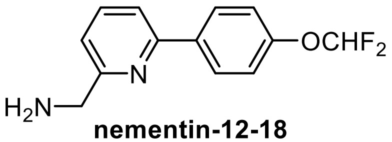

nementin-12-18

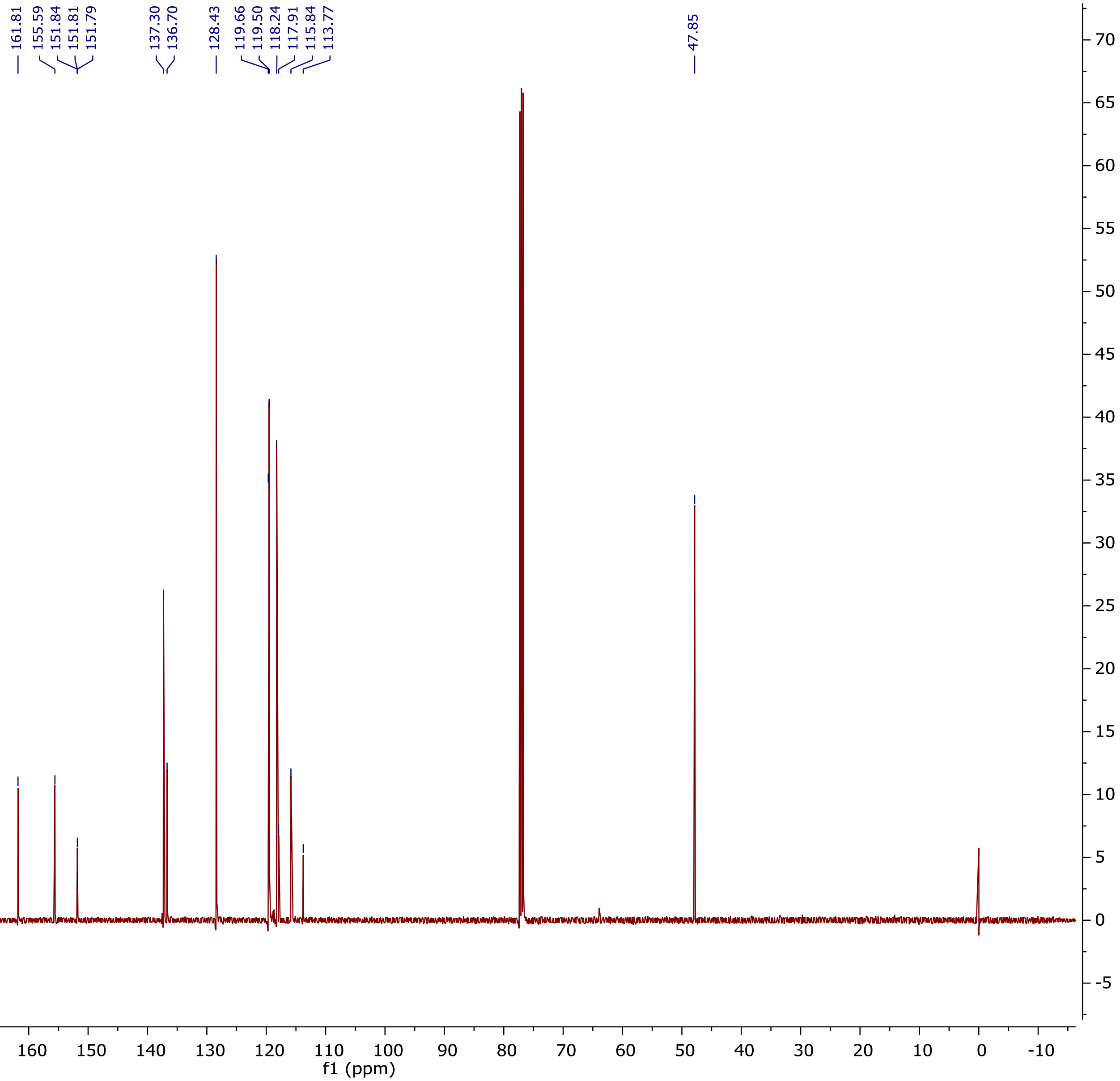

^{19}F NMR (375 MHz, $CDCl_3$)

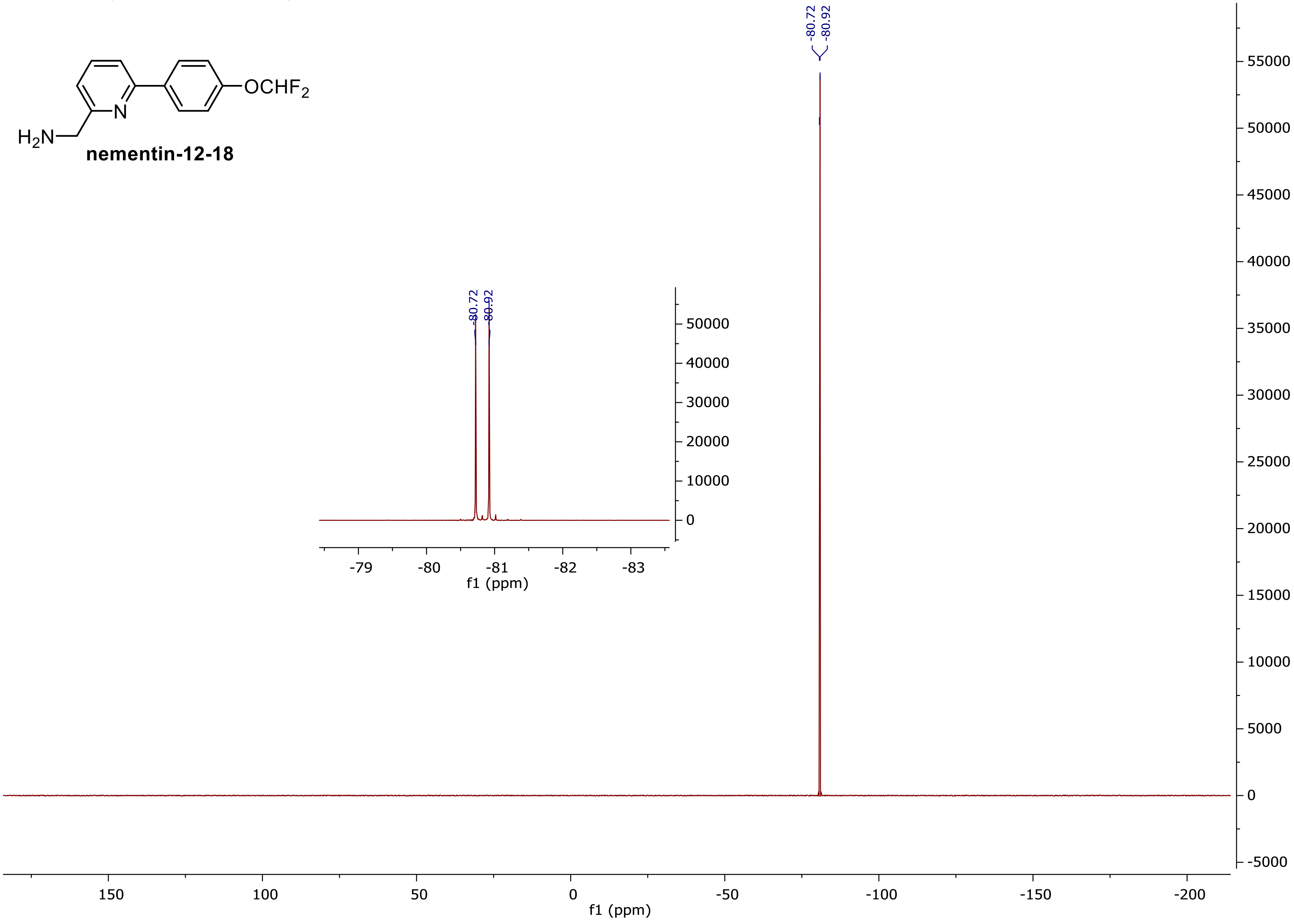

^{1}H NMR (500 MHz, CDCl$_3$) (nementin-12-20)

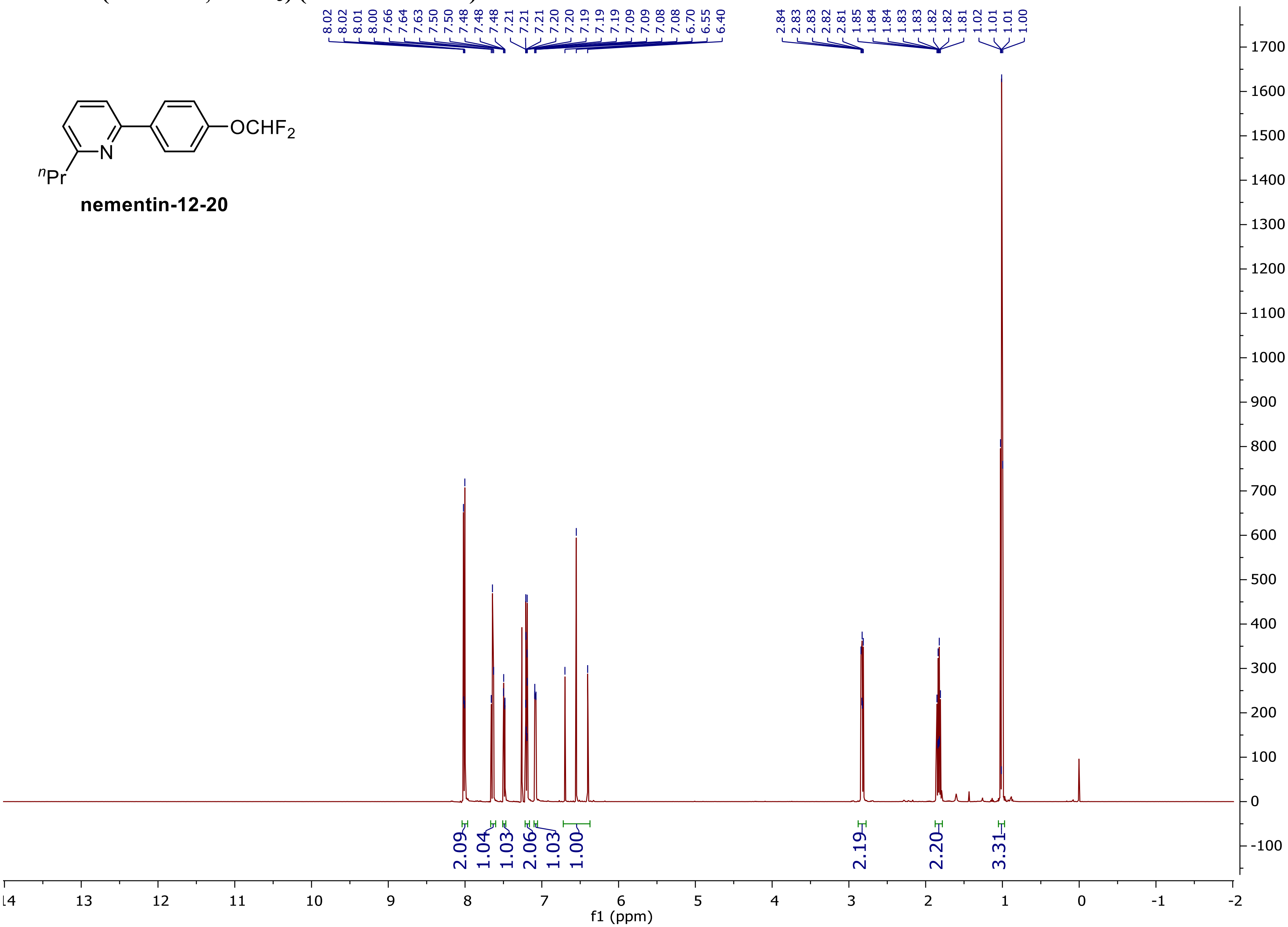

^{13}C NMR (125 MHz, CDCl$_3$)

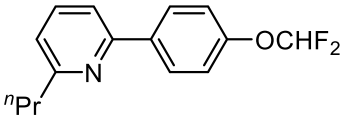

nementin-12-20

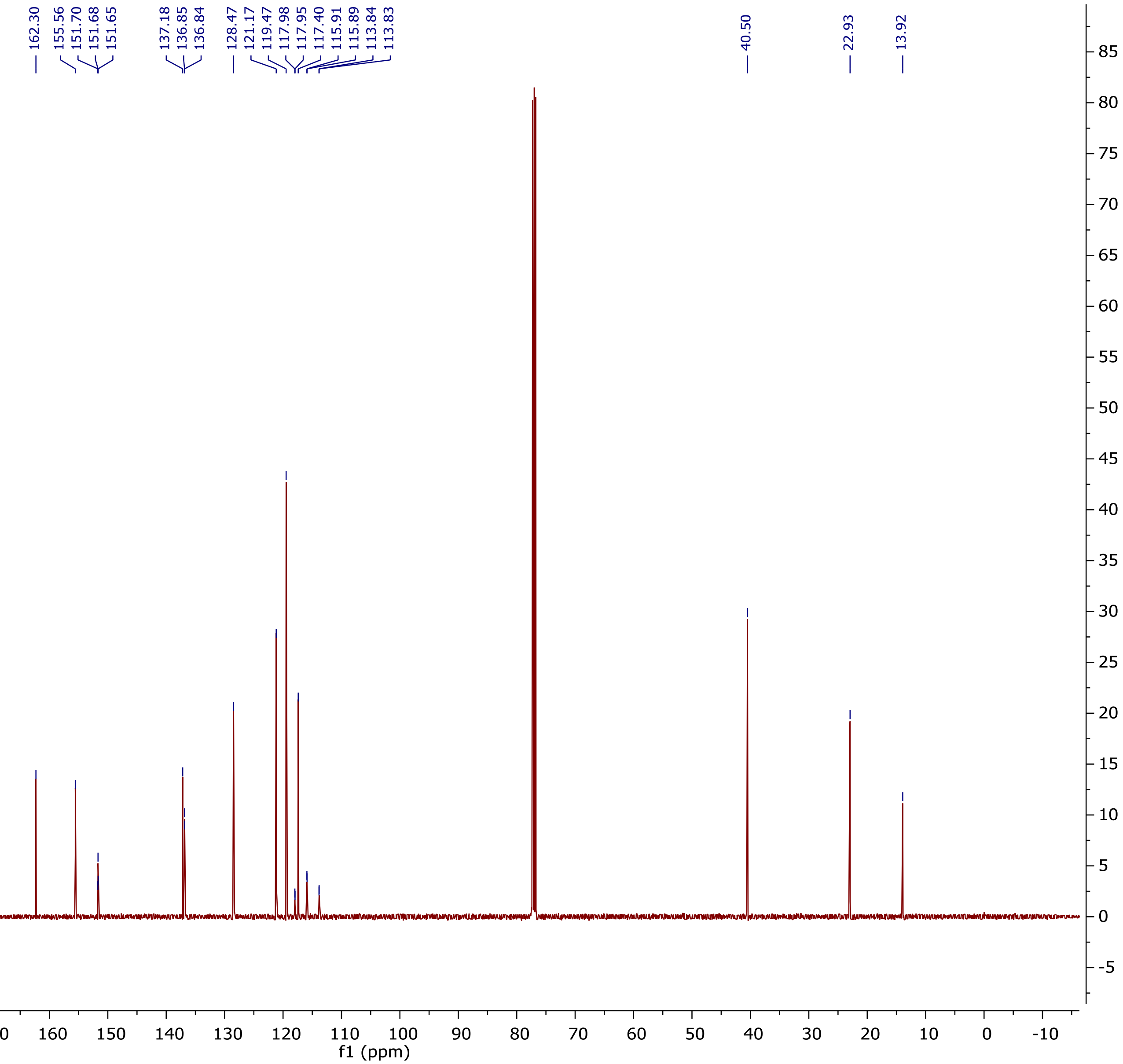

^{19}F NMR (375 MHz, $CDCl_3$)

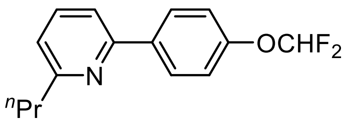

nementin-12-20

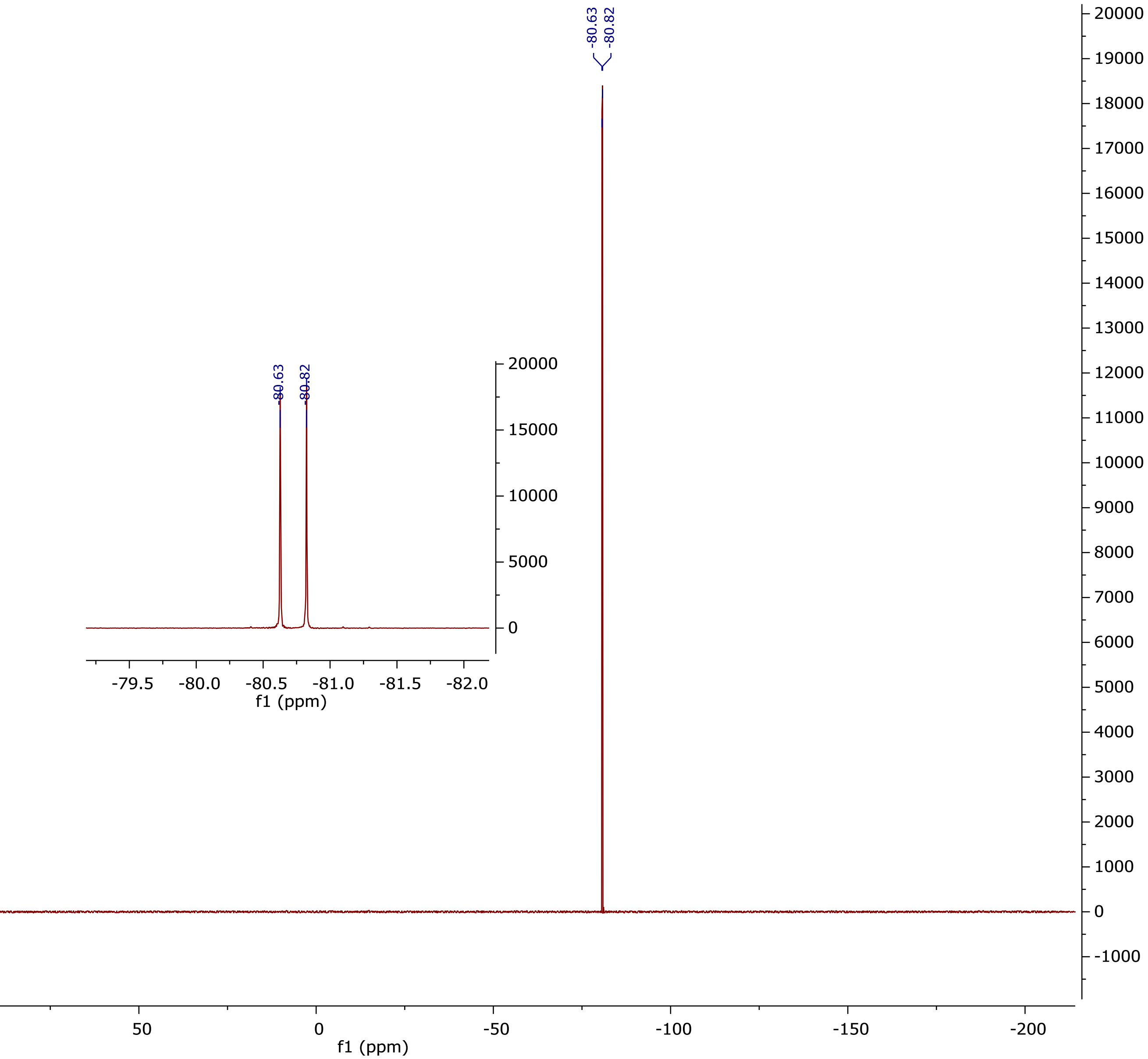

^{1}H NMR (500 MHz, $CDCl_3$) (nementin-12-21)

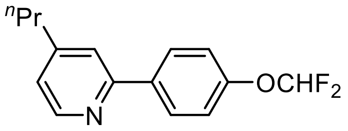
nementin-12-21

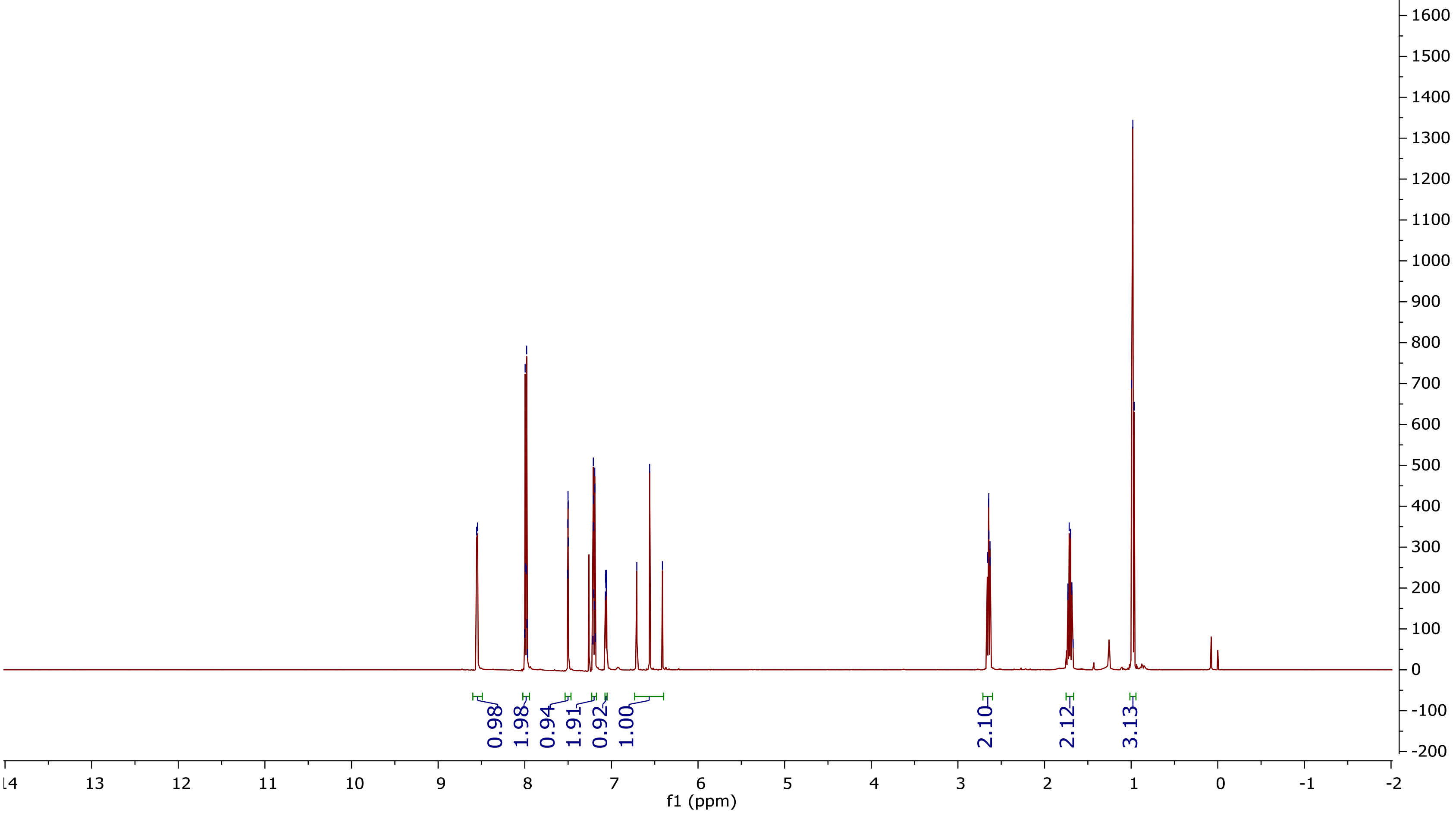

^{13}C NMR (125 MHz, CDCl$_3$)

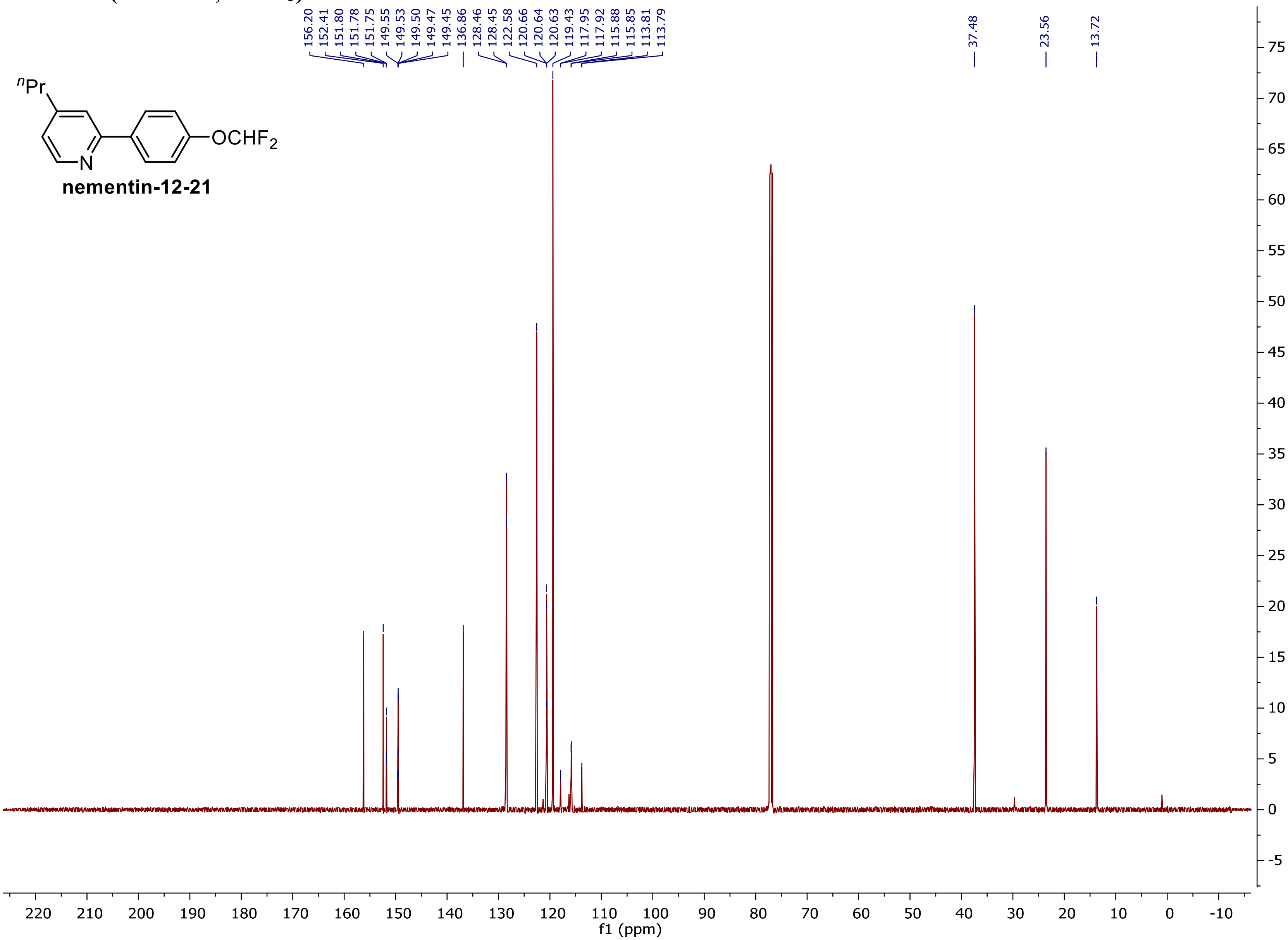

^{19}F NMR (375 MHz, $CDCl_3$)

nementin-12-21

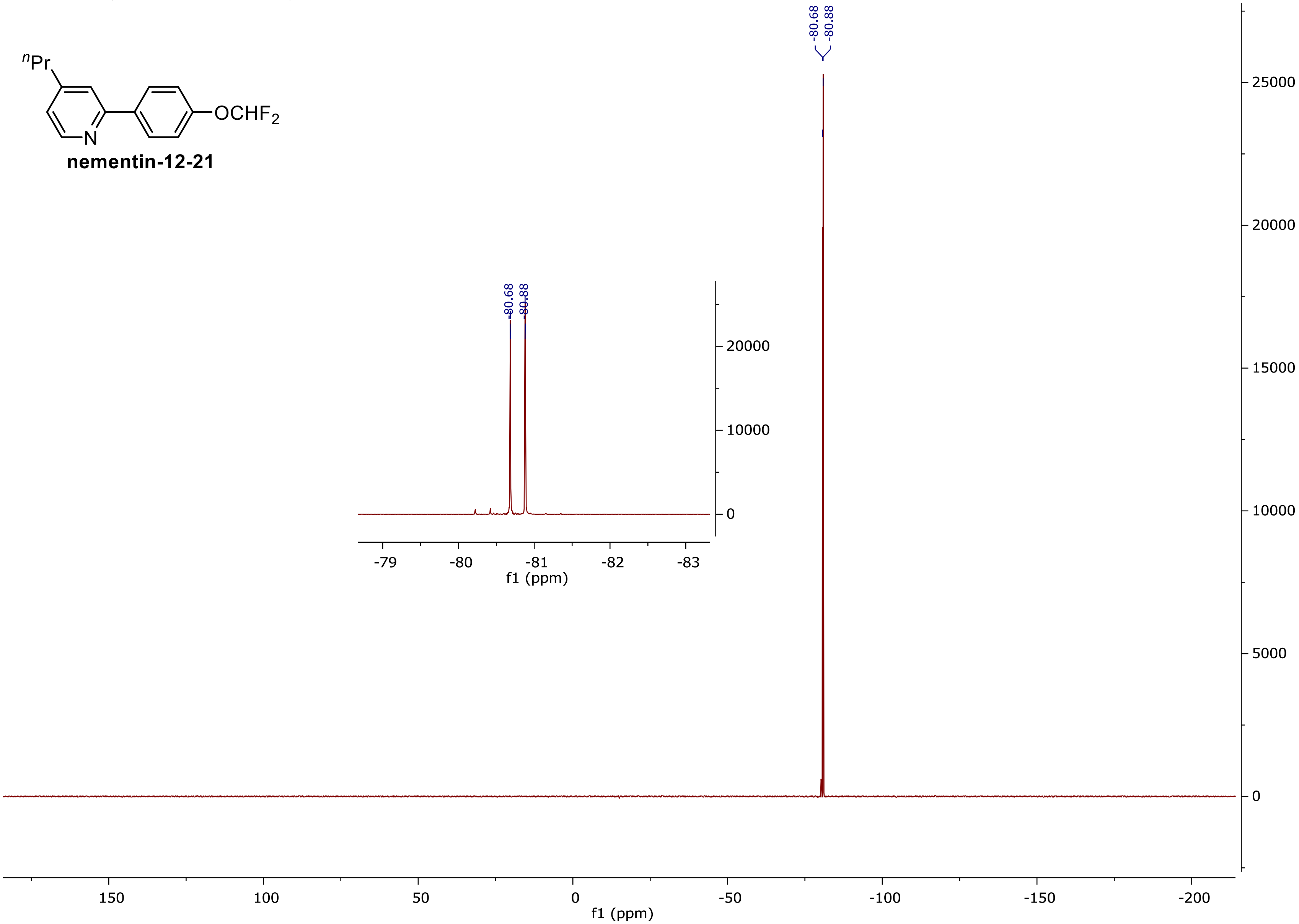

^{1}H NMR (500 MHz, CDCl₃) (nementin-12-22)

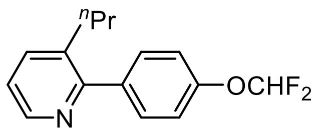

nementin-12-22

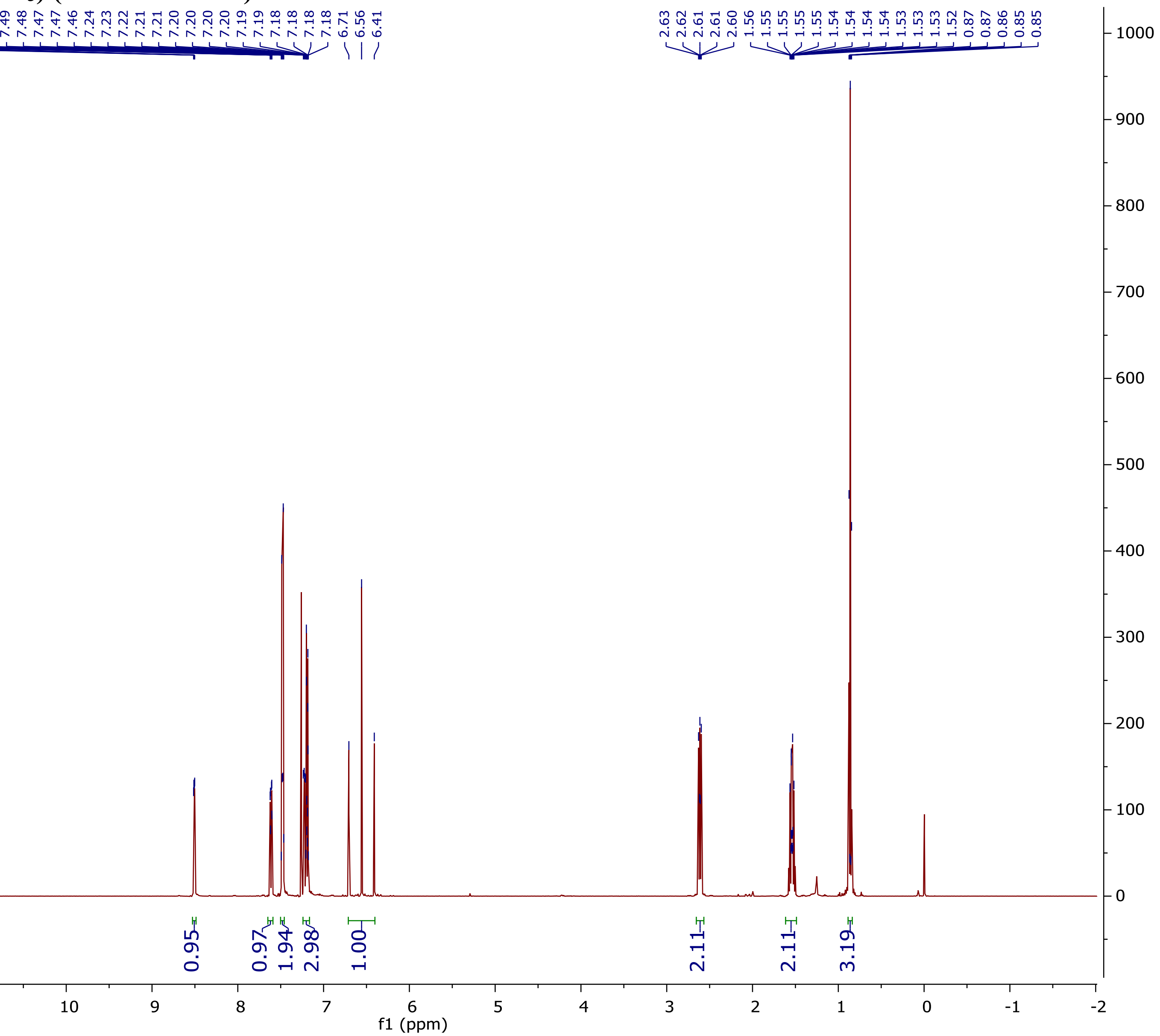

^{13}C NMR (125 MHz, $CDCl_3$)

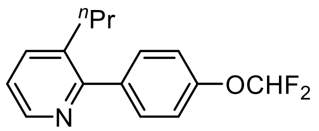

nementin-12-22

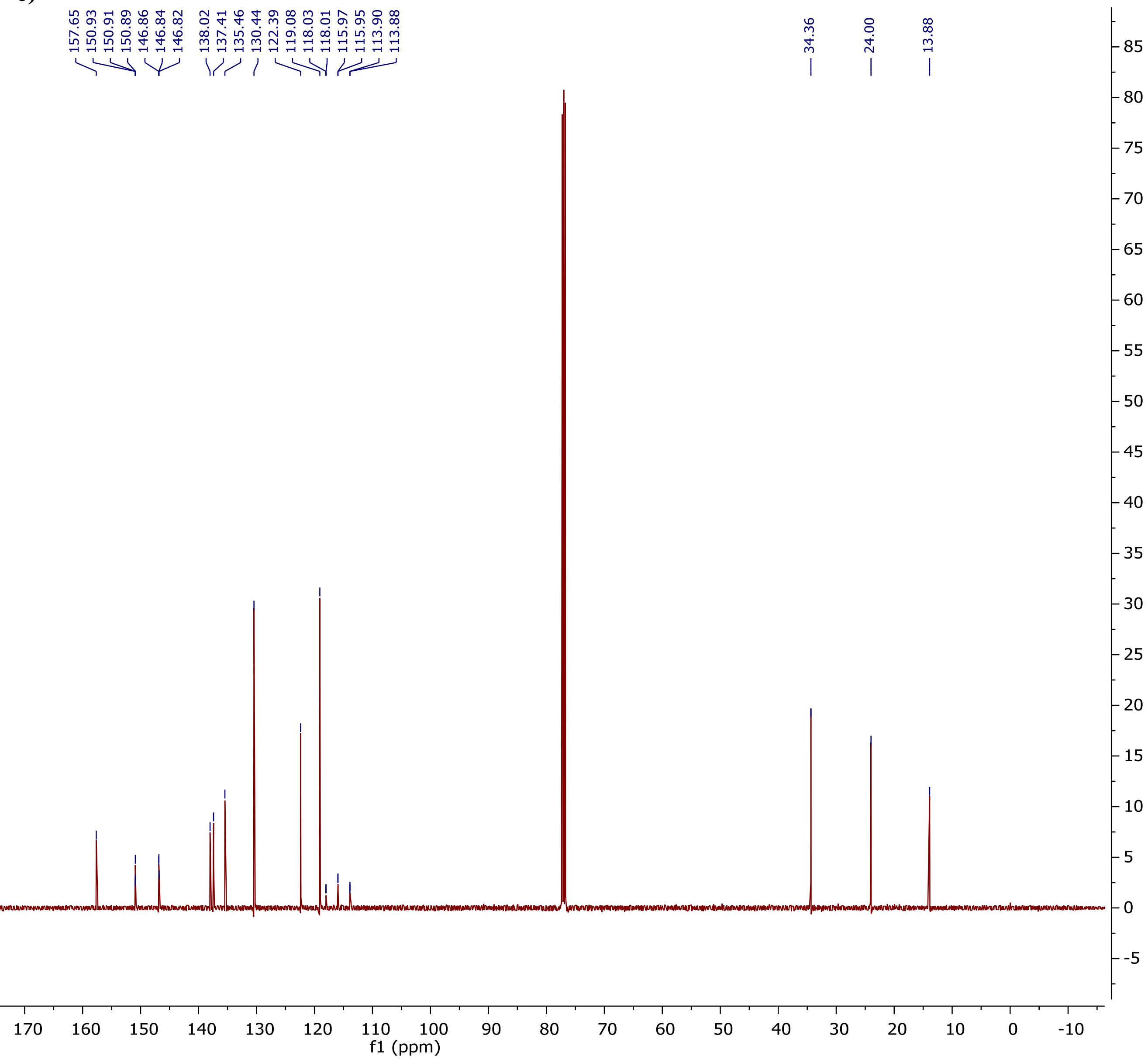

^{19}F NMR (375 MHz, $CDCl_3$)

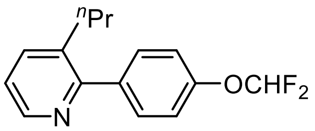

nementin-12-22

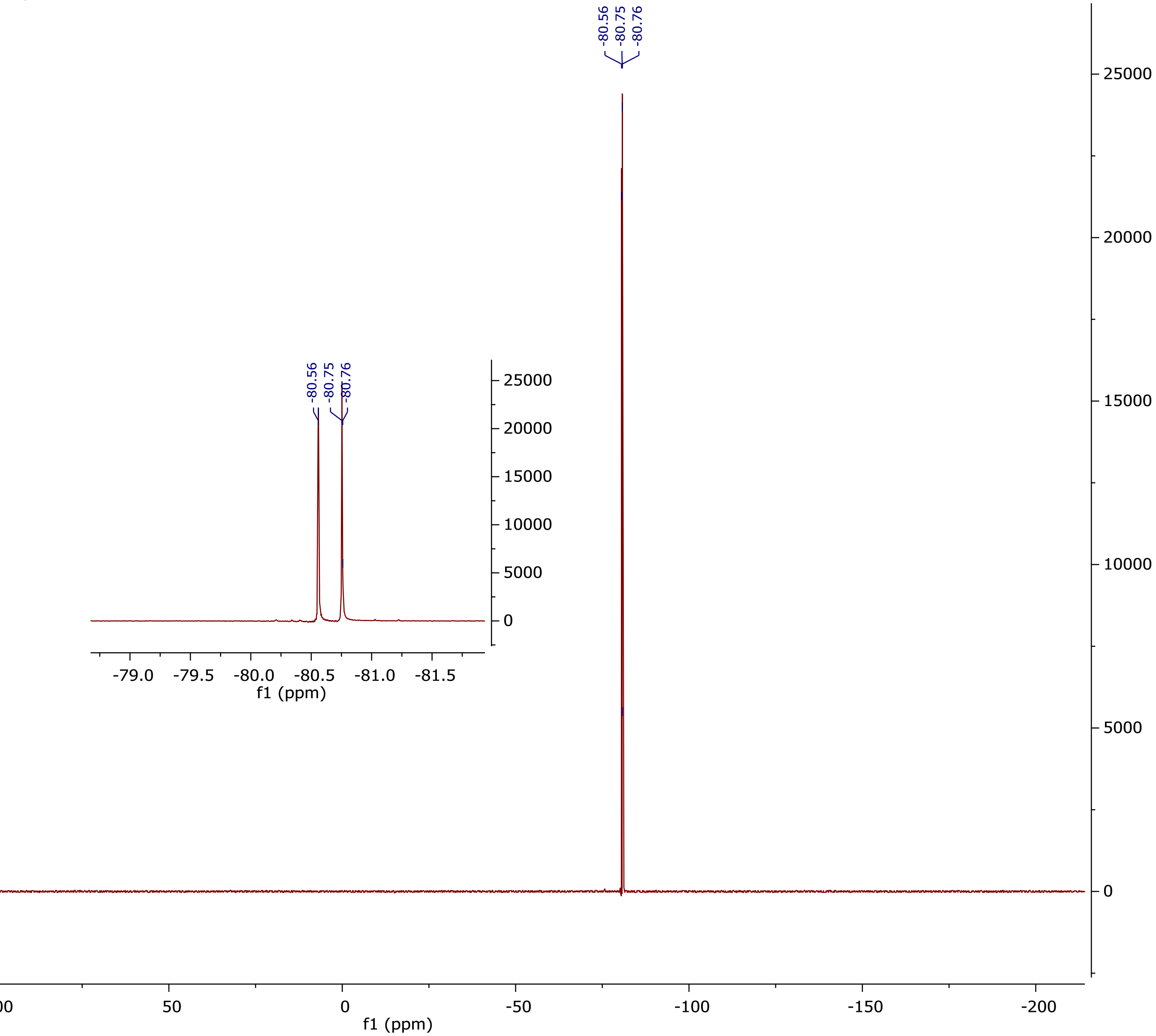

^{1}H NMR (500 MHz, $CDCl_3$) (nementin-12-23)

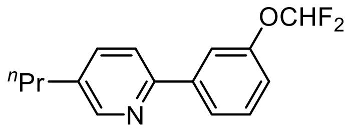

nementin-12-23

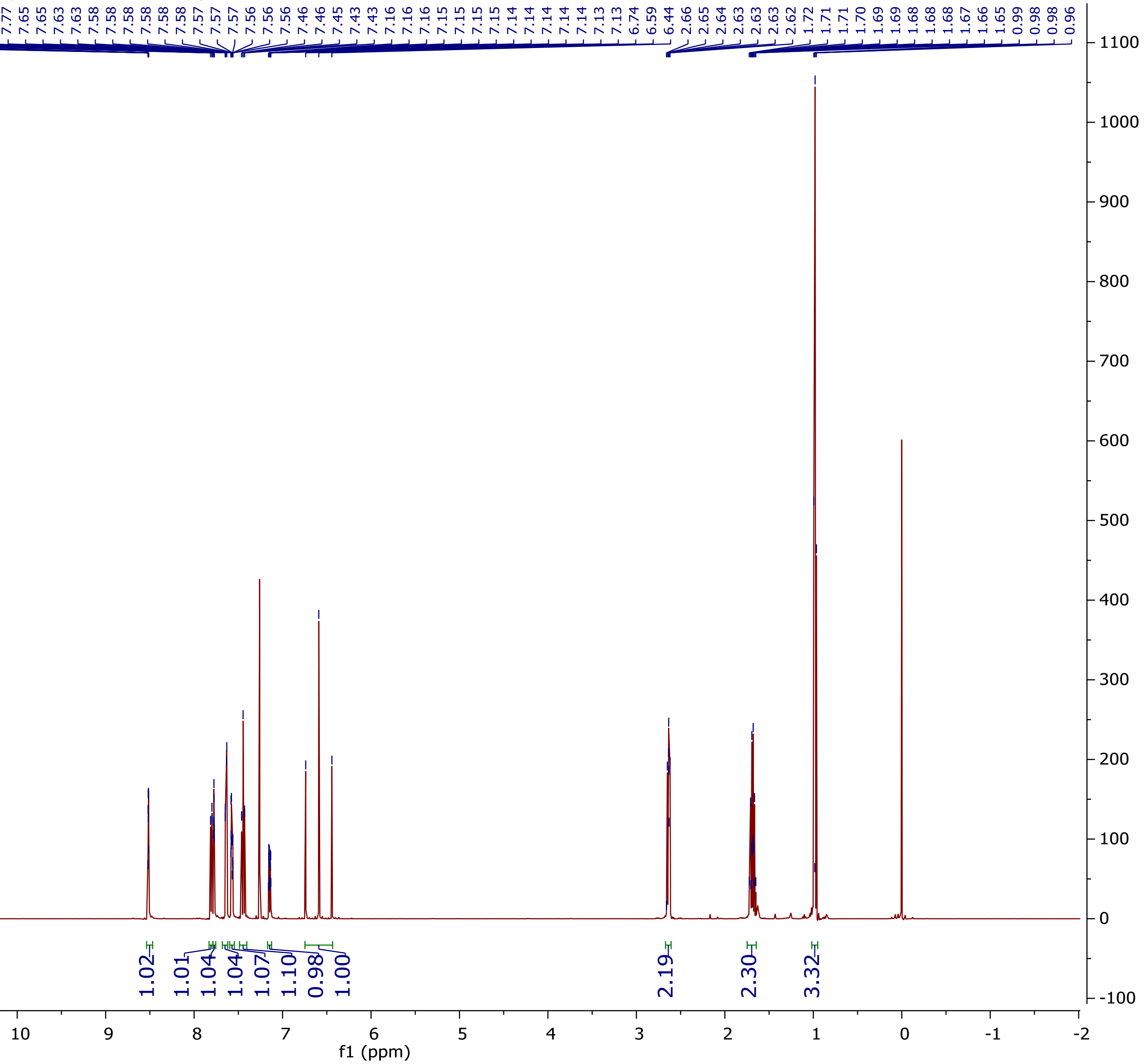

^{13}C NMR (125 MHz, $CDCl_3$)

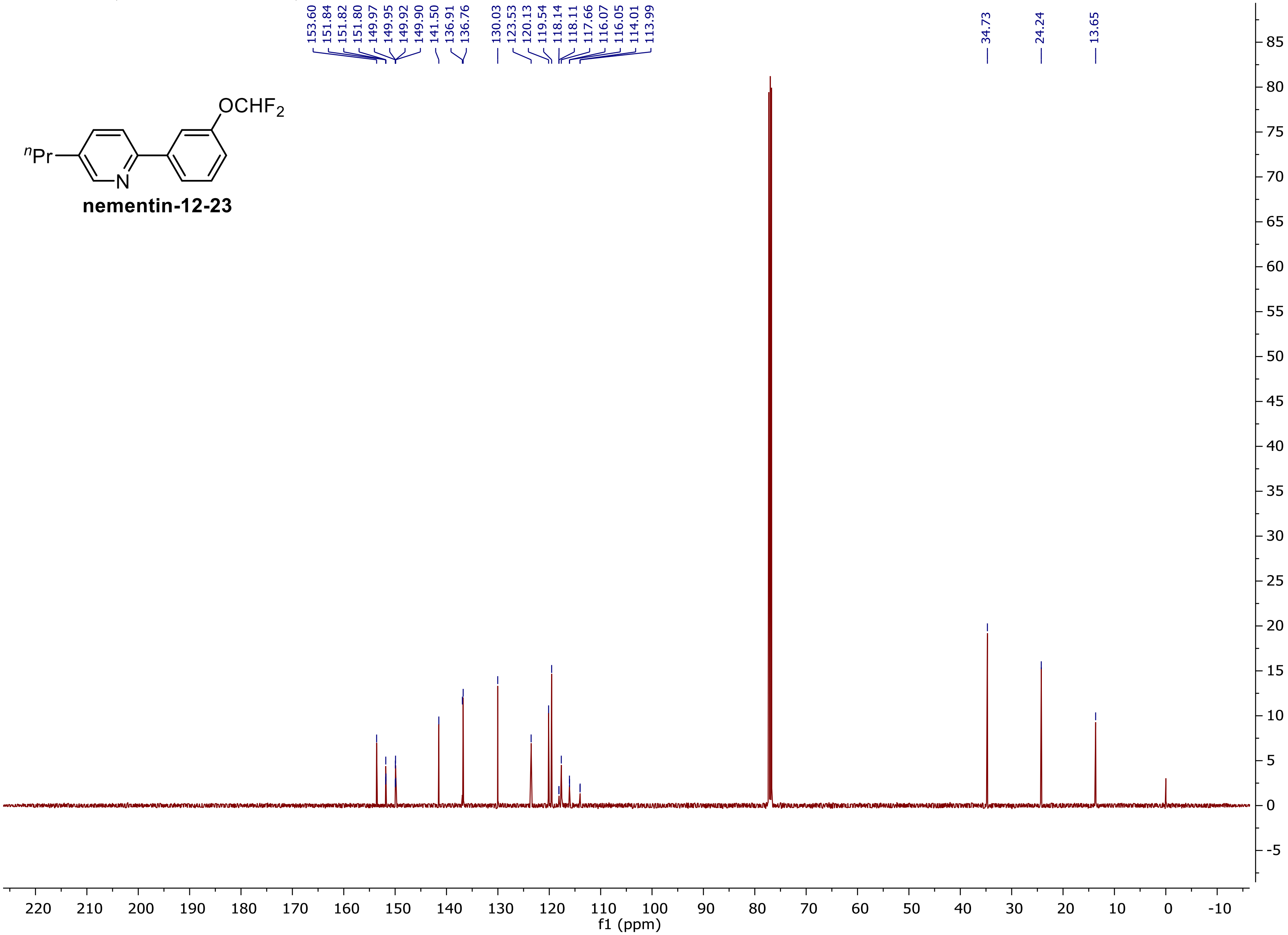

nementin-12-23

^{19}F NMR (375 MHz, $CDCl_3$)

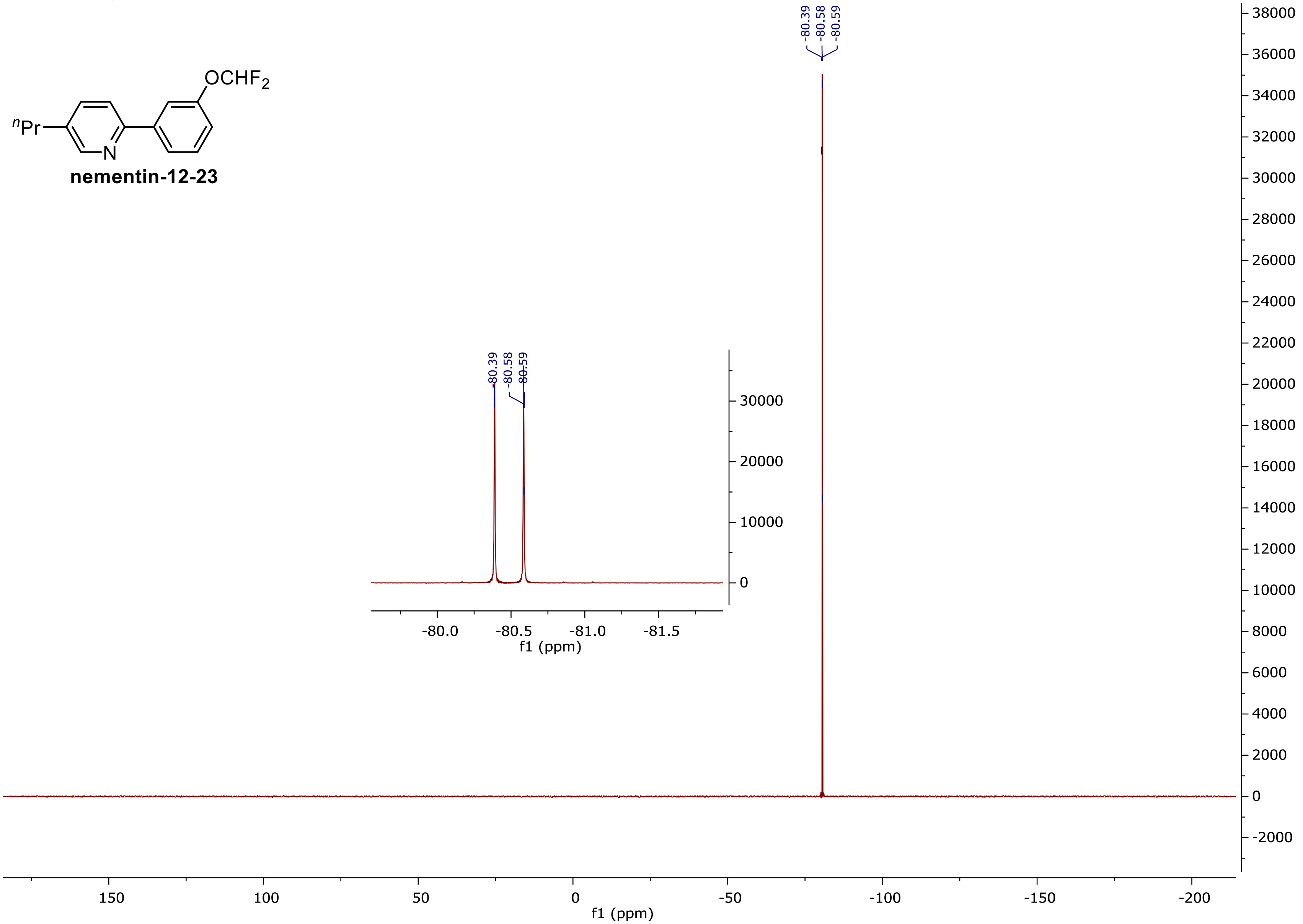

^{1}H NMR (500 MHz, CDCl$_3$) (nementin-12-24)

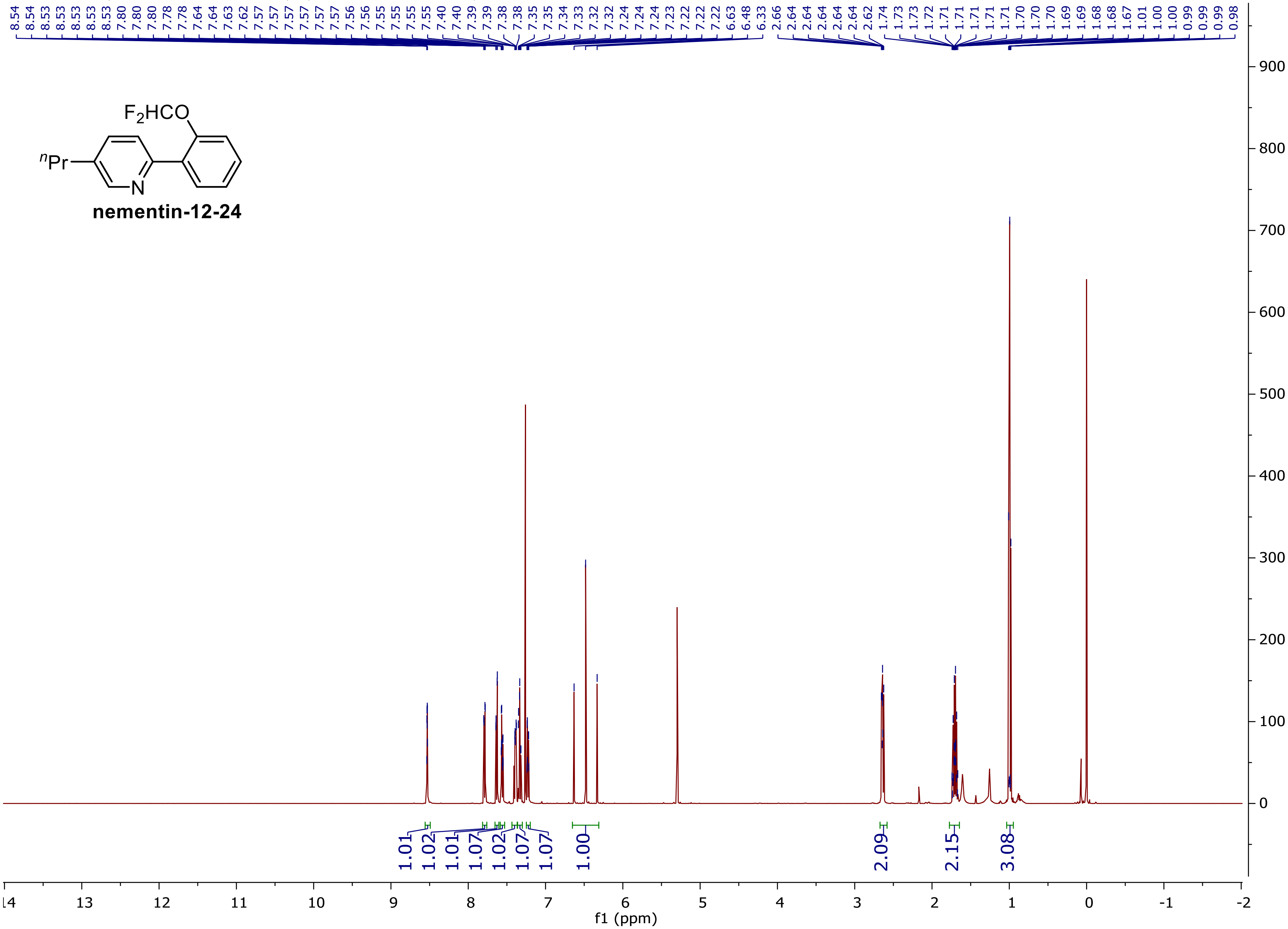

^{13}C NMR (125 MHz, $CDCl_3$)

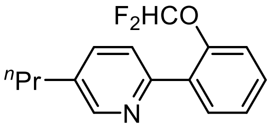

nementin-12-24

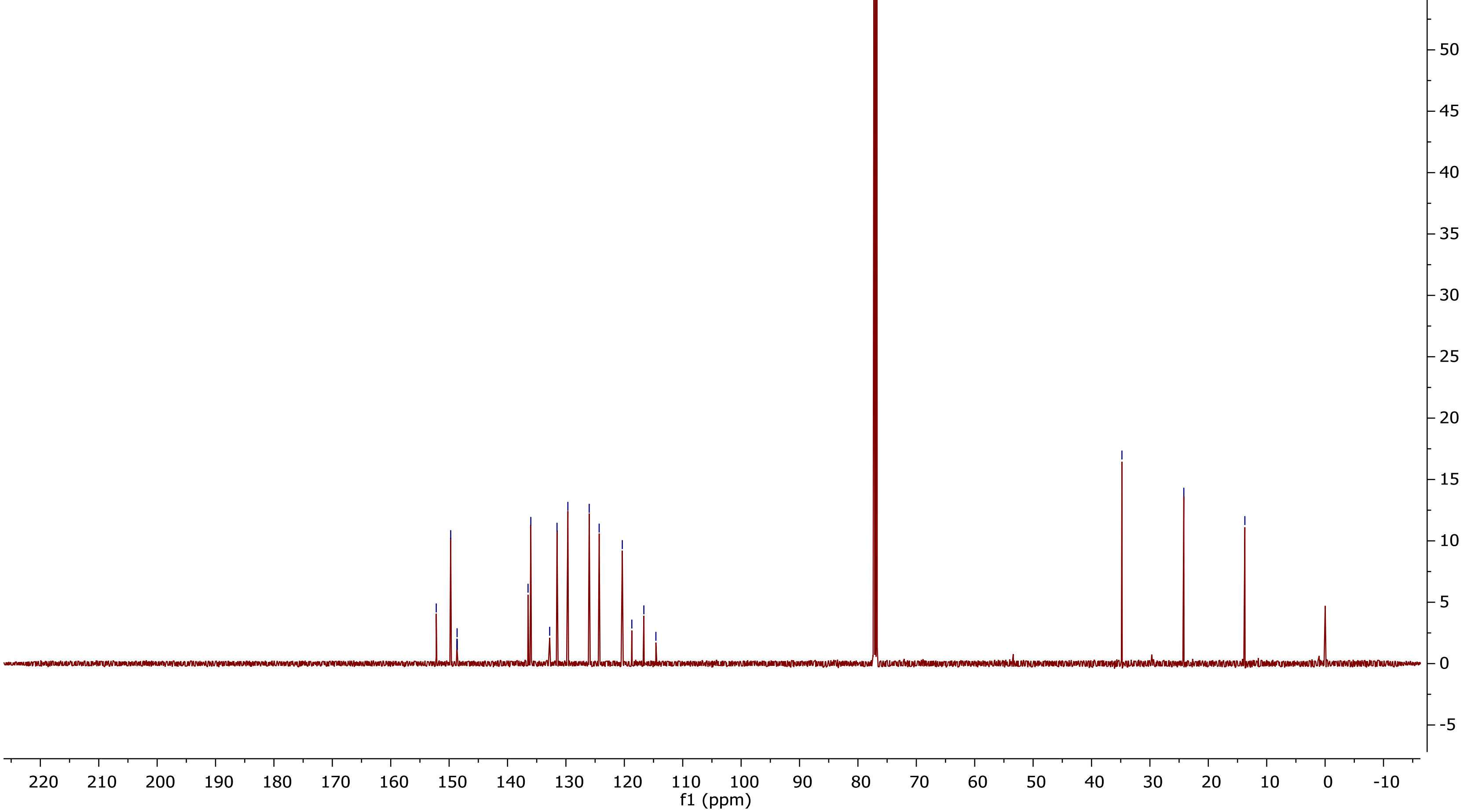

^{19}F NMR (375 MHz, $CDCl_3$)

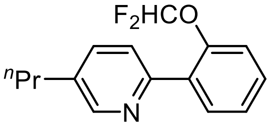

nementin-12-24

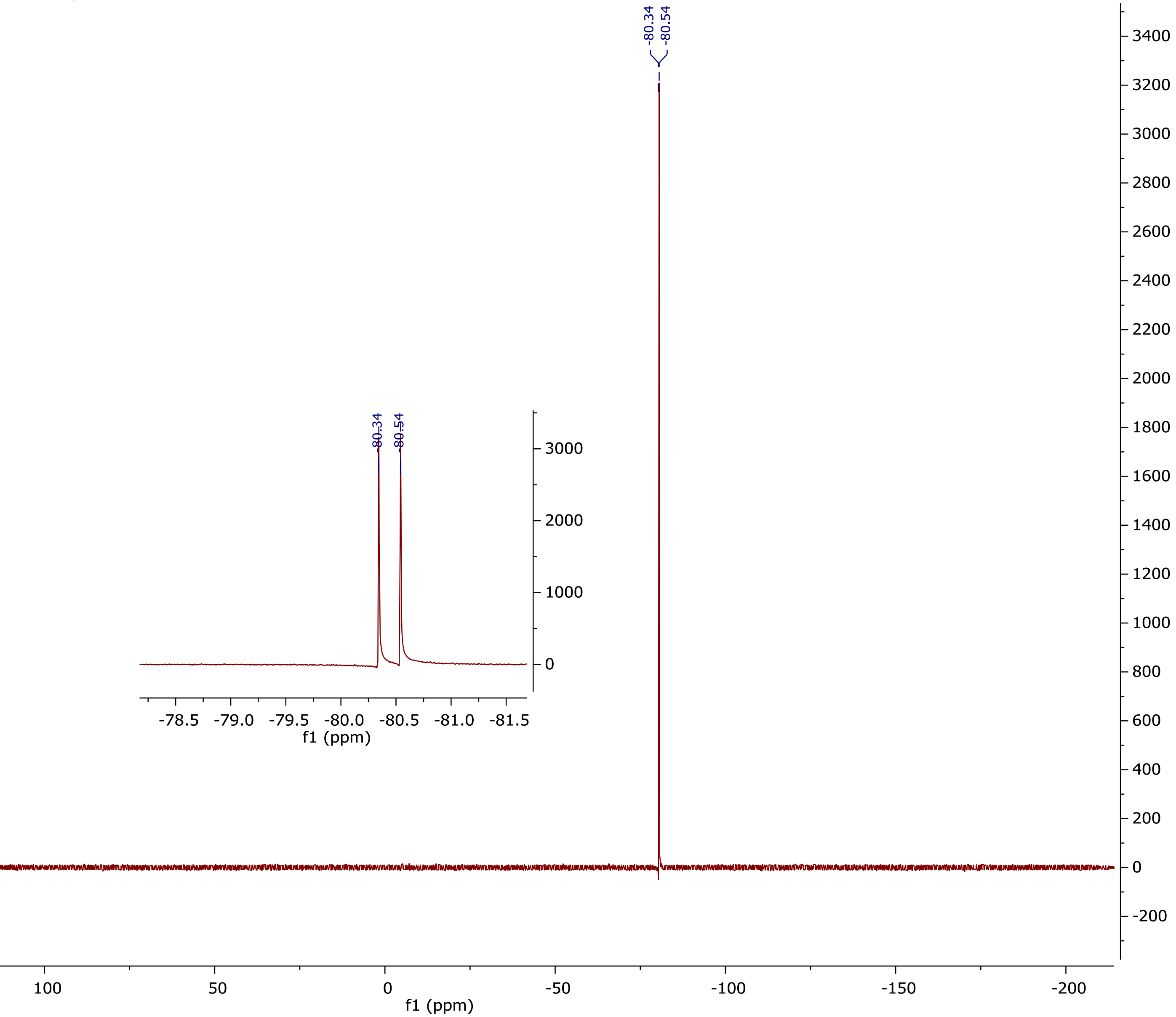

^{1}H NMR (500 MHz, CDCl₃) (nementin-12-25)

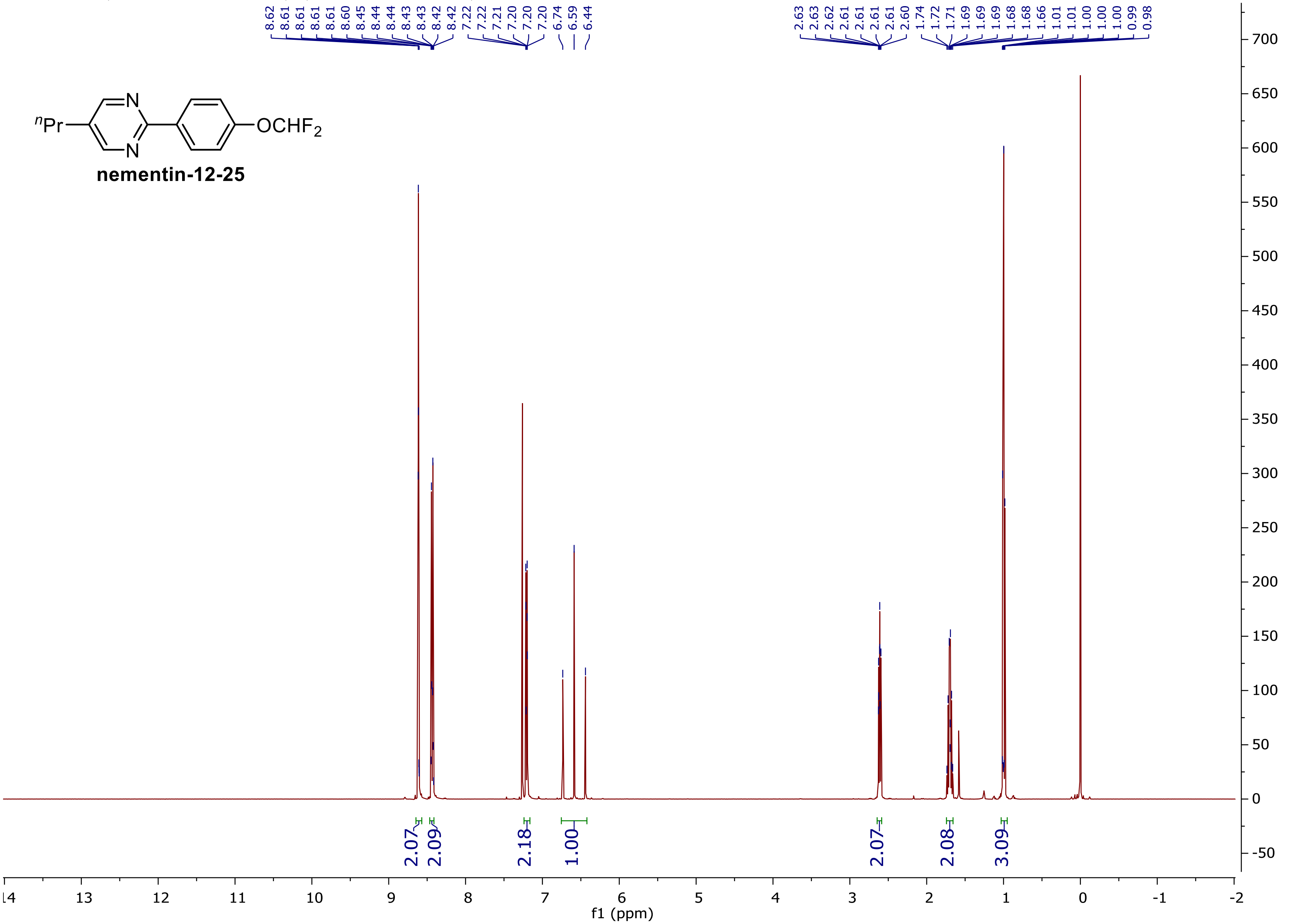

^{13}C NMR (125 MHz, CDCl$_3$)

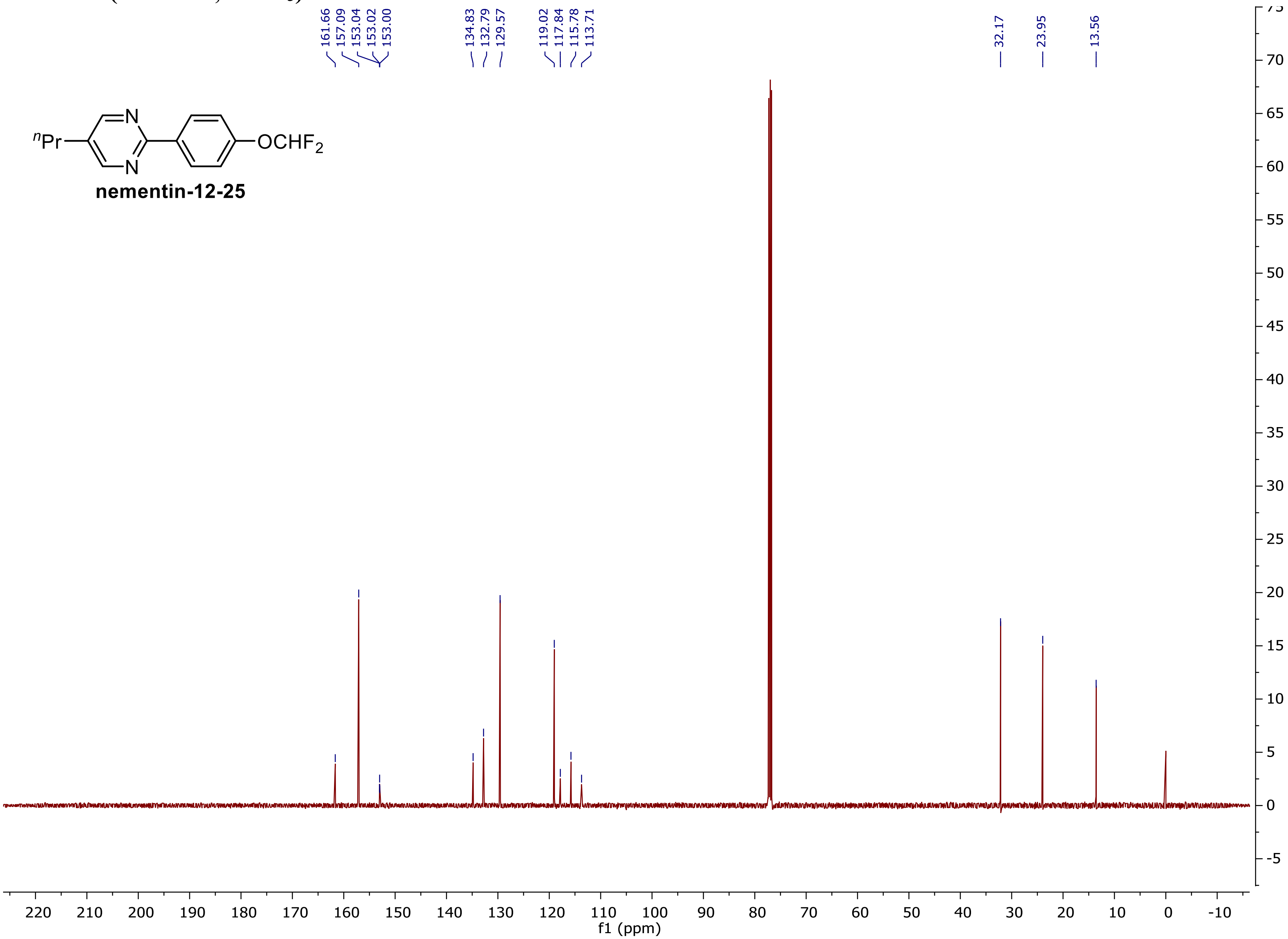

^{19}F NMR (375 MHz, $CDCl_3$)

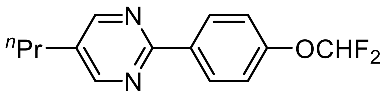

nementin-12-25

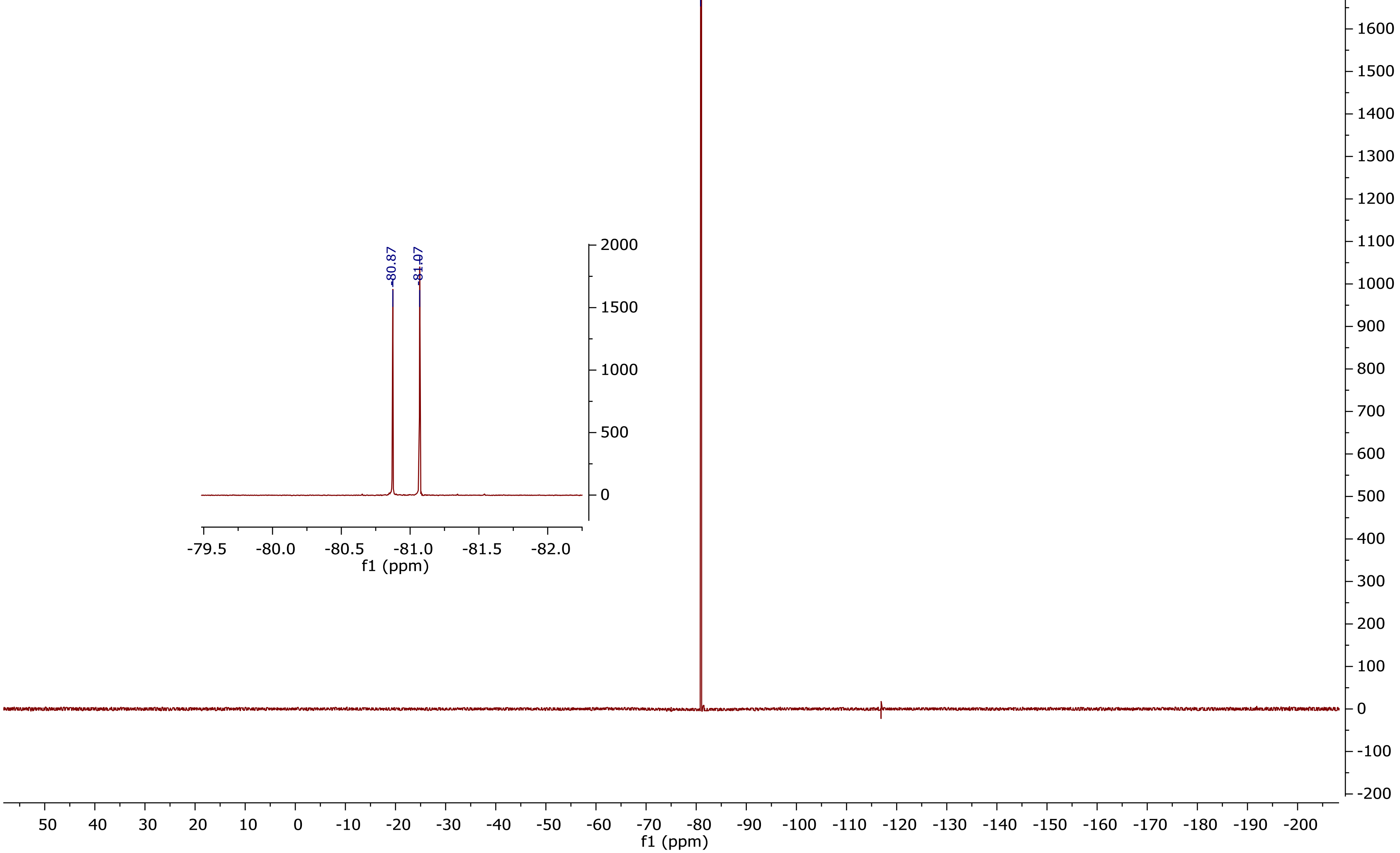

^{1}H NMR (500 MHz, CDCl$_3$) (nementin-12-26)

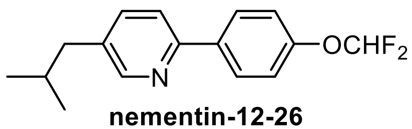

nementin-12-26

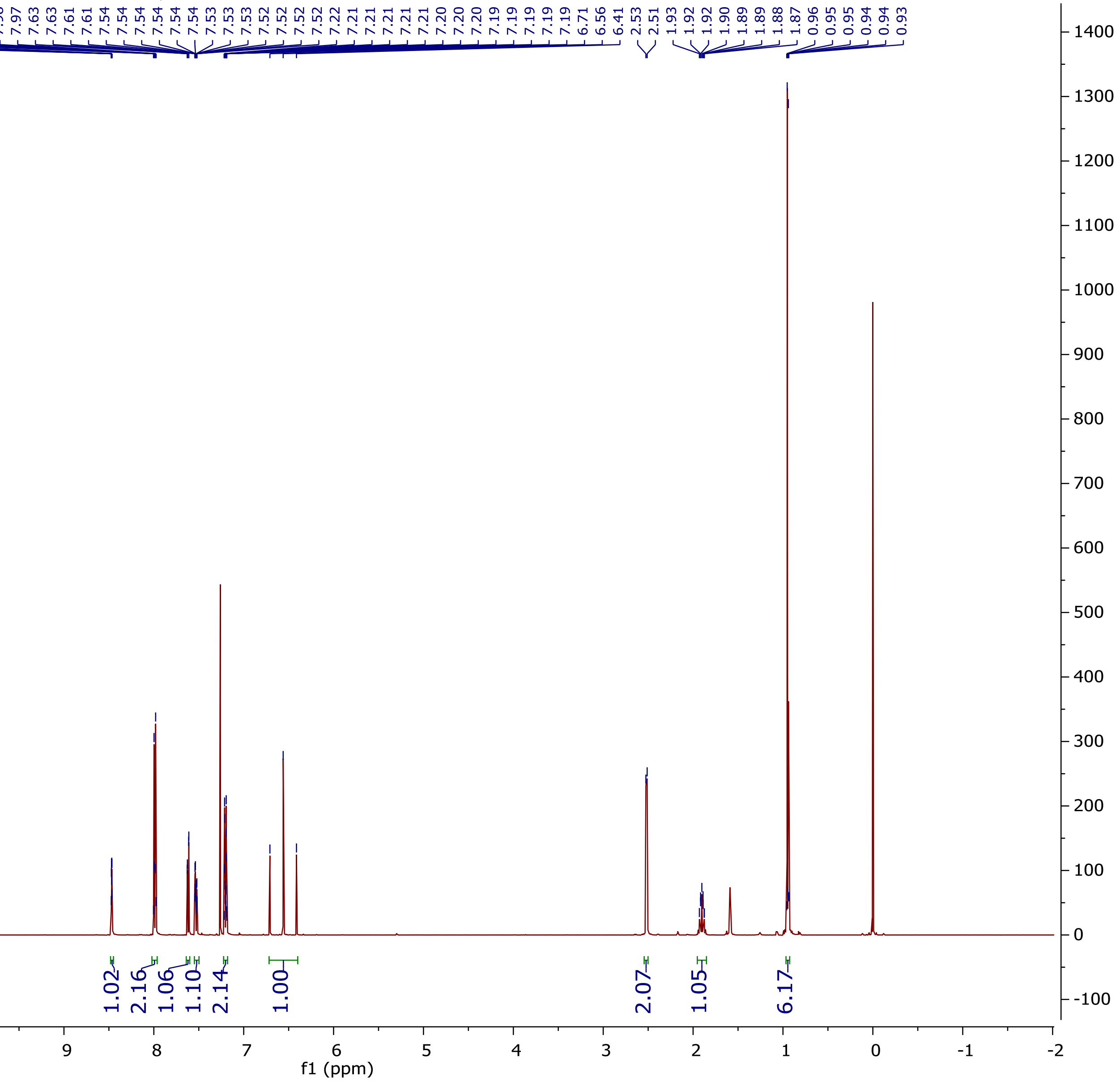

^{13}C NMR (125 MHz, $CDCl_3$)

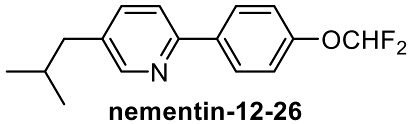

nementin-12-26

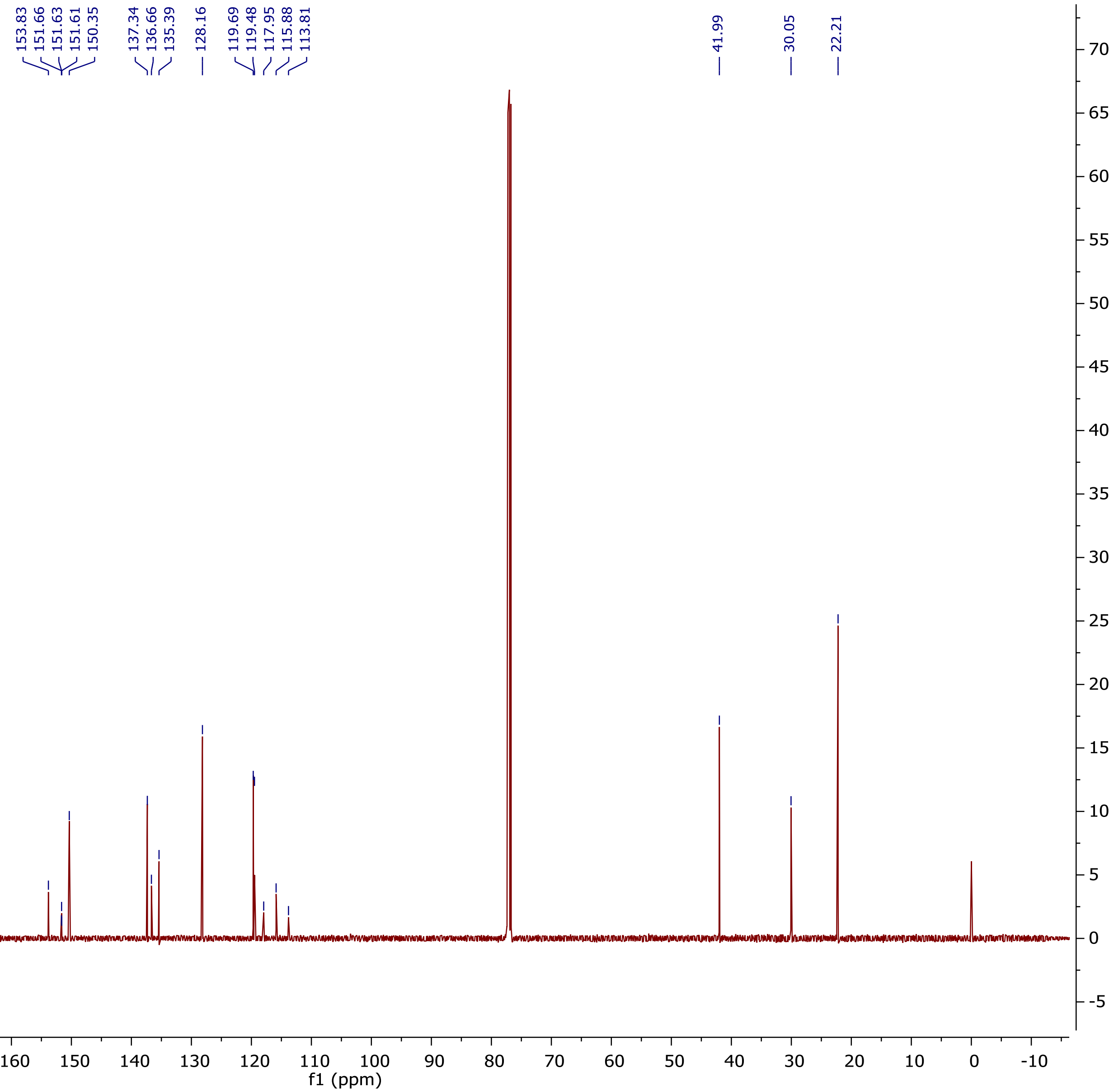

^{19}F NMR (375 MHz, $CDCl_3$)

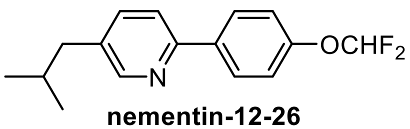

nementin-12-26

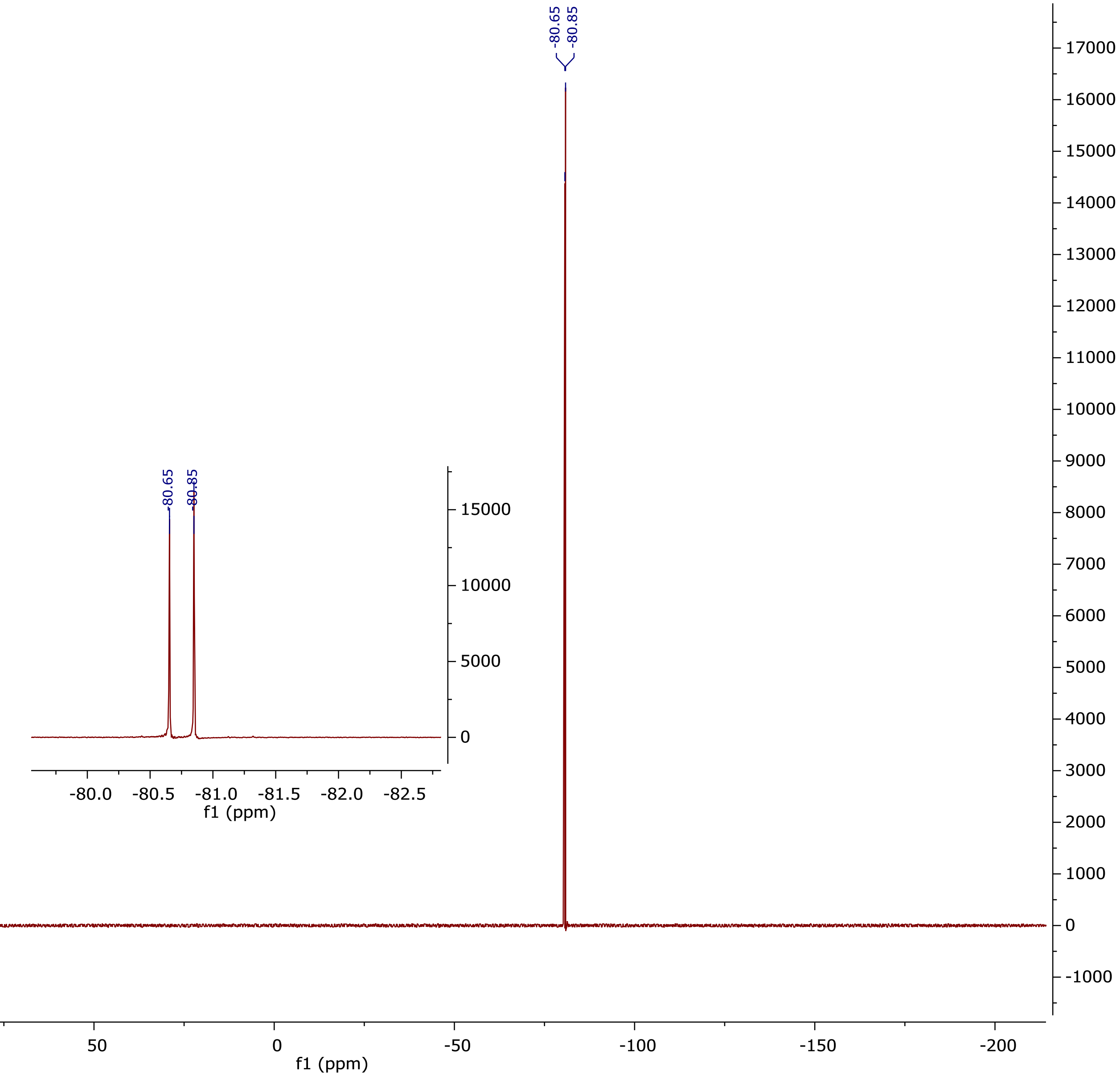

^{1}H NMR (500 MHz, $CDCl_3$) (nementin-12-27)

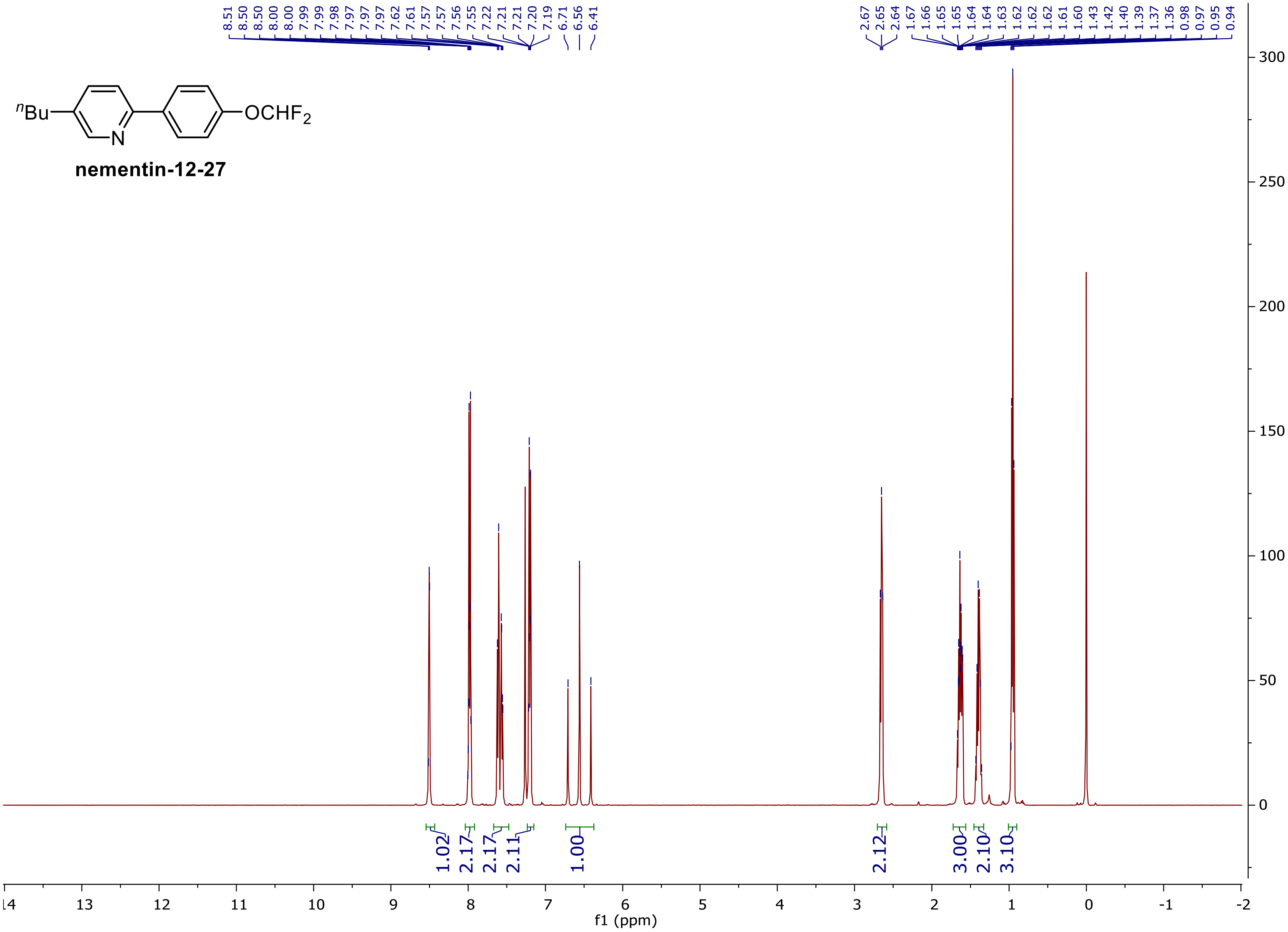

^{13}C NMR (125 MHz, CDCl$_3$)

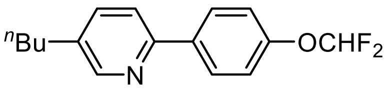

nementin-12-27

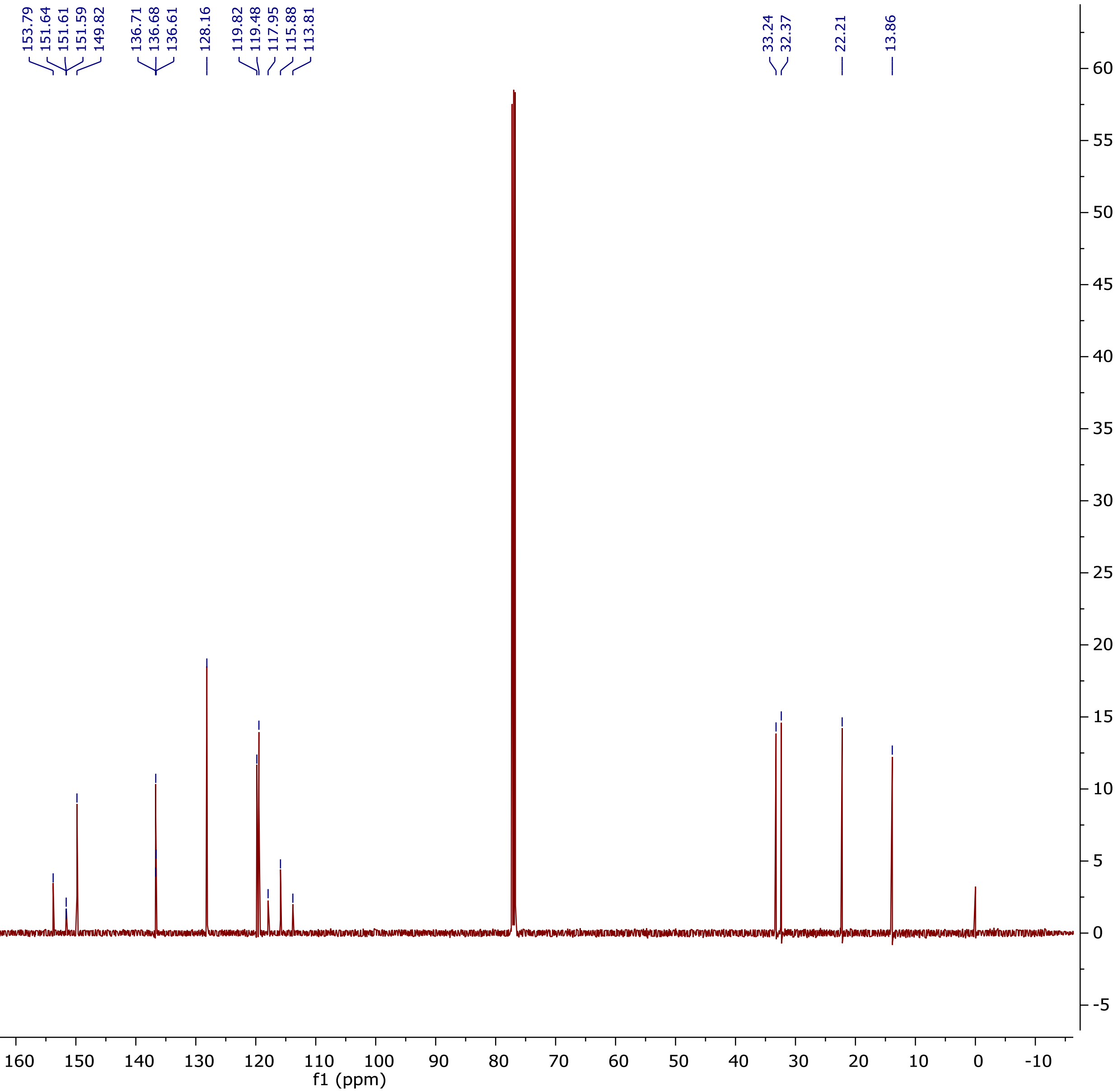

^{19}F NMR (375 MHz, $CDCl_3$)

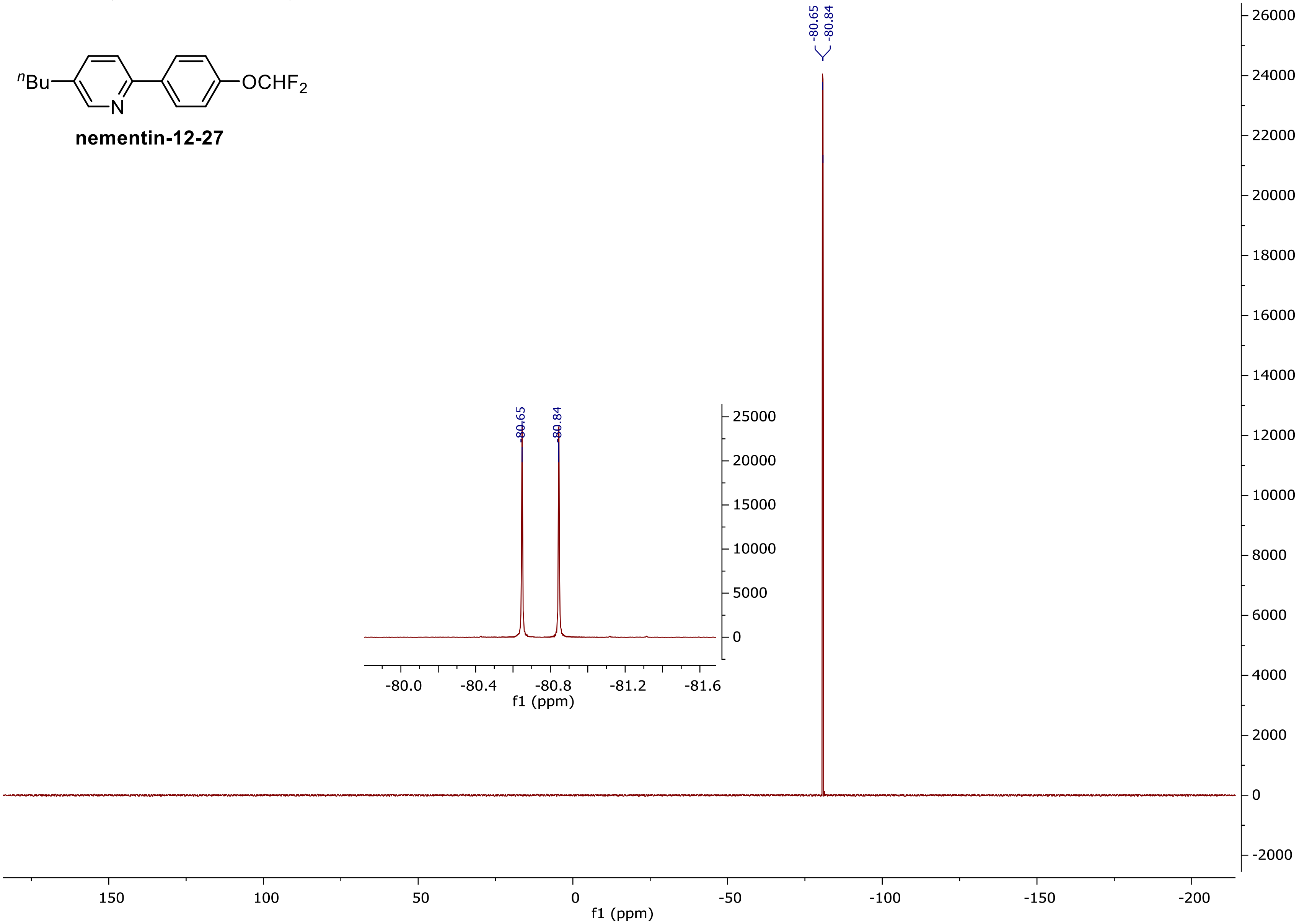

^{1}H NMR (500 MHz, $CDCl_3$) (nementin-12-28)

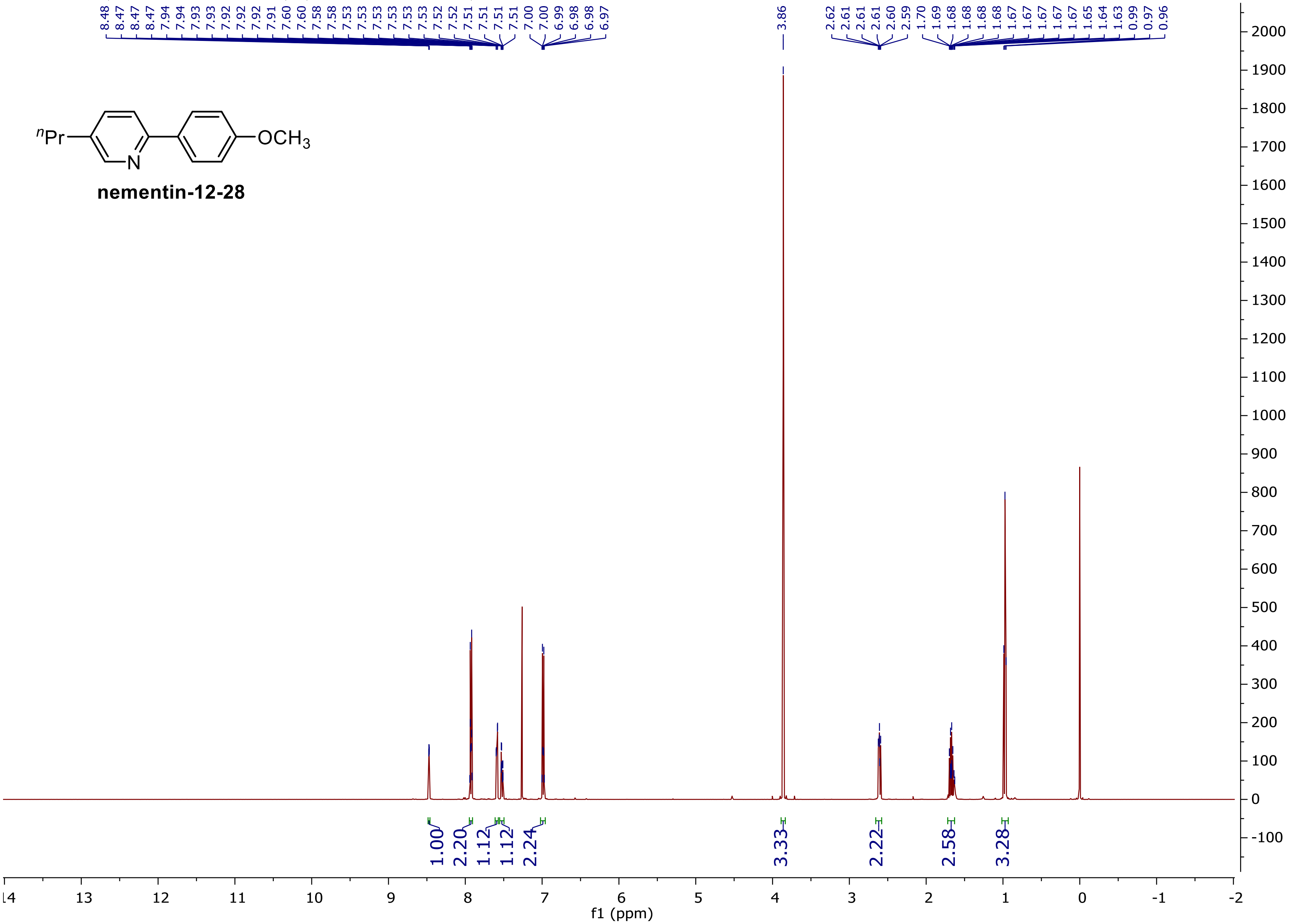

^{13}C NMR (125 MHz, $CDCl_3$)

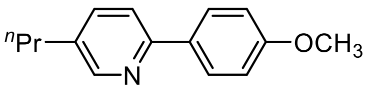

nementin-12-28

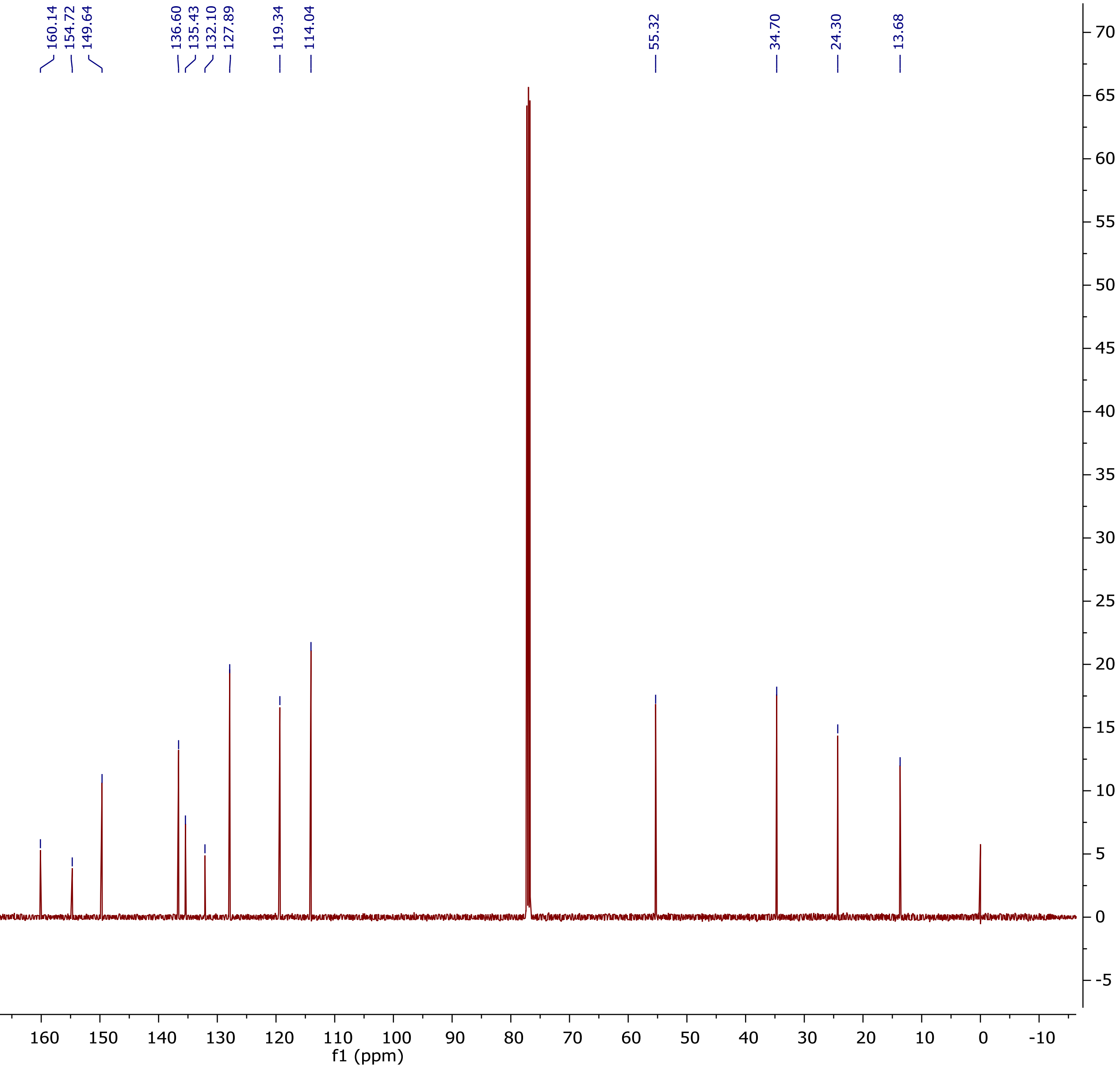

^{1}H NMR (500 MHz, CDCl$_3$) (nementin-12-29)

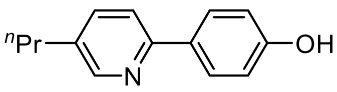

nementin-12-29

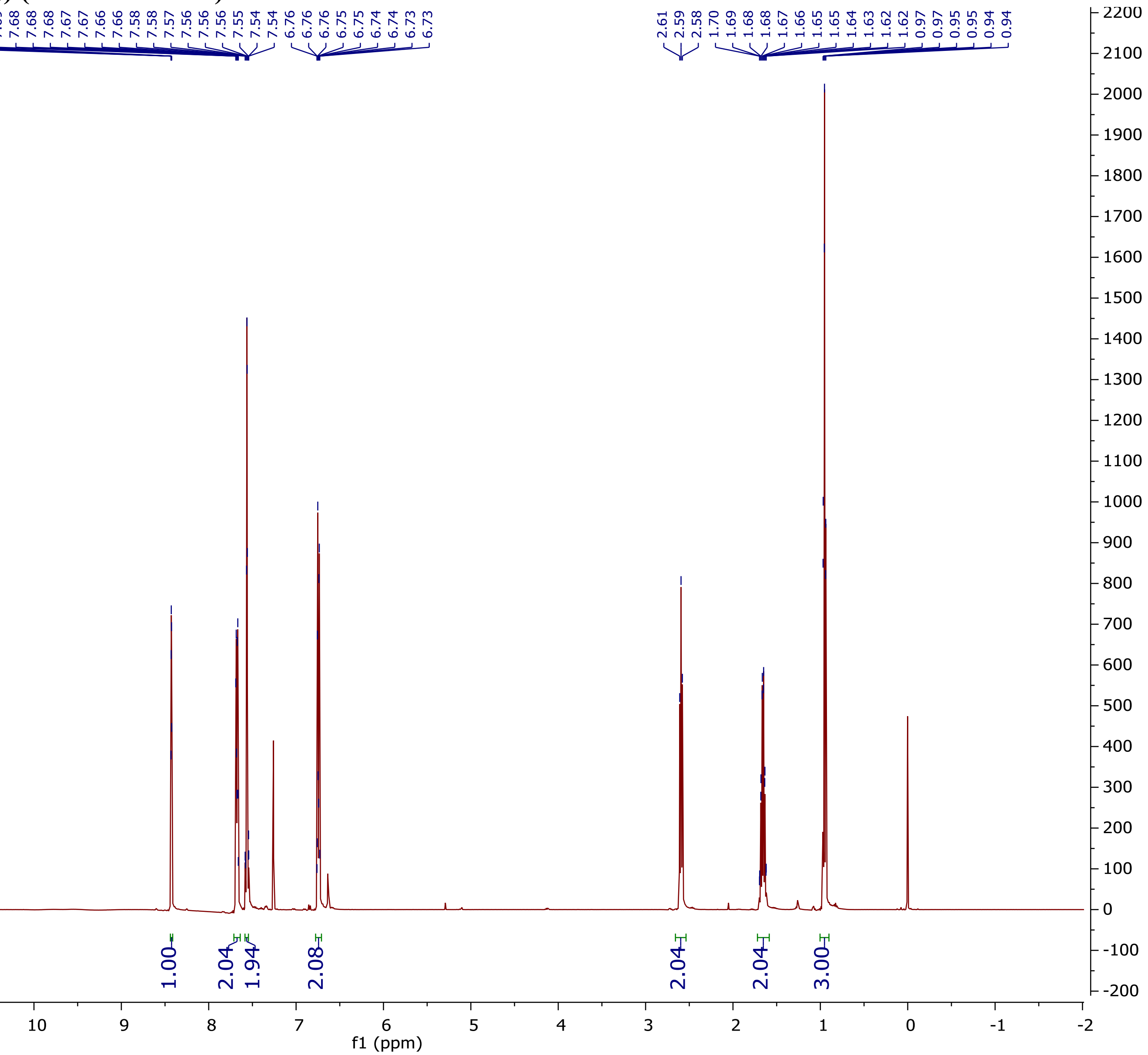

^{13}C NMR (125 MHz, $CDCl_3$)

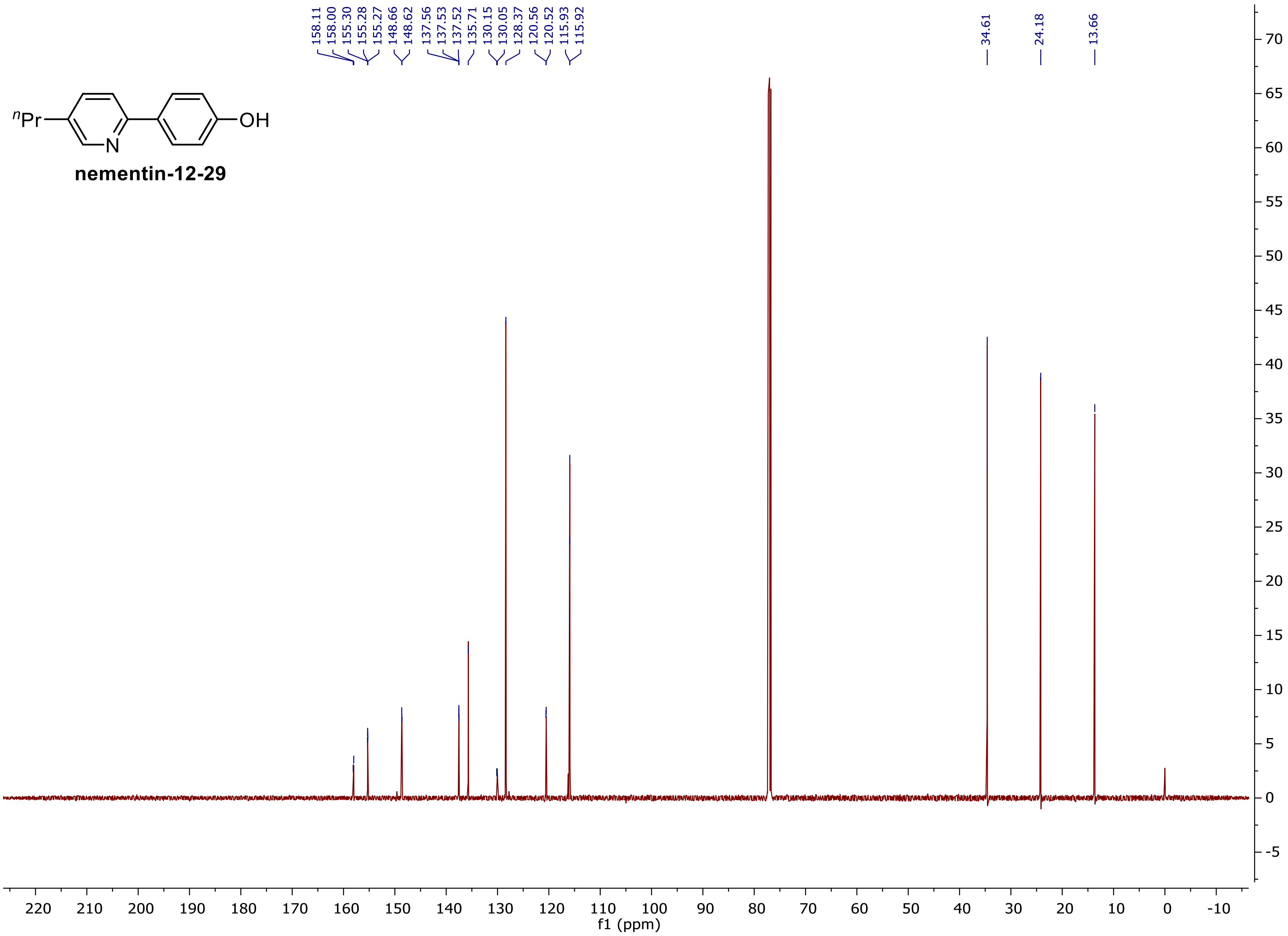

^{1}H NMR (500 MHz, $CDCl_3$) (nementin-12-30)

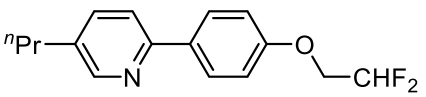

nementin-12-30

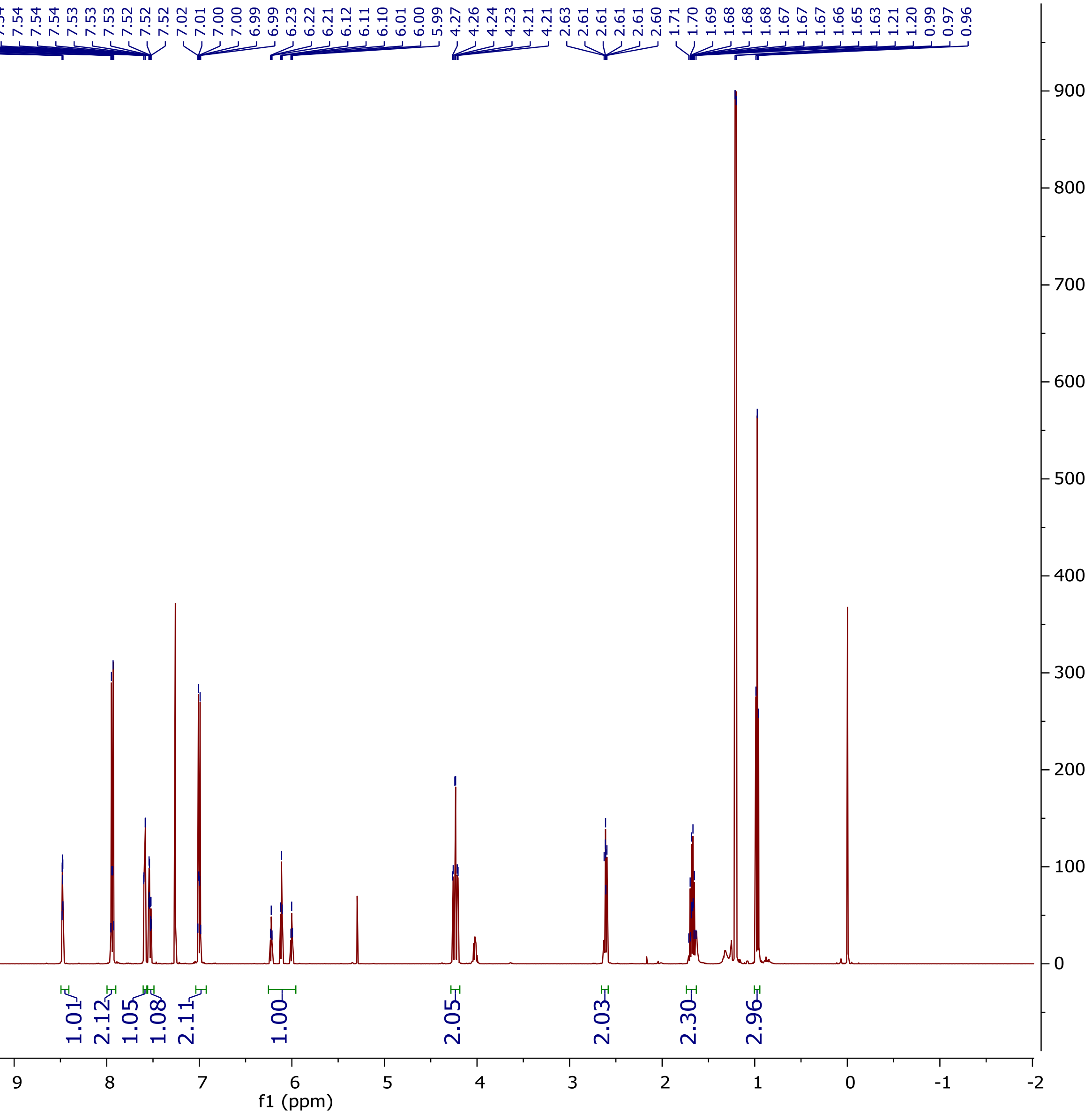

^{13}C NMR (125 MHz, $CDCl_3$)

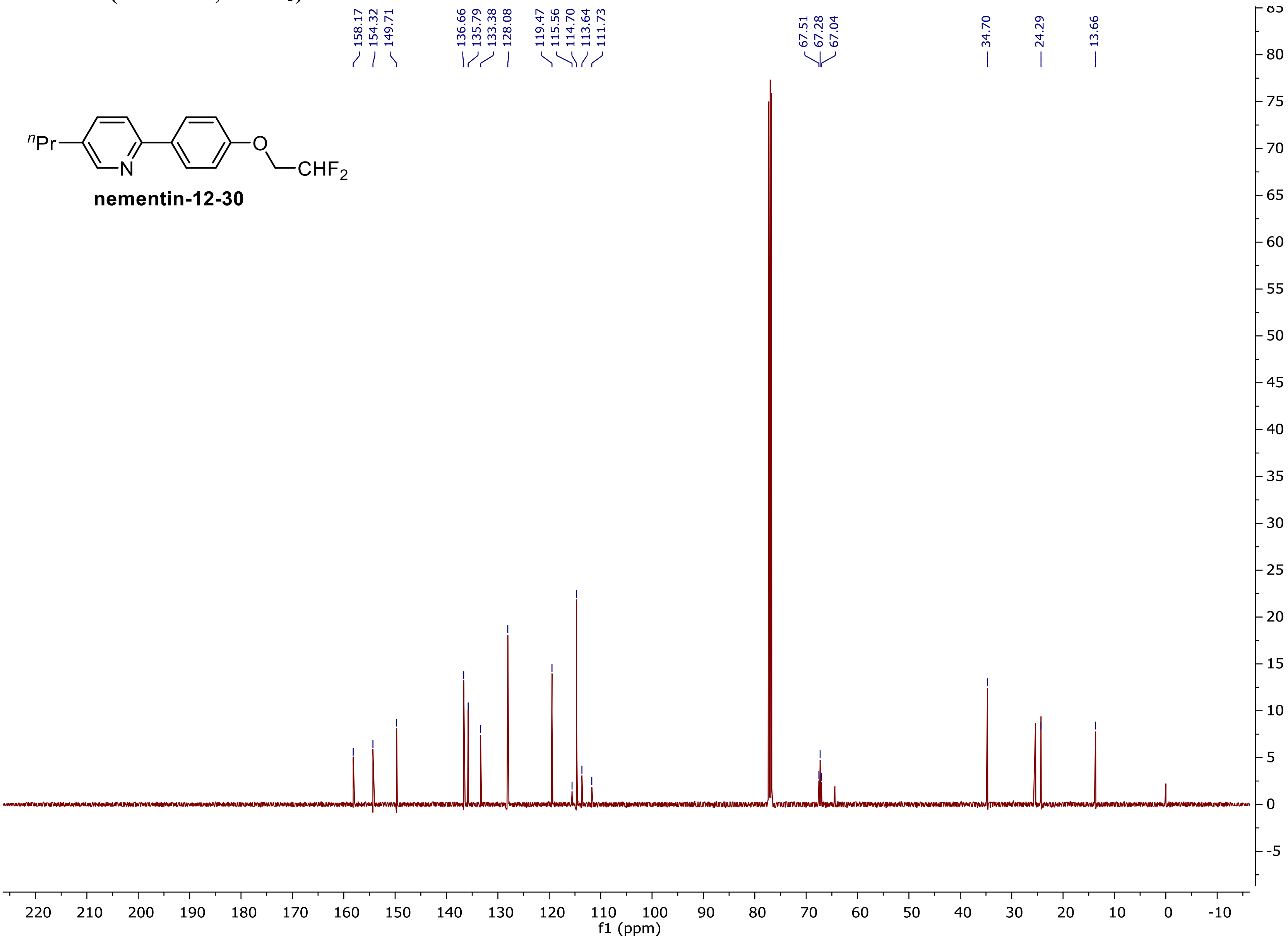

nementin-12-30

^{19}F NMR (375 MHz, $CDCl_3$)

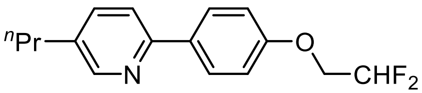

nementin-12-30

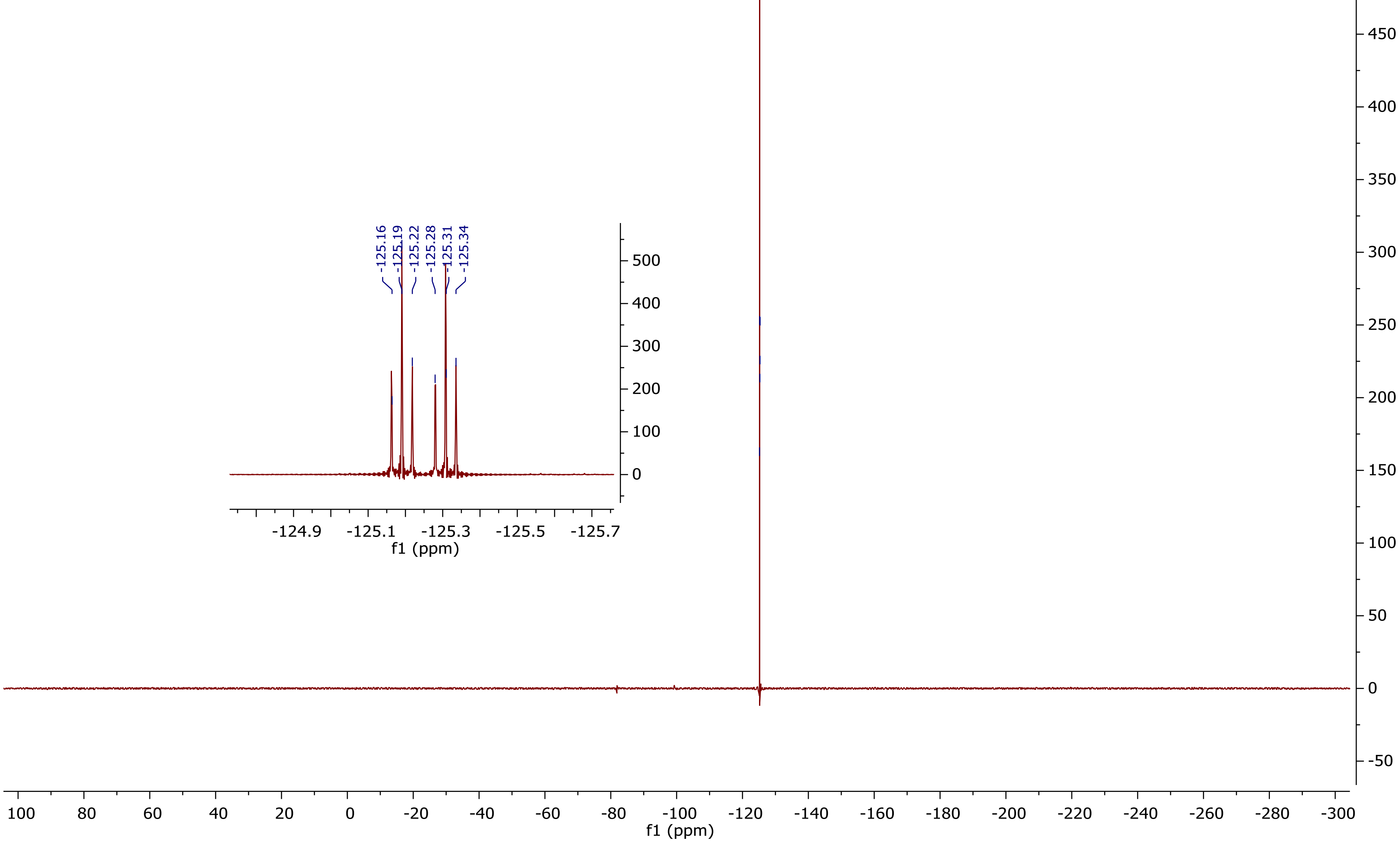

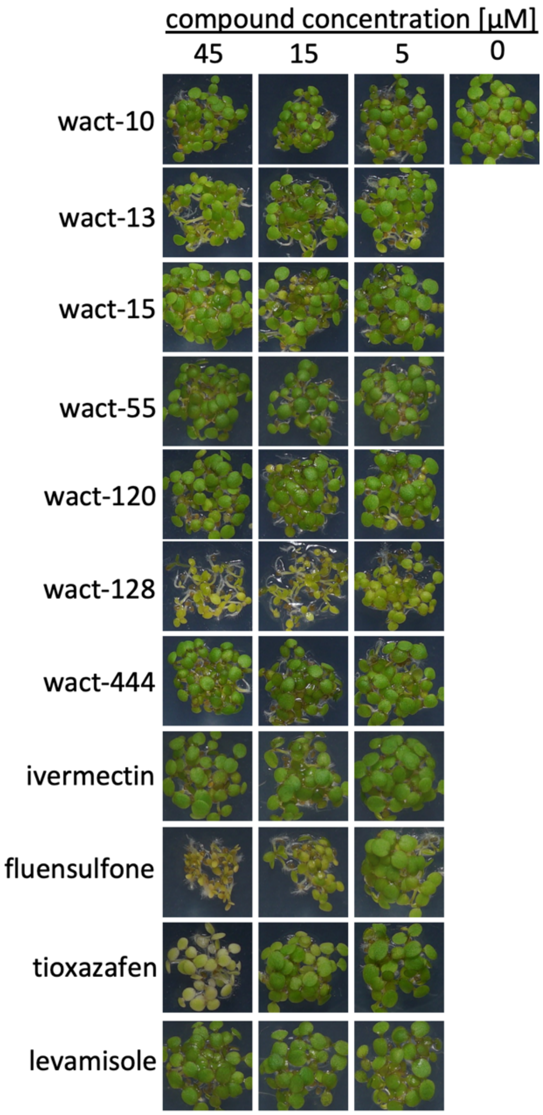

Supplementary Figure 1. Effect of prioritized wactives and established anthelmintics on *Arabidopsis* growth and greening. Assays were performed in 12 well polystyrene plates using 20-25 seeds per well (see Methods). Experiments were performed in triplicate. Any yellowing or obvious growth impairment was reported as activity.

Strain	Resistance	[nementin-1] µM 0	0.94	1.88	3.75	7.5	15	30	60
N2	-	1	1	1.1	1.1	1	0	0	0
VC731 *unc-63(ok1075)*	LEV, APAs, THPs	1	1.1	1.1	1	0.8	0	0	0
CB1072 *unc-29(e1072)*	LEV, THPs	1	1	1.1	1	0.8	0	0	0
DA1316 *avr-14;avr-15;glc-1*	MCL	1	1.1	1	1	0.3	0	0	0
CB3474 *ben-1(e1880)*	Benzimidazoles	1	1	1.1	1.1	0.9	0	0	0
RB2119 *acr-23(ok2804)*	AADs	1	0.9	1.1	1	0.7	0	0	0
NM1968 *slo-1(js379)*	Emodepside	1	1.1	1.1	1	0.5	0	0	0
CF1038 *daf-16(mu86)*	Apigenin	1	1	1	1	0.4	0	0	0
RP2674 *mev-1(tr393)*	Fluopyram	1	1	1.1	0.9	0.6	0	0	0
PR1152 *cha-1(p1152)*	AChE inhibitor	1	1	1	0.9	0.2	0	0	0

Strain	Resistance	[levamisole] µM 0	9.38	18.8	37.5	75	150	300	600
N2	-	1	1	1	1.1	1.1	0.5	0.2	0
VC731 *unc-63(ok1075)*	LEV, APAs, THPs	1	1	1	1	1.1	1.1	1	1
CB1072 *unc-29(e1072)*	LEV, THPs	1	1.1	1.1	1.1	1	1.1	1	1
DA1316 *avr-14;avr-15;glc-1*	MCL	1	0.8	1	1	0.9	0.5	0.1	0
CB3474 *ben-1(e1880)*	Benzimidazoles	1	0.9	1	1.1	0.9	0.6	0.4	0.1
RB2119 *acr-23(ok2804)*	AADs	1	0.9	0.8	0.6	0.5	0.1	0	0
NM1968 *slo-1(js379)*	Emodepside	1	0.9	1	0.9	0.9	0.6	0.1	0
CF1038 *daf-16(mu86)*	Apigenin	1	0.6	0.9	1	0.8	0.7	0.3	0.1
RP2674 *mev-1(tr393)*	Fluopyram	1	0.8	0.9	1	0.9	0.4	0.1	0
PR1152 *cha-1(p1152)*	AChE inhibitor	1	0.3	0.2	0	0	0	0	0

Strain	Resistance	[fluopyram] µM 0	0.09	0.19	0.38	0.75	1.5	3	6
N2	-	1	1.1	1	0.7	0	0	0	0
VC731 *unc-63(ok1075)*	LEV, APAs, THPs	1	1	0.7	0.3	0	0	0	0
CB1072 *unc-29(e1072)*	LEV, THPs	1	0.9	0.7	0.7	0	0	0	0
DA1316 *avr-14;avr-15;glc-1*	MCL	1	1	0.9	0.2	0	0	0	0
CB3474 *ben-1(e1880)*	Benzimidazoles	1	1	0.8	0.7	0	0	0	0
RB2119 *acr-23(ok2804)*	AADs	1	1	1	0.4	0	0	0	0
NM1968 *slo-1(js379)*	Emodepside	1	1	0.6	0	0	0	0	0
CF1038 *daf-16(mu86)*	Apigenin	1	1	1	0.5	0	0	0	0
RP2674 *mev-1(tr393)*	Fluopyram	1	1.1	1.1	1	1	1.1	1	0.9
PR1152 *cha-1(p1152)*	AChE inhibitor	1	1	0.3	0	0	0	0	0

Strain	Resistance	[ivermectin] nM 0	1.88	3.75	7.5	15	30	60	120
N2	-	1	1	1.1	0.8	0.2	0	0	0
VC731 *unc-63(ok1075)*	LEV, APAs, THPs	1	1	1.1	1	0	0	0	0
CB1072 *unc-29(e1072)*	LEV, THPs	1	0.9	1.1	1	0	0	0	0
DA1316 *avr-14;avr-15;glc-1*	MCL	1	1	1.1	1	1	1	0.8	0.4
CB3474 *ben-1(e1880)*	Benzimidazoles	1	1	1	0.7	0.2	0	0	0
RB2119 *acr-23(ok2804)*	AADs	1	1	0.9	1	0.9	0.2	0	0
NM1968 *slo-1(js379)*	Emodepside	1	1	0.8	0.1	0	0	0	0
CF1038 *daf-16(mu86)*	Apigenin	1	1	1	0.9	0	0	0	0
RP2674 *mev-1(tr393)*	Fluopyram	1	0.9	1	1	1	0.2	0	0
PR1152 *cha-1(p1152)*	AChE inhibitor	1	0.9	0.2	0	0	0	0	0

≥ 1 — 0

number of living worms relative to DMSO control

Supplementary Figure 2. *C. elegans* strains with mutant alleles conferring anthelmintic resistance are sensitive to the effects of nementin-1. 3-day larval development assay monitoring the development of larval stage 1 (L1) to L4/adulthood. Reporting the

fraction of animals that develop to L4/adulthood relative to the solvent-only controls. Data are the mean of 3 biological replicates measured in technical triplicate normalized to parallel N2 controls. Showing the strain name and genetic background of parasiticide resistant mutants. Resistance to marketed nematode parasiticides is demonstrated for levamisole (strain VC731 & CB1072), fluopyram (strain RB2674), and ivermectin (DA1316). Primary data for this figure can be found in Supplementary Data 7.

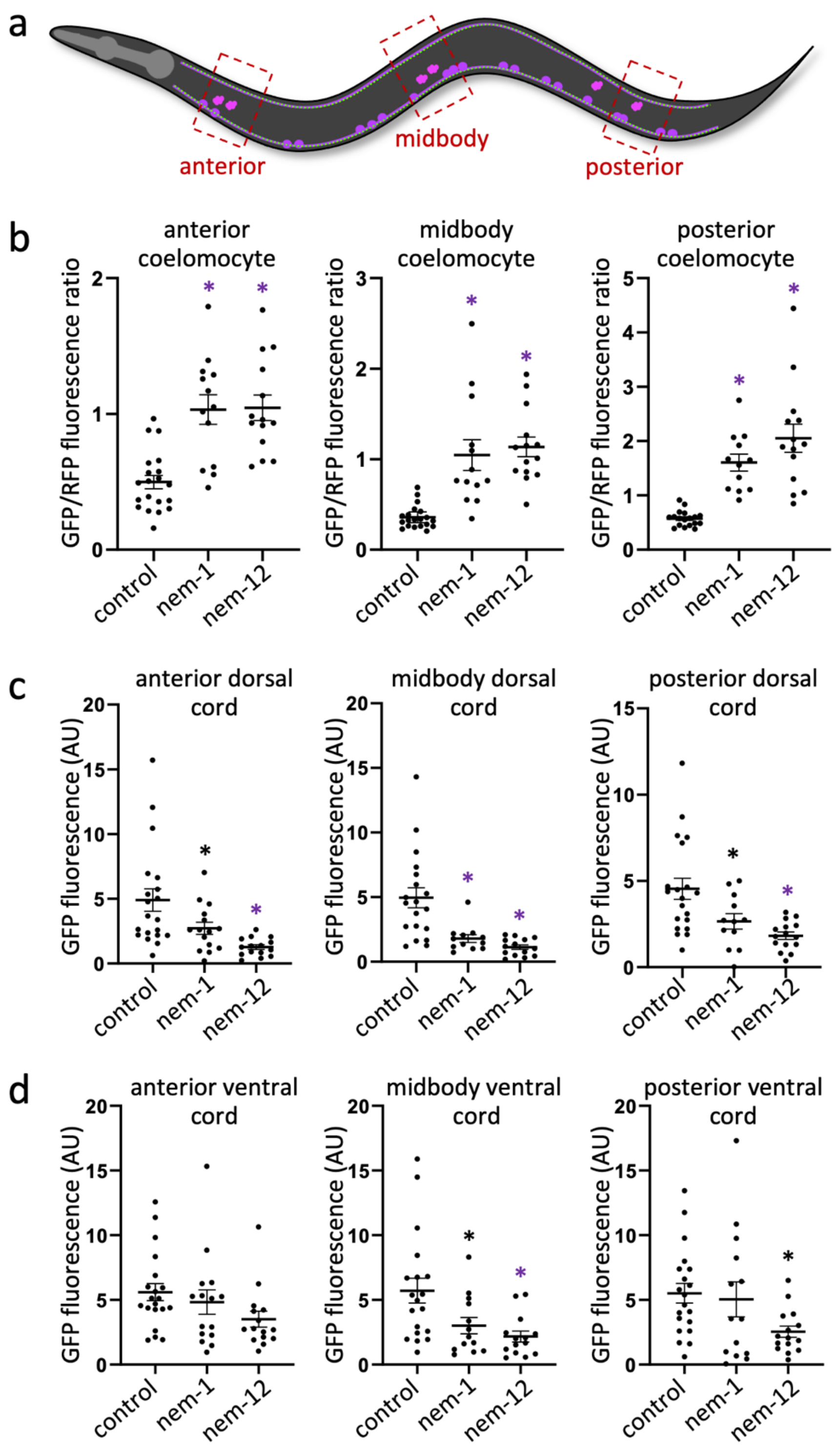

Supplementary Figure 3. Nementin treatment depletes *C. elegans* cholinergic motor neurons of neuropeptides that are released in DCVs. (**a**) Schematic of KG4247 *C. elegans* witih the *ceIs201* integrated transgene expressing INS-22::GFP in

cholinergic motor neurons. INS-22::GFP gets packaged into DCVs serving as a marker for DCVs. The cholinergic motor neurons of KG4247 animals also constitutively secrete mCherry, which is taken up by the coelomocytes, and serves as a coelomocyte marker[4]. Imaged regions are indicated with dashed red boxes. (**b-d**) Quantification of signal from the indicated cell or tissue in the indicated area of the animal. Signal is measured from coelomocytes with a ratio of GFP to RFP fluorescence (because measuring GFP signal alone is too variable because of the variable size of coelomocyte endosomes). Signal is reported from the dorsal and ventral cords as the mean GFP signal with the mean surrounding background signal subtracted. Animals are treated either with DMSO-only (control) or 60 μM of Nementin for 4 hours. For all images, * $p<0.05$;** $p<0.01$; *** $p<0.001$, 1-way ANOVA with Dunnett correction for multiple comparisons; means with SEM are shown. Primary data for this figure can be found in Supplementary Data 3.

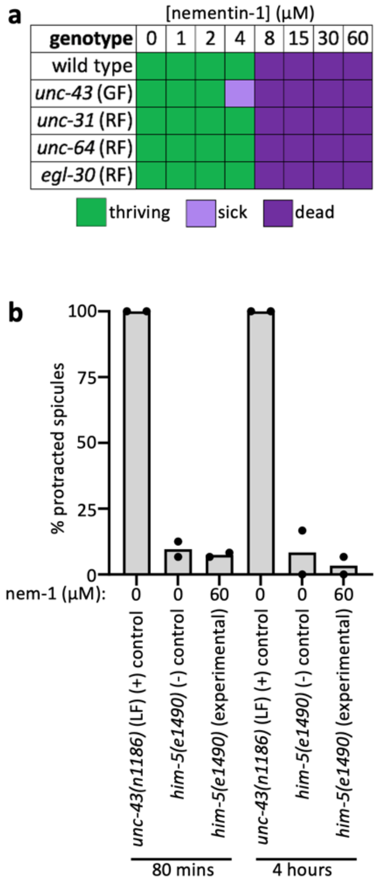

Supplementary Figure 4. UNC-43/CaMKII is unlikely to be the target of nementin.

(**a**) Six-day larval reproduction assay measuring the effects of nementin-1 on generational development. ~20 L1s were plated per well containing 60 μM nementin-1 in NGM media supplemented with HB101 *E. coli* as a food source with 0.6% DMSO. Thriving indicates wells are overgrown with progeny after 6-days. Sick indicates <50 L1 larvae are present per well. Dead indicates <10 live worms are present in test wells. Data are the median response of 3 biological replicates. (**b**) A test to determine whether nementin-1 inhibits UNC-43 in its role in *C. elegans* male tail spicule protraction. The *unc-43* null mutant *(n1186)* is the positive control. Data are the mean of n=2 biologically

independent samples. Primary data for this figure can be found in Supplementary Data 4.

gene	ortholog	exemplar phenocopying mutation and phenotypes[a]	potential suppressing mutation and phenotypes[b]
acr-2	CHRNA1 (cholinergic receptor nicotinic subunit)[5]	*n2420* (GF) convulsions, Egl-c[6,7]	*acr-2(ok1887)* (LF) non-Unc[7]
unc-2	CaV2α (voltage gated calcium channel)[8]	*zf35* (GF) hyperactive, convulsions[9]	*unc-2(e55)* (LF) sluggish[10]
unc-43	CaMKII[11]	*e408* (RF) convulsions, Egl-c [11,12]	*unc-43(n498)* (GF) paralysis[13]
unc-58	KCNK3, KCNK9 and KCNK18 (two-pore K^+ channel)[14]	*e665* (GF) convulsive, Egl-c[14]	*unc-58(e665n273)* (LF) weak Unc[14]
unc-93	UNC93A (SUP-9 K^+ channel complex member)[15]	*e1500* (GF) rubberband[16]	*sup-9(n1012)* (LF) non-Unc[15]
sup-9	two-pore K^+ channel[15]	*n200* (GF) convulsive[16,17]	*sup-9(n1012)* (LF) non-Unc[15]
sup-10	novel (SUP-9 K^+ channel complex member)[15]	*n983* (GF) rubber band[16]	*sup-9(n1012)* (LF) non-Unc[15]
twk-18	TWiK K^+ channel[18]	*e1913* (GF) lethal, rubberband, Egl-d (GF)[19]	*twk-18(gk5009)* (LF) non-Unc (wormbase version WS280)

Supplementary Table 1. Genes that can be mutated to induce convulsions or 'rubber-band' phenotypes. [a]**GF, gain-of-function; RF, reduction-of-function; Egl-c, constitutive egg-laying**

[b]LF, loss-of-function; Unc, uncoordination.

Supplementary References

1 Liu, C., Zhang, Y., Liu, N. and Qiu, J. A simple and efficient approach for the palladium-catalyzed ligand-freeSuzuki reaction in water. *Green Chemistry* **14**, 4 (2012).

2 Geng, Y. *et al.* O-Difluorodeuteromethylation of phenols using difluorocarbene precursors and deuterium oxide. *Org Biomol Chem* **16**, 1807-1811, doi:10.1039/c7ob03088f (2018).

3 Ando, M., Wada, T. & Sato, N. Facile one-pot synthesis of N-difluoromethyl-2-pyridone derivatives. *Org Lett* **8**, 3805-3808, doi:10.1021/ol061481f (2006).

4 Hoover, C. M. *et al.* A novel CaM kinase II pathway controls the location of neuropeptide release from Caenorhabditis elegans motor neurons. *Genetics* **196**, 745-765, doi:10.1534/genetics.113.158568 (2014).

5 Squire, M. D. *et al.* Molecular cloning and functional co-expression of a Caenorhabditis elegans nicotinic acetylcholine receptor subunit (acr-2). *Recept Channels* **3**, 107-115 (1995).

6 Jospin, M. *et al.* A neuronal acetylcholine receptor regulates the balance of muscle excitation and inhibition in Caenorhabditis elegans. *PLoS Biol* **7**, e1000265, doi:10.1371/journal.pbio.1000265 (2009).

7 Petrash, H. A., Philbrook, A., Haburcak, M., Barbagallo, B. & Francis, M. M. ACR-12 ionotropic acetylcholine receptor complexes regulate inhibitory motor

neuron activity in Caenorhabditis elegans. *J Neurosci* **33**, 5524-5532, doi:10.1523/JNEUROSCI.4384-12.2013 (2013).

8 Schafer, W. R. & Kenyon, C. J. A calcium-channel homologue required for adaptation to dopamine and serotonin in Caenorhabditis elegans. *Nature* **375**, 73-78 (1995).

9 Huang, Y. C. *et al.* Gain-of-function mutations in the UNC-2/CaV2alpha channel lead to excitation-dominant synaptic transmission in Caenorhabditis elegans. *Elife* **8**, doi:10.7554/eLife.45905 (2019).

10 Mathews, E. A. *et al.* Critical residues of the Caenorhabditis elegans unc-2 voltage-gated calcium channel that affect behavioral and physiological properties. *J Neurosci* **23**, 6537-6545 (2003).

11 Reiner, D. J., Newton, E. M., Tian, H. & Thomas, J. H. Diverse behavioural defects caused by mutations in Caenorhabditis elegans unc-43 CaM kinase II. *Nature* **402**, 199-203, doi:10.1038/46072 (1999).

12 Brenner, S. The Genetics of Caenorhabditis elegans. *Genetics* **77**, 71-94 (1974).

13 Robatzek, M. & Thomas, J. H. Calcium/calmodulin-dependent protein kinase II regulates Caenorhabditis elegans locomotion in concert with a G(o)/G(q) signaling network. *Genetics* **156**, 1069-1082 (2000).

14 Salkoff, L. *et al.* Potassium channels in C. elegans. *WormBook*, 1-15, doi:10.1895/wormbook.1.42.1 (2005).

15 de la Cruz, I. P., Levin, J. Z., Cummins, C., Anderson, P. & Horvitz, H. R. sup-9, sup-10, and unc-93 may encode components of a two-pore K+ channel that coordinates muscle contraction in Caenorhabditis elegans. *J Neurosci* **23**, 9133-9145 (2003).

16 Greenwald, I. S. & Horvitz, H. R. unc-93(e1500): A behavioral mutant of Caenorhabditis elegans that defines a gene with a wild-type null phenotype. *Genetics* **96**, 147-164 (1980).

17 Levin, J. Z. & Horvitz, H. R. Three new classes of mutations in the Caenorhabditis elegans muscle gene sup-9. *Genetics* **135**, 53-70 (1993).

18 Wei, A., Jegla, T. & Salkoff, L. Eight potassium channel families revealed by the C. elegans genome project. *Neuropharmacology* **35**, 805-829, doi:10.1016/0028-3908(96)00126-8 (1996).

19 Kunkel, M. T., Johnstone, D. B., Thomas, J. H. & Salkoff, L. Mutants of a temperature-sensitive two-P domain potassium channel. *J Neurosci* **20**, 7517-7524 (2000).
