# Supplementary material for: Egg-laying and locomotory screens with C. elegans yield a nematode-selective small molecule stimulator of neurotransmitter release
Source: Commun Biol. 2022 Aug 24;5:865. doi: 10.1038/s42003-022-03819-6 (PMC9402605; doi:10.1038/s42003-022-03819-6)
Supplement: Supplementary file 3 — Description of Additional Supplementary Files [file 42003_2022_3819_MOESM3_ESM.pdf]

Description of Additional Supplementary Files

File name: Supplementary Movie 1.
Description: Example of typical wild type Caenorhabditis elegans locomotion. Wild type C. elegans (strain N2) are swimming on solid media containing only 1% DMSO solvent control that is incorporated into the agar. Animals have been swimming on the plate for 80 minutes at the time the movie was made.

File name: Supplementary Movie 2.
Description: Example of compound-induced convulsions. Wild type C. elegans (strain N2) are shown after swimming on solid media containing 60 µM Nementin-1 for 80 minutes.

File name: Supplementary Movie 3.
Description: Example of compound-induced coiling. Wild type C. elegans (strain N2) are shown after swimming on solid media containing 60 µM wact-45 for 80 minutes.

File name: Supplementary Movie 4.
Description: Example of compound-induced shaking. Wild type C. elegans (strain N2) are shown after swimming on solid media containing 60 µM Nementin-1 for 80 minutes. At the 18 second mark of the movie, C. elegans (strain N2) are shown after swimming on solid media containing 60 µM wact-203 for 80 minutes. At the 21 second mark of the movie, 35 wild type Rhabditophanes diutinus animals are shown after swimming on solid media containing 60 µM Nementin-1 for 170 minutes.

File name: Supplementary Movie 5.
Description: Example of compound-induced jerky-unc phenotype. Wild-type C. elegans (strain N2) are shown after swimming on solid media containing 60 µM wact-45 for 80 minutes.

File name: Supplementary Movie 6. Example of compound-induced reversal-defective phenotype.
Description: Wild type C. elegans (strain N2) are shown after swimming on solid media containing 60 µM wact-38 for 80 minutes.

File name: Supplementary Data 1.
Description: Data for Fig. 1 and Fig. 2

File name: Supplementary Data 2.
Description: Data for Fig. 3

File name: Supplementary Data 3.
Description: Data for Fig. 4 and Supplementary Fig. 3

File name: Supplementary Data 4.
Description: Data for Supplementary Fig 4.

File name: Supplementary Data 5.
Description: Data for Fig. 5

File name: Supplementary Data 6.
Description: Data for Supplementary Fig. 6

File name: Supplementary Data 7.
Description: Data for Supplementary Fig. S2
